# Sequential submodular feature-sample selection for lightweight and update-efficient IoT intrusion detection

**DOI:** 10.1038/s41598-026-66614-x

**Published:** 2026-08-21

**Authors:** Mohammed Nagah Amr, Ahmed Elliethy, Tamer Mekkawy, Ashraf Mahran

**Affiliations:** 1 School of Information Technology (SoIT), Newgiza University, Cairo, 12536 Egypt; 2 Department of Computer Engineering, Military Technical College, Cairo, 11766 Egypt; 3 Department of Avionics, Military Technical College, Cairo, 11766 Egypt; 4 Electronics and Communications Department, Egypt University of Informatics, Cairo, 11741 Egypt

**Keywords:** Intrusion detection systems, Submodular optimization, On-device learning, IoT, Feature selection, Coreset construction, Edge computing, Engineering, Mathematics and computing

## Abstract

The exponential expansion of the Internet of Things (IoT) has created a complex threat landscape that challenges traditional intrusion detection systems (IDS), particularly on edge devices with stringent computational and memory budgets. Beyond lightweight inference, practical IoT security increasingly requires on-device learning and frequent retraining so that models can adapt to evolving traffic patterns and emerging attacks; however, repeatedly training on high-dimensional data and large traffic corpora remains prohibitively expensive for resource-constrained devices. Existing lightweight IDS solutions often treat dimensionality reduction and data pruning as isolated tasks, leading to suboptimal representations and a lack of theoretical guarantees. To address this, we propose the Sequential Submodular Feature-Sample Selection (SSFSS) framework, a theoretically grounded approach that sequentially optimizes the feature and sample spaces to reduce training cost while preserving detection fidelity. First, we introduce Label-Aware Coreset Greedy-Based Feature Selection (LA-CGFS), which formulates feature selection as a label-conditioned facility location problem. This stage maximizes coverage across class manifolds, preserving discriminative power for minority attack classes without relying on synthetic oversampling. Second, we employ a geometry-aware coreset selection strategy that minimizes geometric coverage error in the reduced feature space. By leveraging the submodular property of diminishing returns, SSFSS provides a proven *(1-1/e)* approximation guarantee to the optimal subset. Extensive evaluation on the RT-IoT2022, Edge-IIoTset, and CICIoT2023 datasets demonstrates that SSFSS reduces the feature space to 20 features and the training set to as little as 5% of the original samples, achieving average training speedups of up to *70× * and up to a 96% reduction in peak training RAM; even when the one-time coreset-construction cost is included, the end-to-end pipeline remains faster than a single full-data fit. Across four differentiable empirical-risk-minimization classifiers (kernel logistic regression, kernel SVM, kernel ridge, and softmax regression), the best reduced model retains 97.5–99.5% accuracy at *10%* budget, and the weighted coreset improves macro-F1 and balanced accuracy on the imbalanced benchmarks. Supported by the reported results, we position the proposed SSFSS framework as an efficient update-aware preprocessing framework whose measured speedups and RAM consumption motivate deployment on resource-constrained edge hardware.

## Introduction

The rapid expansion of the Internet of Things (IoT) has made connected sensing, monitoring, and control fundamental to Industry 4.0, smart healthcare, intelligent transportation, and critical infrastructure^1,2^. With the number of connected devices continuing to grow at a massive scale, IoT deployments increasingly push computation and decision making toward the network edge, where latency, bandwidth, and autonomy constraints make local intelligence highly desirable^3–5^. This same proliferation, however, has also enlarged the attack surface of cyber-physical systems. Securing IoT environments is especially challenging because many devices and edge gateways operate under strict limits on CPU cycles, memory, storage, and energy, which restrict the applicability of conventional security mechanisms designed for server-class platforms^4,5^.

Intrusion Detection Systems (IDS) therefore remain a central defensive layer in IoT security architectures^6,7^. Signature-based IDS are highly effective against previously seen attacks, but their dependence on known patterns limits their usefulness against zero-day threats and rapidly evolving adversarial behavior. In contrast, anomaly-based IDS built on machine learning (ML) and deep learning (DL) offer stronger generalization to unseen attacks, yet they typically require training on high-dimensional traffic representations and large data corpora, which substantially increases computational and memory cost^8,9^. For resource-constrained IoT environments, this challenge extends beyond lightweight inference. In practice, an IDS should also support *update-efficient learning*: models should be retrainable or refinable as device behavior changes, traffic distributions drift, software is updated, and new attack patterns emerge. Repeatedly training on the full feature space and the full historical dataset, however, is generally infeasible on constrained edge hardware^10–13^.

This requirement shifts the design objective for IoT-IDS from lightweight prediction alone to efficiency across the full learning lifecycle. For practical edge security, lightweight inference alone is not sufficient: IoT traffic distributions evolve over time, device behavior changes with software updates and operational context, and attack patterns continuously adapt. Consequently, an effective IDS should be deployable on constrained hardware while also supporting periodic local retraining, online refinement, or participation in distributed update schemes (such as federated learning updates) without prohibitive cost^10–13^. Crucially, a model that is light enough for inference may still remain too expensive to retrain frequently when new local traffic becomes available, leaving a persistent gap between deployment feasibility and update efficiency.

Recent research has concentrated on reducing costs, primarily through data reduction techniques. Feature selection methods seek to eliminate redundant and weakly informative traffic attributes, thereby ensuring that sensing, storage, and learning are feasible on constrained devices^14–16^. At the same time, sample handling methods aim to rebalance or compress training datasets using techniques such as oversampling, pruning, or selective sampling^12,17–19^. These strategies not only enhance computational efficiency but also improve the overall performance of machine learning models in resource-limited environments. Consequently, integrating these innovative approaches is becoming increasingly essential for advancing the capabilities of IoT devices and edge computing applications. Submodular optimization^20–23^ serves as a natural link between these two data-reduction perspectives, as both feature selection and sample selection can be framed as budgeted subset-selection problems. The diminishing-returns property of submodular objectives favors elements that provide complementary information over those with only high individual relevance, making it suitable for selecting compact and representative feature and sample subsets. This method is particularly attractive in resource-constrained IoT-IDS settings, where the goal is not only to reduce dimensionality or the training set size in isolation but also to maintain the informative structure necessary for reliable detection within strict memory and computational limits.

### Motivation

A key limitation in current literature is that feature reduction and sample reduction are often executed using disparate techniques, each with its own objectives, heuristics, and optimization criteria. This inconsistency in methodology prevents the overall pipeline from leveraging the unified theoretical guarantees offered by submodular optimization,^24^. It also complicates rigorous end-to-end error analysis, as the approximations introduced during feature selection cannot be seamlessly integrated into the subsequent sample selection phase (or vice versa). Conversely, formulating both feature selection and sample selection as submodular functions, as proposed in the SSFSS framework, allows for a coherent overall error analysis while maintaining theoretical consistency across the two sequential stages. Moreover, many traditional feature-selection methods depend on global relevance scores that may underrepresent minority attack structures, while various sample handling approaches either increase the effective training set through synthetic oversampling or rely on heuristic pruning, which lacks robust theoretical assurances about how faithfully the selected subset mimics full-dataset training^14,18,19,21–23^. Consequently, existing lightweight IDS pipelines may enhance deployment efficiency but do not adequately address the primary challenge for adaptive edge security: how to retrain accurately and frequently under stringent memory and time constraints.

These observations motivate a theoretically grounded preprocessing framework that reduces training cost while preserving discriminative structure. In this paper, we propose the **Sequential Submodular Feature-Sample Selection (SSFSS)** framework, which sequentially optimizes the feature and sample spaces for lightweight IoT intrusion detection. In the first stage, Label-Aware Coreset Greedy-Based Feature Selection (LA-CGFS) formulates feature selection as a label-conditioned facility location problem so that the reduced representation preserves coverage across both majority and minority class manifolds. In the second stage, SSFSS performs gradient-aware sample reduction in that reduced feature space to construct a compact training subset that remains faithful to the optimization behavior of the full dataset. By leveraging monotone submodular optimization, the framework inherits the classical greedy approximation guarantee of *(1-1/e)* under the adopted objective^24^. Consequently, SSFSS is intended not merely as an offline compression technique, but as a key enabler for practical on-device learning and frequent retraining in resource-constrained IoT environments.

### Our contributions

The main contributions of this paper are summarized as follows:

- We propose SSFSS, a sequential feature–sample selection framework for lightweight IoT-IDS that is explicitly designed to support update-efficient learning, including on-device adaptation and frequent retraining under stringent resource constraints.
- We introduce LA-CGFS, a label-aware feature selection method that preserves class-discriminative coverage across traffic manifolds and mitigates the tendency of conventional global scoring methods to overlook minority attack characteristics.
- We develop a gradient-aware sample reduction strategy in the reduced representation space and provide a rigorous theoretical analysis for the complete sequential pipeline, including submodular approximation guarantees and an error characterization that links feature reduction, coreset construction, and optimization fidelity^20–23^.
- We evaluate SSFSS on RT-IoT2022, Edge-IIoTset, and CICIoT2023 using four differentiable ERM classifiers (KLR, KSVM, KRR, SoftmaxLR) over a shared Nyström feature map, showing that the framework reduces the feature space to 20 features and the training set to 5–15% of its original size while the best reduced classifier retains 97.5–99.5% accuracy at the balanced *10%* budget, achieving average training speedups of up to *70× * and up to a 96% reduction in peak training RAM.

The literature review in Section “Literature review” is organized around the research threads most relevant to this objective: lightweight IoT-IDS and update-efficient learning, feature selection for resource-constrained IDS, sample handling under class imbalance, and submodular/coreset-based data-efficient training, followed by a synthesis of the resulting research gap. This organization clarifies why existing lightweight IDS pipelines remain insufficient for adaptive edge security and positions SSFSS within the broader literature. Section “The proposed sequential submodular feature-sample selection (SSFSS) framework” then presents the proposed framework, Section “Error analysis” develops the theoretical guarantees, Section “Experimental evaluation” reports the empirical evaluation, and Section “Conclusion and future work” concludes the paper.

## Literature review

Recent IoT intrusion detection research has progressively shifted from cloud-centric, accuracy-driven models toward resource-aware pipelines that can operate close to the data source. Contemporary surveys consistently identify the same central challenge: an IoT-IDS must preserve detection fidelity while respecting tight limits on memory, computation, energy, and latency on edge or embedded platforms^6,7^. For practical edge security, lightweight inference alone is insufficient. Because IoT traffic, device behavior, and attack patterns evolve over time, an effective IDS must be not only deployable on constrained hardware, but also update-efficient enough to support local retraining, online refinement, or distributed updates without prohibitive cost. This broader requirement motivates a literature review that goes beyond accuracy-centric comparisons and instead focuses on whether existing preprocessing strategies are suitable for update-efficient learning.

### Lightweight IoT-IDS and the need for update-efficient learning

A large body of work has reduced IoT-IDS cost through compact architectures, hardware-aware deployment, and multi-stage preprocessing. Recent IDS studies continue to improve detection quality through hybrid deep models and increasingly elaborate balancing pipelines^10,11^, while lightweight frameworks such as LIRAD explicitly optimize memory footprint and execution time for constrained platforms^12^. TinyML-oriented research pushes this direction further by demonstrating intrusion detection on microcontroller-class hardware^13^. These efforts collectively show that edge deployment is feasible, but they also expose a more subtle limitation: A model may be lightweight for inference yet still too costly to retrain frequently as new local traffic becomes available. This distinction is especially important in IoT security, where adaptation speed can be as important as static test accuracy.

### Feature selection for resource-constrained IoT-IDS

Feature selection remains the dominant mechanism for lowering the computational burden of IoT-IDS because network-flow datasets often contain dozens of correlated, noisy, or weakly informative attributes. Filter-based methods are particularly attractive for constrained environments due to their simplicity and low preprocessing cost. Musthafa et al.^9^ showed that ANOVA-based feature pruning can substantially reduce input dimensionality while preserving strong performance in ensemble-based IDS models. Narayan et al.^15^ similarly demonstrated that correlation-based subset selection can improve multiclass detection on CICIoT2023, while Amierh et al.^16^ used variance-threshold screening to build a compact multiclass IoT-IDS pipeline. Taken together, these studies confirm that significant redundancy exists in contemporary IoT traffic representations and that substantial compression is often possible.

Beyond simple statistical filters, wrapper, embedded, and metaheuristic methods attempt to capture higher-order dependencies among features. LEMDA^25^ estimates feature importance through the decay of predictive performance, whereas Houkan et al.^26^ report that mRMR-style criteria can outperform generic linear reduction methods such as PCA in industrial IoT settings. Optimization-driven approaches such as GSLDWOA^27^, genetic/synergistic search strategies^28^, and cached wrapper evaluation^29^ further improve the accuracy–efficiency trade-off by exploring the combinatorial feature space more aggressively. Nevertheless, most existing feature-selection techniques are primarily validated for one-shot offline training and often rely on global relevance scores. As a result, they provide limited guarantees that the reduced representation will preserve class-conditional structure—especially for minority or high-impact attacks—during repeated retraining on-device^14–16^.

### From class rebalancing to sample compression

A second major line of work addresses the severe class imbalance that characterizes IoT intrusion datasets. The dominant approach remains synthetic oversampling or hybrid resampling. SMOTE-based pipelines are widely used in IoT-IDS^16,30^, while SMOTETomek^18^, DCGAN-based augmentation^31^, and dynamic weighting mechanisms^32^ seek to improve minority-class recall under extreme skew. More recent frameworks such as AegisGuard^19^ combine multi-stage resampling with feature selection to stabilize performance across heterogeneous IIoT traffic. These methods are valuable for correcting biased class distributions, but they do not directly address the computational burden of repeated model updates. In fact, oversampling commonly enlarges the effective training set, which is counterproductive when the goal is frequent retraining under strict memory and time budgets.

To reduce training cost more directly, several recent studies have begun exploring selective sampling and compact training subsets. Javed et al.^17^ investigate active sample selection to remove redundant traffic instances, and Mishra et al.^12^ integrate sampling, feature selection, and pruning to improve constrained deployment. Hybrid preprocessing pipelines such as the two-step strategy of Gheni et al.^14^ further show that data reduction choices strongly influence downstream IDS efficiency. However, most existing sample-reduction approaches in IoT-IDS remain heuristic. They rarely provide a formal account of how well the selected subset preserves the geometry of the original data, the discriminative information in the training set, or the gradients that drive optimization. This limitation becomes critical when the reduced subset is expected to support on-device learning, online refinement, or frequent retraining rather than merely one-time offline model fitting.

### Submodular selection and coreset-based data-efficient training

Compared with the IoT-IDS literature, data-efficient learning in machine learning has a stronger theoretical foundation in submodular optimization and coreset construction. The results in literature show that greedy algorithms achieve a *(1-1/e)* approximation for monotone submodular objectives^24^, making submodularity particularly attractive for representative subset selection under budget constraints. In parallel, coreset theory formalizes how a small weighted summary can preserve key geometric or optimization properties of a much larger dataset^20,21^. These ideas are especially relevant when the main bottleneck is training cost rather than inference cost.

Recent machine learning work has translated these principles into gradient-aware data selection. CRAIG formulates subset selection as a submodular problem that approximates the full gradient and provides convergence guarantees for incremental gradient methods^22^. GRAD-MATCH extends this perspective by constructing subsets that approximate training or validation gradients with explicit optimization guarantees^23^. Although these methods were not originally proposed for IoT intrusion detection, they identify precisely the kind of guarantee that adaptive edge security requires: the reduced subset should be compact, representative, and faithful to the optimization induced by the full dataset. Yet, in current IoT-IDS practice, these theoretically grounded ideas are rarely integrated with feature reduction in a unified preprocessing pipeline.

### Research gap and positioning of SSFSS

The literature therefore reveals a persistent gap between existing lightweight IDS methods and the requirements of adaptive edge security. Existing feature-selection studies reduce dimensionality but are usually not explicitly label-aware in a manner that preserves minority-class coverage. Existing class-imbalance solutions improve recall but frequently enlarge the dataset instead of compressing it. Existing sample-selection methods reduce data volume but seldom provide principled guarantees that the selected subset remains faithful to the full optimization problem. Most importantly, the primary constraint in the existing literature is that feature reduction and sample reduction are generally executed using disparate heterogeneous methods, each possessing distinct objectives, heuristics, and optimization criteria. This methodological inconsistency obstructs the overall pipeline from leveraging the cohesive theoretical guarantees inherent in submodular optimization^24^, and it also impedes a thorough end-to-end error analysis, as the approximation introduced during the feature-selection phase cannot be seamlessly transmitted into the subsequent sample-selection phase (or vice versa).

The proposed Sequential Submodular Feature-Sample Selection (SSFSS) framework is designed to bridge this gap. SSFSS first performs label-aware feature selection so that the reduced representation preserves discriminative structure across normal and attack classes, and then performs gradient-aware sample reduction in that representation to construct a compact training coreset. This positioning places the present work at the intersection of lightweight IoT-IDS, imbalance-aware multiclass detection, and theoretically grounded data-efficient learning. Unlike prior pipelines based only on global feature scoring, synthetic oversampling, or heuristic resampling^15,16,18,19^, SSFSS explicitly targets the core requirement highlighted in this paper: enabling practical on-device learning and frequent retraining on resource-constrained IoT devices while retaining strong representational and optimization fidelity.

## The proposed sequential submodular feature-sample selection (SSFSS) framework

**Fig. 1:**
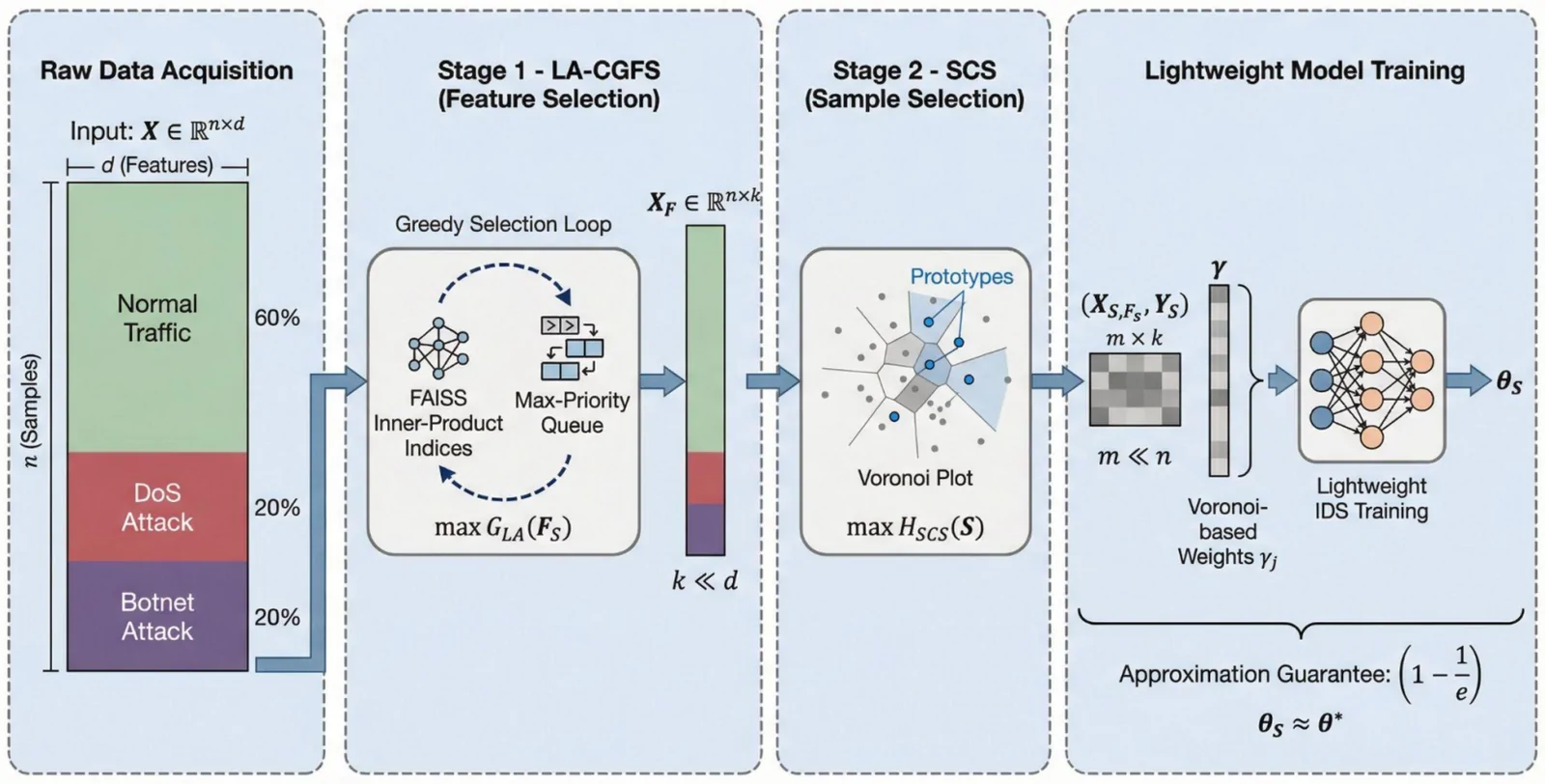
The architectural flow of the proposed SSFSS framework, illustrating the sequential optimization of the feature space (Stage 1: LA-CGFS via Label-Conditioned Facility Location submodular maximization with FAISS and Lazy Greedy) followed by the sample space (Stage 2: SCS via gradient-aware coreset selection with Voronoi-based weighting), transforming raw IoT IDS data $\boldsymbol{X} \in \mathbb {R}^{n \times d}$ into a lightweight weighted coreset of size *m × k* for efficient classifier training.

The proposed Sequential Submodular Feature-Sample Selection (SSFSS) framework is designed to make lightweight IDS training practical under the stringent memory and computational limits of IoT edge environments. Rather than optimizing inference alone, SSFSS targets the broader requirement of *update-efficient learning*: the ability to retrain or refine an IDS model frequently as traffic distributions evolve, without repeatedly processing the full high-dimensional dataset. To this end, SSFSS applies a sequential reduction pipeline. Stage 1 performs label-aware feature selection to preserve class-discriminative structure, particularly for minority attack classes, and Stage 2 performs gradient-aware sample reduction in the resulting feature space to construct a compact weighted coreset for downstream training. The overall workflow is illustrated in Fig. 1.

### Preliminaries and notation

Let the supervised training dataset be denoted by

$$ \mathcal {D} = \{(\boldsymbol{x}_i, y_i)\}_{i=1}^{n}, $$

where $\boldsymbol{x}_i \in \mathbb {R}^{d}$ is the *i*^th^ training sample and $y_i \in \{1,\dots ,C\}$ is its class label. Let $\boldsymbol{X} \in \mathbb {R}^{n \times d}$ be the corresponding data matrix, $\mathcal {I}=\{1,\dots ,n\}$ the set of sample indices, and $\mathcal {F}=\{1,\dots ,d\}$ the set of feature indices. For each feature index $j \in \mathcal {F}$, let $\boldsymbol{f}_j = \boldsymbol{X}_{:,j} \in \mathbb {R}^{n}$ denote the $j^{\text {th}}$ feature column. SSFSS seeks a feature subset $F_s \subseteq \mathcal {F}$ with $|F_s| = k \ll d$ and a sample subset $\mathcal {S} \subseteq \mathcal {I}$ with $|\mathcal {S}| = m \ll n$.

The output of the framework is the reduced training dataset

$$ (\boldsymbol{X}_{\mathcal {S},F_s},\, \boldsymbol{Y}_{\mathcal {S}}), $$

which is used by the downstream IDS learner in place of the full training set.

For any subset $\mathcal {A}$ of a finite ground set $\mathcal {V}$, a set function $g:2^{\mathcal {V}}\rightarrow \mathbb {R}$ is *submodular* if it satisfies the diminishing-returns property

$$ g(\mathcal {A} \cup \{e\}) - g(\mathcal {A}) \ge g(\mathcal {B} \cup \{e\}) - g(\mathcal {B}), \qquad \forall \mathcal {A} \subseteq \mathcal {B} \subseteq \mathcal {V},\ \forall e \in \mathcal {V} {\setminus } \mathcal {B}. $$

A function is *monotone non-decreasing* if $g(\mathcal {A})\le g(\mathcal {B})$ whenever $\mathcal {A} \subseteq \mathcal {B}$. Maximizing a monotone submodular function under a cardinality constraint is NP-hard, but the standard greedy algorithm attains a *(1-1/e)* approximation to the optimum^24^. SSFSS leverages this guarantee in both stages of the pipeline.

### Stage 1: Label-aware coreset greedy-based feature selection (LA-CGFS)

**Fig. 2:**
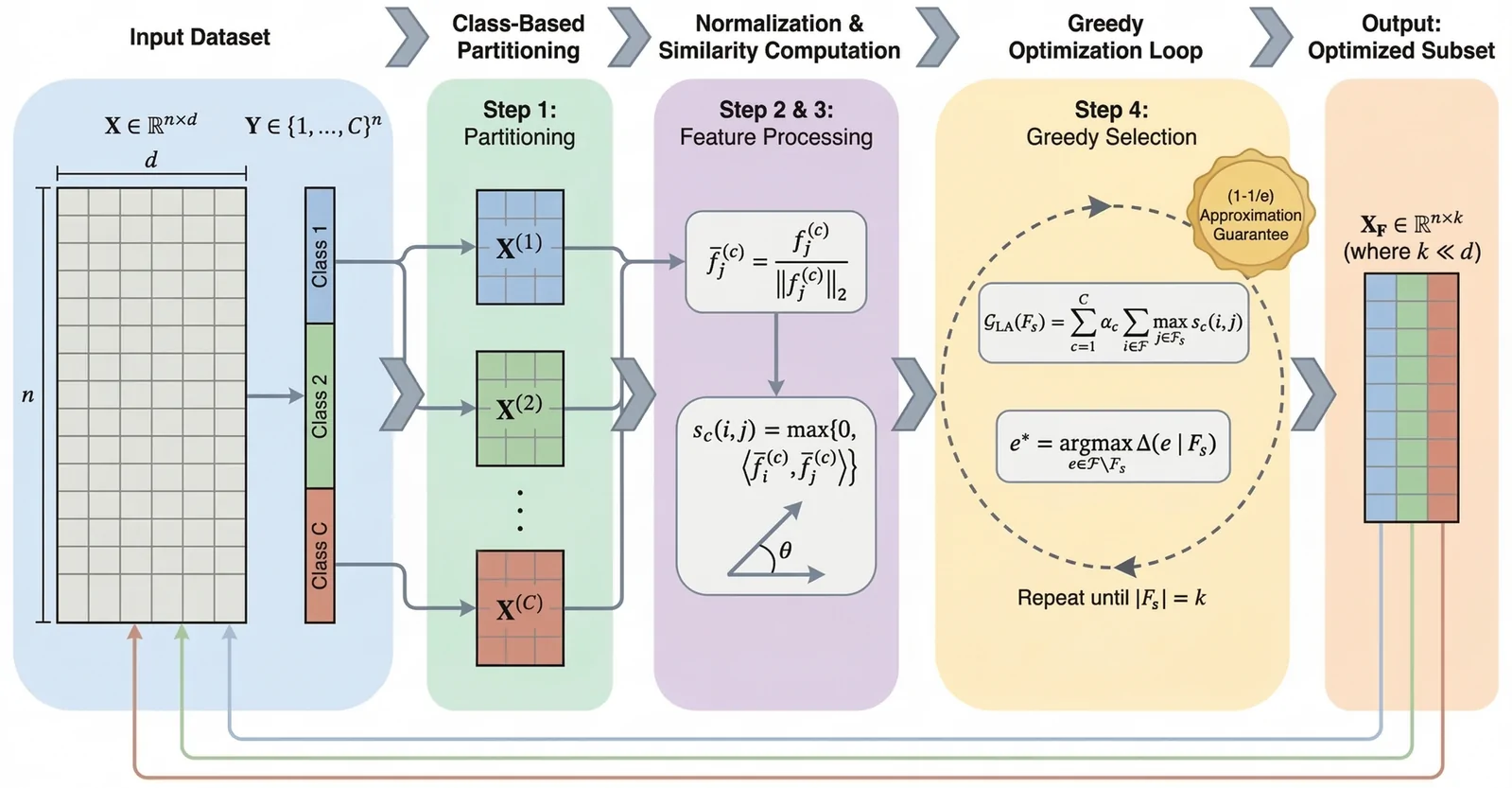
Flow of the proposed LA-CGFS stage. Class-conditioned feature views are formed first by partitioning $\boldsymbol{X} \in \mathbb {R}^{n \times d}$ into *C* class-restricted matrices $\boldsymbol{X}^{(c)}$ and $\ell _2$-normalizing their columns, after which greedy facility-location maximization of $\mathcal {G}_{\text {LA}}(F_s) = \sum _c \sum _i \max _{j \in F_s} s_c(i,j)$ iteratively selects the *k* most representative and discriminative features with a $\left( 1 - \frac{1}{e}\right)$ approximation guarantee, producing the reduced feature matrix $\boldsymbol{X}_{F_s} \in \mathbb {R}^{n \times k}$.

The purpose of Stage 1 is to identify a compact subset of features that preserves the class-discriminative structure of the training data. This is especially important in IoT intrusion detection because globally dominant dimensions are often shaped by majority-class traffic and may fail to retain minority attack signatures. LA-CGFS addresses this issue by evaluating feature coverage separately within each class and then aggregating those class-conditional coverages into a single submodular objective. Figure 2 illustrates the overall flow of the proposed label-aware feature-selection stage.

#### Label-conditioned facility-location objective

For each class $c \in \{1,\dots ,C\}$, define the class-specific sample index set

$$ \mathcal {I}_{c} = \{i \in \mathcal {I} \mid y_i = c\}, $$

and let $\boldsymbol{X}^{(c)} = \boldsymbol{X}[\mathcal {I}_c,:] \in \mathbb {R}^{|\mathcal {I}_c| \times d}$ denote the corresponding class-restricted data matrix. The class-conditional representation of feature *j* is then the *j*^th^ column of $\boldsymbol{X}^{(c)}$, denoted by $\boldsymbol{f}_j^{(c)} \in \mathbb {R}^{|\mathcal {I}_c|}$.

Because cosine similarity is more stable than raw Euclidean distance for comparing high-dimensional feature columns^33,34^, each class-restricted feature vector is first $\ell _2$-normalized:

$$ \bar{\boldsymbol{f}}_j^{(c)} = {\left\{ \begin{array}{ll} \dfrac{\boldsymbol{f}_j^{(c)}}{\Vert \boldsymbol{f}_j^{(c)}\Vert _2}, & \text {if } \Vert \boldsymbol{f}_j^{(c)}\Vert _2 > 0,\\ 0, & \text {otherwise}. \end{array}\right. } $$

We then define the nonnegative class-conditional similarity

$$ s_c(i,j) = \max \bigl \{0,\ \langle \bar{\boldsymbol{f}}_i^{(c)}, \bar{\boldsymbol{f}}_j^{(c)} \rangle \bigr \}, $$

where the rectification at zero ensures that only positive alignment contributes to coverage and preserves monotonicity of the resulting facility-location objective.

The label-aware feature coverage function is

1 $$\begin{aligned} \mathcal {G}_{\text {LA}}(F_s) = \sum _{c=1}^{C} \sum _{i \in \mathcal {F}} \max _{j \in F_s} s_c(i,j), \qquad \max _{j \in \emptyset } s_c(i,j) = 0. \end{aligned}$$

The inner maximization measures how well feature *i* is represented by the selected set within class *c*, while the outer sum enforces coverage across all classes equally. Since (1) is a nonnegative weighted sum of facility-location functions, $\mathcal {G}_{\text {LA}}$ is monotone and submodular.

The Stage 1 optimization problem is therefore

2 $$\begin{aligned} F_s^{\star } = \arg \max _{F_s \subseteq \mathcal {F},\, |F_s| \le k} \mathcal {G}_{\text {LA}}(F_s). \end{aligned}$$

By construction, maximizing $\mathcal {G}_{\text {LA}}$ promotes features that remain representative across both majority and minority class manifolds, rather than features that are merely globally dominant.

#### Greedy optimization for LA-CGFS

Define the current class-conditional coverage of feature *i* under a selected set $F_s$ as

$$ m_{c,i}(F_s) = \max _{j \in F_s} s_c(i,j), \qquad m_{c,i}(\emptyset )=0. $$

For a candidate feature $e \in \mathcal {F}{\setminus } F_s$, the marginal gain is

3 $$\begin{aligned} \begin{aligned} \Delta (e \mid F_s)&= \mathcal {G}_{\text {LA}}(F_s \cup \{e\}) - \mathcal {G}_{\text {LA}}(F_s) \\&= \sum _{c=1}^{C} \sum _{i \in \mathcal {F}} \max \bigl \{0,\ s_c(i,e) - m_{c,i}(F_s)\bigr \}. \end{aligned} \end{aligned}$$

LA-CGFS starts from the empty set and repeatedly adds the feature with the largest marginal gain until *k* features have been selected. Because the objective is monotone submodular, the exact greedy procedure inherits the classical *(1-1/e)* approximation guarantee^24^. The complete procedure is summarized in Algorithm 1. It is important to note that the *(1-1/e)* approximation guarantee applies strictly to the maximization of the surrogate feature coverage objective, $\mathcal {G}_{\text {LA}}$. While this guarantees a near-optimal preservation of the class-conditional feature geometries, it does not act as a direct theoretical bound on the downstream classification accuracy or F1-score. Rather, the submodular guarantee ensures that the reduced feature subspace retains maximum representational fidelity, which serves as a highly effective empirical prerequisite for strong end-to-end IDS performance.

**Algorithm 1:**
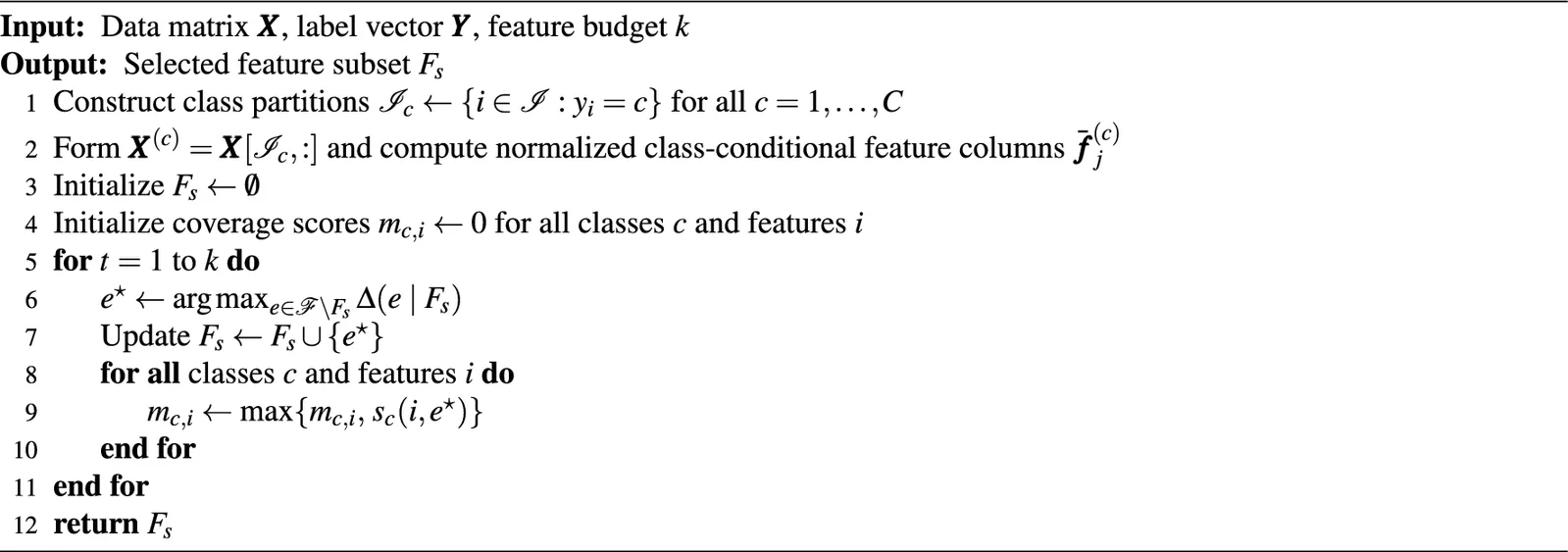
Label-Aware Coreset Greedy-Based Feature Selection (LA-CGFS)

#### Efficient implementation of LA-CGFS

To keep Stage 1 practical for large IDS datasets, we use the following implementation choices.

1. **Cosine similarity via normalization:** After $\ell _2$ normalization, cosine similarity reduces to a standard inner product, which allows the class-conditional similarities $s_c(i,j)$ to be evaluated efficiently using matrix multiplication or inner-product search.
2. **Similarity indexing with FAISS:** In large feature spaces, we can accelerate class-wise inner-product lookup by employing FAISS-based search over the normalized feature columns. This approach influences only the runtime and does not change the underlying optimization objective. The theoretical *(1-1/e)* guarantee discussed earlier applies to the exact greedy (or lazy-greedy) evaluation of marginal gains. In our experiments, we used the FAISS flat index to ensure 100% search accuracy, thus preserving the submodular approximation guarantee.
3. **Lazy-greedy evaluation:** Because $\mathcal {G}_{\text {LA}}$ is submodular, marginal gains exhibit diminishing returns. A lazy-greedy priority queue therefore avoids unnecessary recomputation of gains while preserving the exact greedy solution when gains are re-evaluated at extraction time.

### Stage 2: Geometry-aware sample space reduction

After Stage 1, each training sample is represented by the reduced vector $\boldsymbol{x}_i^{(F_s)} \in \mathbb {R}^{k}$, and the projected dataset is $\boldsymbol{X}_{F_s} \in \mathbb {R}^{n \times k}$. The goal of Stage 2 is to construct a weighted sample coreset $\mathcal {S} \subseteq \mathcal {I}$ with $|\mathcal {S}| \le m$ such that training on the coreset closely approximates training on the full projected dataset. This stage is the key mechanism by which SSFSS reduces the retraining cost of the downstream IDS model.

Let $\ell (\boldsymbol{\theta }; \boldsymbol{x}, y)$ denote the per-sample loss for model parameters $\boldsymbol{\theta }$. Define the full projected empirical gradient and the weighted coreset gradient, respectively, as

4 $$\begin{aligned} \boldsymbol{g}_{\text {full}}^{(F_s)}(\boldsymbol{\theta }) = \frac{1}{n}\sum _{i \in \mathcal {I}} \nabla _{\boldsymbol{\theta }}\ell (\boldsymbol{\theta }; \boldsymbol{x}_i^{(F_s)}, y_i), \end{aligned}$$

and

5 $$\begin{aligned} \boldsymbol{g}_{\mathcal {S},\boldsymbol{\gamma }}^{(F_s)}(\boldsymbol{\theta }) = \frac{1}{n}\sum _{j \in \mathcal {S}} \gamma _j \nabla _{\boldsymbol{\theta }}\ell (\boldsymbol{\theta }; \boldsymbol{x}_j^{(F_s)}, y_j). \end{aligned}$$

where $\gamma _j \ge 0$ in eq. (5) is the weight assigned to selected sample *j*, indicating how much that sample contributes to the coreset gradient; intuitively, $\gamma _j$ can be viewed as the number of projected training samples that sample *j* represents in the reduced dataset. The ideal Stage 2 objective is to choose $\mathcal {S}$ and nonnegative weights $\boldsymbol{\gamma }$ with $\sum _{j \in \mathcal {S}} \gamma _j = n$ so that these two gradients are as close as possible:

6 $$\begin{aligned} \min _{\mathcal {S},\boldsymbol{\gamma }} \left\| \boldsymbol{g}_{\text {full}}^{(F_s)}(\boldsymbol{\theta }) - \boldsymbol{g}_{\mathcal {S},\boldsymbol{\gamma }}^{(F_s)}(\boldsymbol{\theta }) \right\| _2 . \end{aligned}$$

Directly optimizing the gradient matching objective (6) is computationally expensive due to the need for repeated gradient calculations while selecting subsets. To address this challenge, we utilize a geometric surrogate inspired by coreset theory and related gradient-matching methods^20–22^. Under specific input-Lipschitz assumptions (which will be elaborated in the next section), ensuring adequate geometric coverage in the reduced feature space helps maintain control over the gradient approximation error. Consequently, geometric coreset selection provides a practical alternative to direct optimization in the full gradient space. Most importantly, this method allows for coreset selection to be treated as a preprocessing step, circumventing the computational difficulties associated with direct gradient-based optimization.

#### Distance-based sample coverage objective

A crucial theoretical requirement inherited from gradient-matching theory ^22^ is that the bound connecting feature-space distance to gradient discrepancy, i.e., $\Vert \nabla \ell (\boldsymbol{\theta }; \boldsymbol{x}_i) - \nabla \ell (\boldsymbol{\theta }; \boldsymbol{x}_j)\Vert \le L \Vert \boldsymbol{x}_i - \boldsymbol{x}_j\Vert$, is generally valid only when the two samples share the same label ($y_i = y_j$). When labels differ, gradients can point in opposite directions, and a small feature distance does not guarantee a small gradient difference. Therefore, we do *not* select the coreset globally across all classes. Instead, we apply the geometric coreset selection independently to each label class.

Let $\mathcal {I}_c = \{ i \mid y_i = c \}$ denote the indices of training samples belonging to class *c*, and let $n_c = |\mathcal {I}_c|$. For each class *c*, we select a class-specific coreset $\mathcal {S}_c \subseteq \mathcal {I}_c$ of size $m_c$ (where $\sum _c m_c = m$), along with class-specific weights $\boldsymbol{\gamma }^{(c)}$. The final sample coreset is the union $\mathcal {S} = \bigcup _c \mathcal {S}_c$. This per-class strategy maintains the class distribution of the full dataset, prevents majority-class bias, and ensures that the theoretical upper bound on the gradient estimation error formally applies to the combined selected set.

For a candidate per-class coreset $\mathcal {S}_c \subseteq \mathcal {I}_c$, define the residual distance of sample $i \in \mathcal {I}_c$ to the selected set as

$$ r_i(\mathcal {S}_c) = \min _{j \in \mathcal {S}_c} \left\| \boldsymbol{x}_i^{(F_s)} - \boldsymbol{x}_j^{(F_s)} \right\| _2. $$

**Fig. 3:**
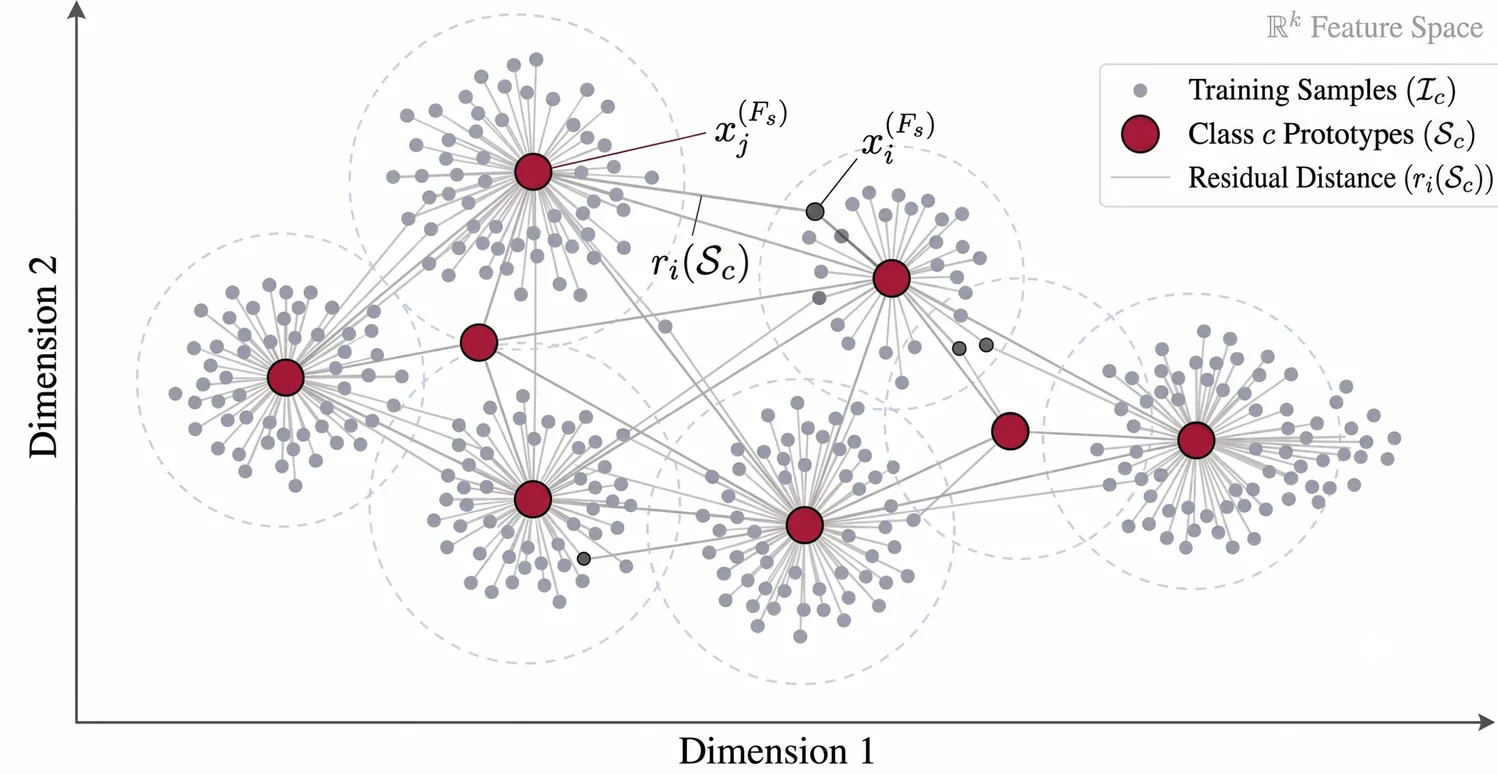
Visualization of the per-class distance-based sample-coverage objective in the reduced feature space $\mathbb {R}^k$. Grey dots are training samples $\boldsymbol{x}_i^{(F_s)}$ belonging to a specific class *c*; large filled circles are the $m_c$ selected prototypes $\boldsymbol{x}_j^{(F_s)} \in \mathcal {S}_c$ for that class. Each line segment has length $r_i(\mathcal {S}_c) = \min _{j \in \mathcal {S}_c} \Vert \boldsymbol{x}_i^{(F_s)} - \boldsymbol{x}_j^{(F_s)}\Vert _2$, the residual distance of sample *i* to its nearest prototype within the same class. Dashed circles delimit each prototype’s coverage neighborhood. The optimization objective $\min _{\mathcal {S}_c, |\mathcal {S}_c| \le m_c} \sum _{i \in \mathcal {I}_c} r_i(\mathcal {S}_c)$ is solved independently per class, and the final global coreset is obtained as the union $\mathcal {S} = \bigcup _c \mathcal {S}_c$.

The corresponding per-class sample-reduction problem is

7 $$\begin{aligned} \min _{\mathcal {S}_c \subseteq \mathcal {I}_c,\, |\mathcal {S}_c| \le m_c} \sum _{i \in \mathcal {I}_c} r_i(\mathcal {S}_c), \end{aligned}$$

which seeks a set of prototypes within the class that minimizes the aggregate representation error. Figure 3 visualizes this objective.

To express (7) as a monotone submodular maximization problem, let

$$ \kappa _c \ge \max _{p,q \in \mathcal {I}_c} \left\| \boldsymbol{x}_p^{(F_s)} - \boldsymbol{x}_q^{(F_s)} \right\| _2, $$

(which is simply the diameter of the class in the reduced space), and define the nonnegative similarity

$$ s_c(i,j) = \kappa _c - \Vert \boldsymbol{x}_i^{(F_s)} - \boldsymbol{x}_j^{(F_s)}\Vert _2. $$

Then minimizing the residual distance is equivalent, up to the constant $n_c \kappa _c$, to maximizing the per-class facility-location objective

8 $$\begin{aligned} \mathcal {H}_{\text {SCS}}^{(c)}(\mathcal {S}_c) = \sum _{i \in \mathcal {I}_c} \max _{j \in \mathcal {S}_c} s_c(i,j) = n_c \kappa _c - \sum _{i \in \mathcal {I}_c} r_i(\mathcal {S}_c). \end{aligned}$$

Because it is again a facility-location function with nonnegative similarities, $\mathcal {H}_{\text {SCS}}^{(c)}$ is monotone and submodular.

Define the current coverage of sample *i* under $\mathcal {S}_c$ as

$$ m_i(\mathcal {S}_c) = \max _{j \in \mathcal {S}_c} s_c(i,j). $$

For a candidate sample $e \in \mathcal {I}_c {\setminus } \mathcal {S}_c$, the marginal gain can be written as

$$ \Delta ^{(c)}(e \mid \mathcal {S}_c) = \sum _{i \in \mathcal {I}_c} \max \{0,\ s_c(i,e) - m_i(\mathcal {S}_c)\}, $$

or equivalently as

9 $$\begin{aligned} \Delta ^{(c)}(e \mid \mathcal {S}_c) = \sum _{i \in \mathcal {I}_c} \max \!\left\{ 0,\, r_i(\mathcal {S}_c) - \left\| \boldsymbol{x}_i^{(F_s)} - \boldsymbol{x}_e^{(F_s)}\right\| _2 \right\} . \end{aligned}$$

Thus, each selected prototype is the sample that most reduces the total residual distance within its class in the reduced feature space.

#### Greedy optimization and weight construction

Since $\mathcal {H}_{\text {SCS}}^{(c)}$ is monotone submodular, a greedy strategy yields a *(1-1/e)* approximation to the optimal per-class sample coreset of size $m_c$^22,24^. Starting from the empty set, the algorithm repeatedly adds the sample with the largest marginal gain and updates the residual distances of all points within that class. The complete procedure is summarized in Algorithm 2. The final global coreset is obtained as $\mathcal {S} = \bigcup _c \mathcal {S}_c$.

Similar to Stage 1, the *(1-1/e)* bound achieved by the greedy coreset selection applies exclusively to the submodular geometric coverage objective, $\mathcal {H}_{\text {SCS}}^{(c)}$. This mathematically guarantees that the selected prototypes minimize the aggregate residual representation error within the reduced space for each class. This geometric guarantee acts as a structural surrogate to minimize gradient discrepancy, but it must be distinguished from a formal generalization bound on the ultimate convergence rate or test accuracy of the downstream intrusion detection models.

**Algorithm 2:**
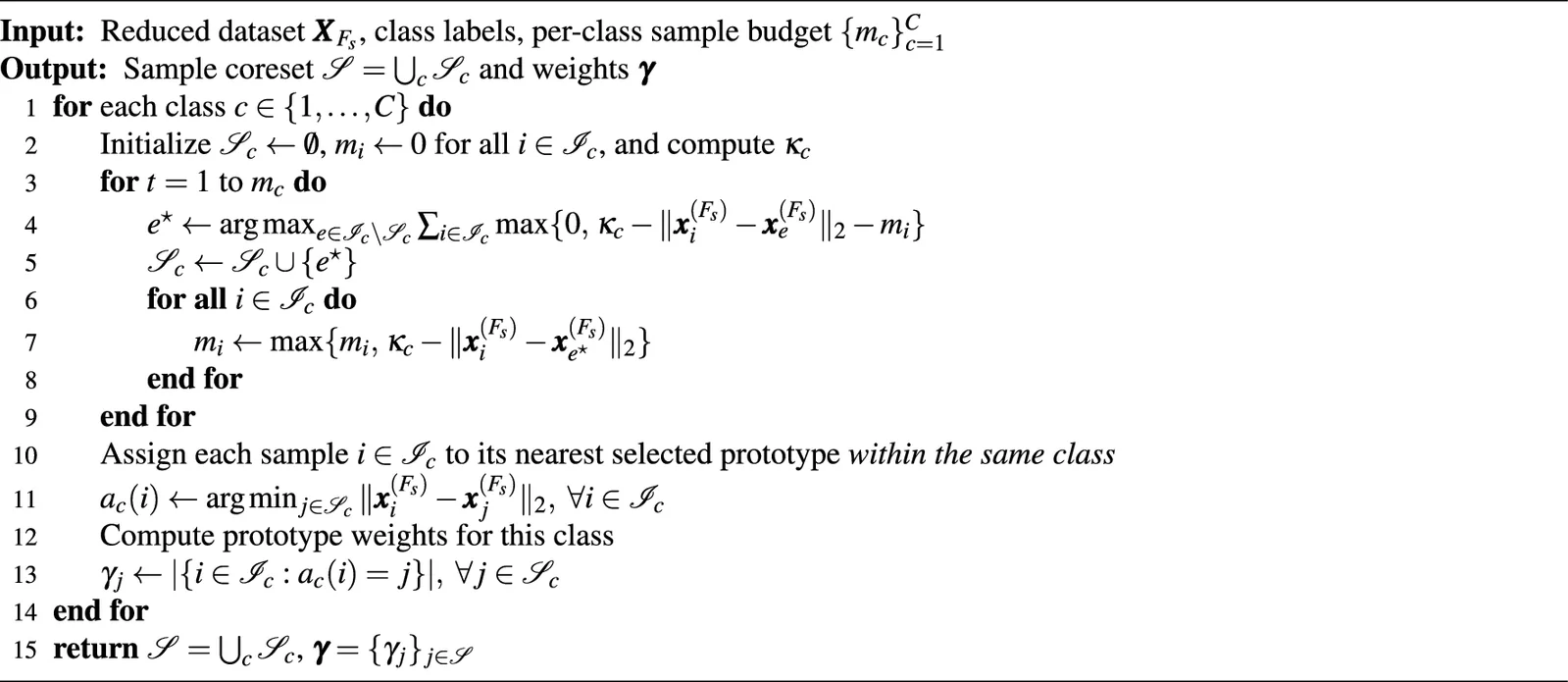
Sample Coreset Selection (SCS)—Per-Class Execution

Crucially, the weighted coreset reduces the training set size directly rather than synthetically expanding it, which is precisely the behavior required for practical edge-side learning and frequent retraining. Since the full gradient is the sum of class-wise gradients, and we approximate each class-wise gradient individually within a bounded feature space, the total approximation error is controlled by the sum of the per-class geometric coverage errors.

Please note that, the sequential order of SSFSS is essential. Selecting samples before eliminating redundant features would force the coreset objective to operate in a noisier and potentially majority-biased space, wasting sample budget on distinctions that disappear after feature reduction. By first constructing a label-aware compact representation and then performing sample coreset selection independently per class in that representation, SSFSS aligns the reduction process with the final training geometry used by the downstream IDS model.

## Error analysis

In this section, we quantify the discrepancy between training on the full dataset and training on the reduced dataset returned by the two sequential stages of SSFSS. The derivation is stated for differentiable empirical-risk minimizers. Since SSFSS is a model-agnostic preprocessing framework, the resulting bound should be interpreted as a formal optimization guarantee for differentiable learners and as a representation-level justification for the reduced training set used by non-differentiable classifiers. To keep the main text focused on the bound itself, only the assumptions, linking equations, and final result statements are given here; the complete proofs are deferred to Appendix 1.

### Problem setup and error decomposition

We now quantify the end-to-end approximation error introduced by the two sequential reductions in SSFSS. To compare the original training set with the Stage 1 and Stage 2 outputs in a common ambient space, let $\boldsymbol{P}_{F_s}:\mathbb {R}^{d}\rightarrow \mathbb {R}^{d}$ denote the coordinate projector that keeps the entries indexed by $F_s$ and sets all remaining coordinates to zero. For the purpose of the error analysis, we use the same notation

$$ \boldsymbol{x}_i^{(F_s)} \triangleq \boldsymbol{P}_{F_s}\boldsymbol{x}_i \in \mathbb {R}^{d}, $$

that is, $\boldsymbol{x}_i^{(F_s)}$ is interpreted here as the zero-padded embedding of the reduced representation introduced in Stage 1.

We first define the empirical gradient over the full, unreduced training set:

10 $$\begin{aligned} \boldsymbol{g}_{\text {full}}(\boldsymbol{\theta }) = \frac{1}{n}\sum _{i=1}^{n} \nabla _{\boldsymbol{\theta }}\ell (\boldsymbol{\theta }; \boldsymbol{x}_i,y_i). \end{aligned}$$

The corresponding projected full-data gradient $\boldsymbol{g}_{\text {full}}^{(F_s)}(\boldsymbol{\theta })$ and weighted coreset gradient $\boldsymbol{g}_{\mathcal {S},\boldsymbol{\gamma }}^{(F_s)}(\boldsymbol{\theta })$ have already been defined in Stage 2 by (4) and (5), respectively. We measure the overall approximation error between training on the original dataset and training on the final weighted coreset by

11 $$\begin{aligned} \mathcal {E}_{\text {total}} \triangleq \sup _{\boldsymbol{\theta } \in \Theta } \left\| \boldsymbol{g}_{\text {full}}(\boldsymbol{\theta }) - \boldsymbol{g}_{\mathcal {S},\boldsymbol{\gamma }}^{(F_s)}(\boldsymbol{\theta }) \right\| _2. \end{aligned}$$

To relate perturbations in the input representation to the corresponding change in the training gradient, we impose a *label-conditional* Lipschitz regularity condition on the per-sample loss gradient. Specifically, we assume that there exists a constant $L_{\text {in}}\ge 0$ such that, for all $\boldsymbol{\theta } \in \Theta$, all $\boldsymbol{u},\boldsymbol{v} \in \mathbb {R}^{d}$, and every fixed label $y \in \{1,\dots ,C\}$,

12 $$\begin{aligned} \left\| \nabla _{\boldsymbol{\theta }}\ell (\boldsymbol{\theta };\boldsymbol{u},y) - \nabla _{\boldsymbol{\theta }}\ell (\boldsymbol{\theta };\boldsymbol{v},y) \right\| _2 \le L_{\text {in}}\Vert \boldsymbol{u}-\boldsymbol{v}\Vert _2 . \end{aligned}$$

This assumption states that, *within a given class*, the gradient varies smoothly with respect to changes in the input vector. It is deliberately weaker than a joint input–label smoothness condition: because both stages of SSFSS compare a sample only with a surrogate carrying the *same* label, no cross-label gradient comparison ever arises in the analysis. Concretely, Stage 1 replaces $(\boldsymbol{x}_i,y_i)$ by $(\boldsymbol{x}_i^{(F_s)},y_i)$, which leaves the label untouched, and Stage 2 replaces each projected sample by a prototype drawn from its own class, since the coverage objective (8) is maximized independently within every class *c*. Consequently, (12) is the only regularity condition needed for the whole pipeline, and no label-mismatch term appears in the resulting bounds.

The total approximation error in (11) can then be separated into the two errors induced by the sequential stages of SSFSS. By adding and subtracting the projected full-data gradient $\boldsymbol{g}_{\text {full}}^{(F_s)}(\boldsymbol{\theta })$, defined in (4), and applying the triangle inequality uniformly over *Θ *, we obtain

13 $$\begin{aligned} \mathcal {E}_{\text {total}} \le \underbrace{ \sup _{\boldsymbol{\theta } \in \Theta } \left\| \boldsymbol{g}_{\text {full}}(\boldsymbol{\theta }) - \boldsymbol{g}_{\text {full}}^{(F_s)}(\boldsymbol{\theta }) \right\| _2 }_{\Delta _{\text {feat}}(F_s)} + \underbrace{ \sup _{\boldsymbol{\theta } \in \Theta } \left\| \boldsymbol{g}_{\text {full}}^{(F_s)}(\boldsymbol{\theta }) - \boldsymbol{g}_{\mathcal {S},\boldsymbol{\gamma }}^{(F_s)}(\boldsymbol{\theta }) \right\| _2 }_{\Delta _{\text {samp}}(\mathcal {S},\boldsymbol{\gamma }\mid F_s)}. \end{aligned}$$

The first term, $\Delta _{\text {feat}}(F_s)$, captures the approximation error introduced by Stage 1 feature reduction alone. The second term, $\Delta _{\text {samp}}(\mathcal {S},\boldsymbol{\gamma }\mid F_s)$, captures the additional error introduced by Stage 2 sample compression after the data have already been projected onto the selected feature subset. This decomposition follows the sequential design of SSFSS and cleanly separates the contribution of each reduction stage to the final training error.

### Stage 1: Feature-stage error bound

The first term in the decomposition (13), namely $\Delta _{\text {feat}}(F_s)$, measures the error introduced when the original samples are replaced by their Stage 1 projected representations. To quantify this effect, we define the average projection distortion of the selected feature subset $F_s$ by

14 $$\begin{aligned} \varepsilon _{\text {feat}}(F_s) = \frac{1}{n}\sum _{i=1}^{n} \left\| \boldsymbol{x}_i-\boldsymbol{x}_i^{(F_s)} \right\| _2. \end{aligned}$$

This quantity summarizes how much information is lost, on average, when each sample is mapped from the original feature space to the Stage 1 representation.

#### Theorem 4.1

Under (12), the Stage 1 error term satisfies

15 $$\begin{aligned} \Delta _{\text {feat}}(F_s) \le L_{\text {in}}\,\varepsilon _{\text {feat}}(F_s). \end{aligned}$$

Theorem 4.1 shows that the contribution of Stage 1 is controlled entirely by the average projection distortion. Hence, a feature subset that preserves the original samples well in Euclidean norm also preserves the corresponding empirical training gradient. The proof is given in Appendix A.1.

### Stage 2: Sample-stage error bound

We now bound the second term in (13), namely $\Delta _{\text {samp}}(\mathcal {S},\boldsymbol{\gamma }\mid F_s)$, which measures the additional error introduced by compressing the Stage 1 projected dataset into a weighted coreset. Throughout this subsection, the feature subset $F_s$ is fixed, and we compare the projected full-data gradient (4) with the weighted coreset gradient (5).

Recall from Stage 2 that the coreset is built independently within each class: for every $c \in \{1,\dots ,C\}$ a subset $\mathcal {S}_c \subseteq \mathcal {I}_c$ with $|\mathcal {S}_c| \le m_c$ is selected, and the final coreset is $\mathcal {S} = \bigcup _{c=1}^{C}\mathcal {S}_c$. Accordingly, each sample is represented by a prototype of its own class. For each class *c* and each sample $i \in \mathcal {I}_c$, let

16 $$\begin{aligned} a_c(i)\in \arg \min _{j \in \mathcal {S}_c} \left\| \boldsymbol{x}_i^{(F_s)}-\boldsymbol{x}_j^{(F_s)} \right\| _2 \end{aligned}$$

denote the nearest selected prototype within class *c* in the reduced feature space, so that $y_{a_c(i)}=y_i=c$ by construction. Since the Stage 2 weights are constructed as within-class Voronoi counts,

$$ \gamma _j = \bigl |\{i \in \mathcal {I}_c: a_c(i)=j\}\bigr |, \qquad j \in \mathcal {S}_c, $$

they satisfy $\sum _{j \in \mathcal {S}_c}\gamma _j = n_c$ for every class and therefore $\sum _{j \in \mathcal {S}}\gamma _j = \sum _{c=1}^{C} n_c = n$. The weighted coreset gradient in (5) can then be rewritten as

17 $$\begin{aligned} \boldsymbol{g}_{\mathcal {S},\boldsymbol{\gamma }}^{(F_s)}(\boldsymbol{\theta }) = \frac{1}{n}\sum _{c=1}^{C}\sum _{i \in \mathcal {I}_c} \nabla _{\boldsymbol{\theta }}\ell \!\left( \boldsymbol{\theta }; \boldsymbol{x}_{a_c(i)}^{(F_s)}, y_i\right) . \end{aligned}$$

This representation makes the Stage 2 approximation explicit: each original projected sample is replaced by the prototype (with the same label) that represents it in the coreset. To quantify this approximation, we define the mean sample-coverage error of class *c* by

18 $$\begin{aligned} \varepsilon _{\text {samp}}^{(c)}(\mathcal {S}_c\mid F_s) = \frac{1}{n_c}\sum _{i \in \mathcal {I}_c} \left\| \boldsymbol{x}_i^{(F_s)}-\boldsymbol{x}_{a_c(i)}^{(F_s)} \right\| _2 = \frac{1}{n_c}\sum _{i \in \mathcal {I}_c} r_i(\mathcal {S}_c), \end{aligned}$$

and the aggregate sample-coverage error as its support-weighted average over the classes,

19 $$\begin{aligned} \varepsilon _{\text {samp}}(\mathcal {S}\mid F_s) = \frac{1}{n}\sum _{c=1}^{C}\sum _{i \in \mathcal {I}_c} \left\| \boldsymbol{x}_i^{(F_s)}-\boldsymbol{x}_{a_c(i)}^{(F_s)} \right\| _2 = \sum _{c=1}^{C}\frac{n_c}{n}\, \varepsilon _{\text {samp}}^{(c)}(\mathcal {S}_c\mid F_s). \end{aligned}$$

Here, $\varepsilon _{\text {samp}}^{(c)}(\mathcal {S}_c\mid F_s)$ is exactly the average residual distance induced by the per-class Stage 2 coverage objective (8); equivalently,

20 $$\begin{aligned} \varepsilon _{\text {samp}}^{(c)}(\mathcal {S}_c\mid F_s) = \kappa _c - \frac{1}{n_c}\,\mathcal {H}_{\text {SCS}}^{(c)}(\mathcal {S}_c), \end{aligned}$$

so that maximizing the per-class facility-location objective is precisely equivalent to minimizing the per-class coverage error that enters the bound. In other words, the geometric error is the only source of Stage 2 discrepancy.

#### Theorem 4.2

Under (12), and with the per-class assignment (16), the Stage 2 error term satisfies

21 $$\begin{aligned} \Delta _{\text {samp}}(\mathcal {S},\boldsymbol{\gamma }\mid F_s) \le L_{\text {in}}\,\varepsilon _{\text {samp}}(\mathcal {S}\mid F_s) = L_{\text {in}} \sum _{c=1}^{C}\frac{n_c}{n}\, \varepsilon _{\text {samp}}^{(c)}(\mathcal {S}_c\mid F_s). \end{aligned}$$

Theorem 4.2 shows that Stage 2 is governed by a single interpretable quantity: geometric coverage in the reduced feature space, measured separately inside each class and then aggregated in proportion to the class supports. A further consequence of (21) is that every class, including heavily under-represented attack classes, contributes its own coverage term. The proof is given in Appendix A.2.

### Unified bound for the sequential pipeline

We can now combine the Stage 1 and Stage 2 bounds through the decomposition in (13). This yields a single end-to-end guarantee for the sequential SSFSS pipeline.

#### Theorem 4.3

Let $F_s$ be the feature subset returned by Stage 1, and let $(\mathcal {S},\boldsymbol{\gamma })$ with $\mathcal {S}=\bigcup _{c=1}^{C}\mathcal {S}_c$ be the per-class weighted coreset returned by Stage 2 on the projected representation induced by $F_s$. Under (12),

22 $$\begin{aligned} \mathcal {E}_{\text {total}} \le L_{\text {in}} \left[ \varepsilon _{\text {feat}}(F_s) + \sum _{c=1}^{C}\frac{n_c}{n}\, \varepsilon _{\text {samp}}^{(c)}(\mathcal {S}_c\mid F_s) \right] . \end{aligned}$$

Theorem 4.3 directly mirrors the two-stage structure of SSFSS. The first term captures the approximation error caused by feature reduction, while the second term captures the geometric loss caused by sample compression after projection, expressed as the support-weighted sum of the residual coverage errors of the individual class coresets. No third term is needed: because Stage 2 assigns every sample to a prototype of its own class, the prototype assignment is label-consistent by construction, and the discrepancy that would otherwise arise from cross-label substitution is eliminated at the design level. Therefore, the sequential pipeline remains accurate whenever Stage 1 preserves the original samples well and Stage 2 provides good coverage inside each class in the reduced feature space. The proof is given in Appendix A.3.

Furthermore, the bounds derived in this section quantify the empirical gradient approximation error ($\mathcal {E}_{\text {total}}$) induced by the sequential feature-sample reduction. They provide a rigorous mathematical justification for why the reduced coreset serves as a faithful optimization surrogate for the full dataset. However, these bounds evaluate empirical training-set fidelity rather than test-set generalization. Therefore, the theoretical guarantees provided by the SSFSS framework constrain the data-reduction approximation error, whereas the end-to-end classification quality and generalization to unseen IoT attacks remain governed by the inductive biases and capacity of the chosen downstream classifier.

## Experimental evaluation

This section assesses whether the SSFSS framework meets the objectives specified in Sections “The proposed sequential submodular feature-sample selection (SSFSS) framework” and “Error analysis”. Specifically, it focuses on maintaining the fidelity of multiclass intrusion detection while significantly reducing the time and memory required for repeated training. The theoretical guarantees discussed in these sections pertain to the family of *differentiable empirical-risk minimizers (ERMs)*, which is why our evaluation centers on this classifier family. This approach ensures that both the empirical study and the theoretical framework address the same hypothesis class. We examine both predictive performance and resource efficiency, emphasizing multiclass attack detection. Additionally, we highlight improvements in training duration, end-to-end timing speedups, and peak RAM reduction achieved by the proposed SSFSS framework on three benchmark IoT datasets.

### Experimental setup

#### Benchmark datasets

To evaluate the robustness of SSFSS across heterogeneous IoT settings, we use three widely adopted benchmarks: RT-IoT2022^2^, Edge-IIoTset^4^, and CICIoT2023^5^.

- **RT-IoT2022:** RT-IoT2022 contains 123,117 flows distributed over 12 traffic classes (9 attack classes and 3 benign device behaviors). It combines realistic IoT telemetry with attack traffic such as brute-force SSH, Slowloris, and Nmap-based probing, making it especially useful for examining minority-class behavior.
- **Edge-IIoTset:** The Edge-IIoTset subset used in this study contains 157,800 flows across 15 traffic classes (14 attack classes and 1 normal class). It covers diverse attacks such as DDoS, ransomware, SQL injection, XSS, and scanning activity in a heterogeneous edge/IIoT environment.
- **CICIoT2023:** Because the original CICIoT2023 corpus is very large, we use a class-stratified subset of 129,972 flows whose class counts are listed in Table 4. The subset is drawn by stratified sampling so that the native class proportions–and hence the heavy imbalance and attack diversity of the benchmark–are preserved, while keeping cross-method comparisons computationally tractable. To account for sampling variability, the subset is redrawn independently for each random seed, so that the reported mean and standard deviation also reflect the sensitivity of SSFSS to the particular subset realization.

The traffic distributions used in the experiments are reported in Tables 2, 3 and 4.

#### Data preprocessing

All datasets are first partitioned into 70% training data and 30% testing data using stratified sampling so that class proportions are preserved in both splits. Categorical attributes are one-hot encoded and continuous attributes are *z*-score normalized, with all statistics *fit on the training split only* and applied to the held-out test split to avoid information leakage. The standardized continuous features are computed as

23 $$\begin{aligned} z = \frac{x - \mu _{\text {train}}}{\sigma _{\text {train}}}, \end{aligned}$$

where $\mu _{\text {train}}$ and $\sigma _{\text {train}}$ denote the training-set mean and standard deviation of the feature. After preprocessing, Stage 1 (LA-CGFS) and Stage 2 (sample coreset selection) are learned exclusively from the training partition.

Because the differentiable ERM classifiers operate on a kernel representation, a unified feature map is applied to every classifier and every baseline so that predictive differences are attributable to the loss function rather than to model-specific preprocessing. Concretely, each classifier is fitted on the same pipeline: a per-feature quantile transformation to a standard normal marginal (which stabilizes the heavy-tailed, mixed categorical–continuous IoT features for distance-based kernels), followed by a Radial Basis Function (RBF) *Nyström* feature map that approximates the exact kernel with a fixed number of landmarks *D*. This keeps training linear in the number of samples–so the map is applicable to the full-data baseline as well as to the reduced coreset–while retaining the non-linear expressiveness of the kernel. The Nyström bandwidth is parameterized as *γ = c/d*, where *d* is the input dimension and *c* a per-dataset scale, so that the same map remains well-conditioned across both the full and the feature-reduced representations. The transformation is fit on the training partition only (or on the weighted coreset when the classifier is trained on the coreset), preserving the leakage-free protocol above.

#### Implementation environment

The framework and all baselines were implemented in Python 3.12. Classifiers were trained with scikit-learn, while FAISS was used to accelerate inner-product similarity search in LA-CGFS. All experiments were executed on a workstation with an Intel^®^ Core™ i7-10750H CPU @ 2.60 GHz and 32 GB RAM. Results are reported as the mean and standard deviation over three independent random seeds.

#### Classifiers and hyperparameters

In line with the theoretical framework of SSFSS, we evaluate four differentiable empirical-risk minimizers that share a unified RBF Nyström representation, as described in Section “Experimental setup”. These minimizers differ only in their loss functions: Kernel Logistic Regression (KLR, which uses a one-vs-rest logistic loss), Kernel Support Vector Machine with squared-hinge loss (KSVM, which is continuously differentiable), Kernel Ridge/least-squares classification (KRR, which employs squared loss), and Softmax/multinomial Logistic Regression (SoftmaxLR, which utilizes multinomial cross-entropy). Each of these methods is a differentiable empirical risk minimization (ERM) over a common fixed feature map, so the joint bound outlined in Section “Error analysis” applies uniformly to all four. For each dataset, we selected the shared bandwidth scale *c* and the regularization strength for each classifier based on a held-out validation split of a stratified subsample. These values were then fixed across all baselines and coreset budgets, ensuring that any observed differences stem from the data-reduction pipeline rather than from retuning each baseline. The selected parameter values are summarized in Table 1, and the number of Nyström landmarks is set at *D=500* for all datasets.

**Table 1:** Per-dataset hyperparameters for the differentiable ERM classifiers: shared RBF Nyström bandwidth scale *c* (with *γ =c/d*) and the regularization constant of each classifier (*C* for KLR, KSVM, and SoftmaxLR; ridge penalty *α * for KRR).

Dataset	*c*	KLR (*C*)	KSVM (*C*)	KRR (*α *) **/SoftmaxLR **(*C*)
RT-IoT2022	1.0	1000	100	0.01/1000
Edge-IIoTset	1.5	1000	100	0.01/1000
CICIoT2023	0.5	1000	100	0.01/1000

#### Baselines and comparison variants

We compare SSFSS against two reference baselines:

1. **Full Dataset:** training on the original feature space with all training samples.
2. **LA-CGFS (Stage 1):** training on the 20 features returned by Stage 1 while keeping 100% of the training samples.

The full SSFSS pipeline is then evaluated with coreset budgets of 5%, 10%, and 15% of the training split, denoted by **SSFSS-5**, **SSFSS-10**, and **SSFSS-15**, respectively. The percentage refers to the fraction of training samples retained after Stage 1 feature reduction. The Stage 1 feature budget is fixed at 20 features for all datasets; this choice, and its effect on the accuracy–efficiency trade-off, is justified in Section “LA-CGFS feature budget”.

#### Evaluation metrics

In the multiclass experiments, we present support-weighted Accuracy, Precision, Recall, F1-score, macro-F1 score, and balanced accuracy. All scores are calculated on the held-out test set and presented as the mean ± standard deviation across three independent seeds. Let *C* represent the number of classes and *N* denote the number of test instances, with $n_c$ indicating the instances belonging to class *c*, such that $N=\sum _{c=1}^{C} n_c$. In a one-vs-rest approach for each class, let $\textit{TP}_c$, $\textit{FP}_c$, and $\textit{FN}_c$ represent the true positives, false positives, and false negatives, respectively. The class-specific scores are

24 $$\begin{aligned} \text {Prec}_c = \frac{\textit{TP}_c}{\textit{TP}_c + \textit{FP}_c}, \qquad \text {Rec}_c = \frac{\textit{TP}_c}{\textit{TP}_c + \textit{FN}_c}, \end{aligned}$$

25 $$\begin{aligned} \text {F1}_c = \frac{2\,\text {Prec}_c\,\text {Rec}_c}{\text {Prec}_c + \text {Rec}_c}, \end{aligned}$$

The support-weighted metrics average the class-specific scores using the class frequencies $n_c/N$:

26 $$\begin{aligned} \text {Prec}_{\text {w}} = \sum _{c=1}^{C}\frac{n_c}{N}\,\text {Prec}_c, \quad \text {Rec}_{\text {w}} = \sum _{c=1}^{C}\frac{n_c}{N}\,\text {Rec}_c, \quad \text {F1}_{\text {w}} = \sum _{c=1}^{C}\frac{n_c}{N}\,\text {F1}_c . \end{aligned}$$

Accuracy is the overall fraction of correctly classified instances,

27 $$\begin{aligned} \text {Acc} = \frac{1}{N}\sum _{i=1}^{N}\textbf{1}\!\left[ \hat{y}_i = y_i\right] = \frac{1}{N}\sum _{c=1}^{C} \textit{TP}_c . \end{aligned}$$

Because the task is single-label multiclass classification, weighted Recall is numerically equal to Accuracy ($\text {Rec}_{\text {w}} = \text {Acc}$); this explains the identical values appearing in several rows of Tables 5, 9 and 13. To complement these frequency-weighted quantities, which are dominated by the majority classes, we additionally report two unweighted (macro) metrics that assign every class equal weight and are therefore more sensitive to minority-class behavior, namely the macro-F1 score and the balanced accuracy:

28 $$\begin{aligned} \text {F1}_{\text {macro}} = \frac{1}{C}\sum _{c=1}^{C}\text {F1}_c, \qquad \text {BalAcc} = \frac{1}{C}\sum _{c=1}^{C}\text {Rec}_c . \end{aligned}$$

Balanced accuracy is the macro-average of the per-class recall, which is the equal-weight mean of $\text {Rec}_c$, as opposed to the support-weighted $\text {Rec}_{\text {w}}$. To assess how well the Stage-2 coreset maintains the class structure of the training data, we also provide per-class *retention ratios*. Consider a full training split containing *M* samples, of which $m_c$ belong to class *c*, and a selected coreset $\mathcal {S}$ that includes $s_c$ samples of class *c*. Each coreset point *j* is assigned a Voronoi weight $w_j$, representing the number of training points for which the nearest coreset point is *j*. These weights collectively add up to the training-set size *M*, and the total weight associated with class *c* is denoted as $W_c$. The raw and weighted retention ratios for class *c* are

29 $$\begin{aligned} \rho _c^{\text {raw}} = \frac{s_c/|\mathcal {S}|}{m_c/M}, \qquad \rho _c^{\text {w}} = \frac{W_c/M}{m_c/M} = \frac{W_c}{m_c}, \qquad W_c = \!\!\sum _{j\in \mathcal {S}:\, y_j=c}\!\! w_j . \end{aligned}$$

A ratio of 1 indicates that class *c* occupies the same proportion in the coreset as it does in the full training split. Values above or below 1 signify over-representation or under-representation, respectively, while a value of 0 indicates that a class has been entirely dropped. The raw ratio $\rho _c^{\text {raw}}$ counts the retained samples and can diverge significantly from 1 because geometry-aware coreset selection compresses dense classes more aggressively than sparse ones. Conversely, the weighted ratio $\rho _c^{\text {w}}$ measures the effective class mass after applying Voronoi weighting. This ratio reflects the distribution presented to a classifier trained on the weighted coreset and remains 1 for each class. This consistency confirms that the weighting process restores the original class proportions and prevents minority attack classes from being neglected. Both ratios are reported per class as the mean ± standard deviation over three seeds.

The per-class tables report the mean classwise F1-score and Recall aggregated over the four differentiable ERM classifiers for each baseline. Resource efficiency is measured through *training time* and *peak RAM usage*. These metrics are reported for both coreset-based classifier fitting alone, which reflects the recurring per-update cost, and for the entire SSFSS pipeline. The latter includes Stage 1 (feature selection), Stage 2 (coreset selection and weighting), and coreset training. It is important to note that inference latency is out of scope for this section.

**Table 2:** RT-IoT2022 dataset traffic distribution.

Label	Traffic type	Number of samples
Attack	DOS_SYN_Hping	94,659
	ARP_poisoning	7750
	NMAP_UDP_SCAN	2590
	NMAP_XMAS_TREE_SCAN	2010
	NMAP_OS_DETECTION	2000
	NMAP_TCP_SCAN	1002
	DDOS_Slowloris	534
	Metasploit_Brute_Force_SSH	37
	NMAP_FIN_SCAN	28
Normal	MQTT	8108
	ThingSpeak	4146
	Wipro_Bulb_Dataset	253

**Table 3:** Edge-IIoTset dataset traffic distribution.

Label	Traffic type	Number of samples
Attack	DDoS_UDP	14,498
	DDoS_ICMP	14,090
	Ransomware	10,925
	DDoS_HTTP	10,561
	SQL_injection	10,311
	Uploading	10,269
	DDoS_TCP	10,247
	Backdoor	10,195
	Vulnerability_scanner	10,076
	Port_Scanning	10,071
	XSS	10,052
	Password	9989
	MITM	1214
	Fingerprinting	1001
Normal	Normal	24,301

**Table 4:** CICIoT2023 sampled subset traffic distribution.

Label	Traffic type	Number of samples
Attack	DDoS-ICMP_Flood	20,160
	DDoS-UDP_Flood	15,151
	DDoS-TCP_Flood	12,662
	DDoS-PSHACK_Flood	11,549
	DDoS-SYN_Flood	11,456
	DDoS-RSTFINFlood	11,353
	DDoS-SynonymousIP_Flood	10,085
	DoS-UDP_Flood	9348
	DoS-TCP_Flood	7476
	DoS-SYN_Flood	5651
	Mirai-greeth_flood	2764
	Mirai-udpplain	2521
	Mirai-greip_flood	2119
	DDoS-ICMP_Fragmentation	1278
	MITM-ArpSpoofing	878
	DDoS-UDP_Fragmentation	804
	DDoS-ACK_Fragmentation	804
	DNS_Spoofing	504
	DoS-HTTP_Flood	210
	DDoS-HTTP_Flood	78
	DDoS-SlowLoris	62
Normal	BenignTraffic	3059

### LA-CGFS feature budget

The two reference baselines and the SSFSS variants of Section “Experimental setup” fix the Stage 1 feature budget at *k=20* for all three datasets. We now justify this single, unified choice so that the results reported in the remainder of this section are not contingent on per-dataset feature-count tuning. The argument rests directly on the submodularity of the label-conditioned facility-location objective $\mathcal {G}_{\text {LA}}$ optimized by LA-CGFS (Section “The proposed sequential submodular feature-sample selection (SSFSS) framework”): because $\mathcal {G}_{\text {LA}}$ is monotone and submodular and the lazy-greedy selection adds features in non-increasing order of marginal gain, the coverage curve is concave and its increments diminish. The operating point *k* should therefore be placed where the marginal gain has effectively vanished–capturing almost all of the attainable coverage while discarding features that contribute negligibly to class separability.

**Fig. 4:**
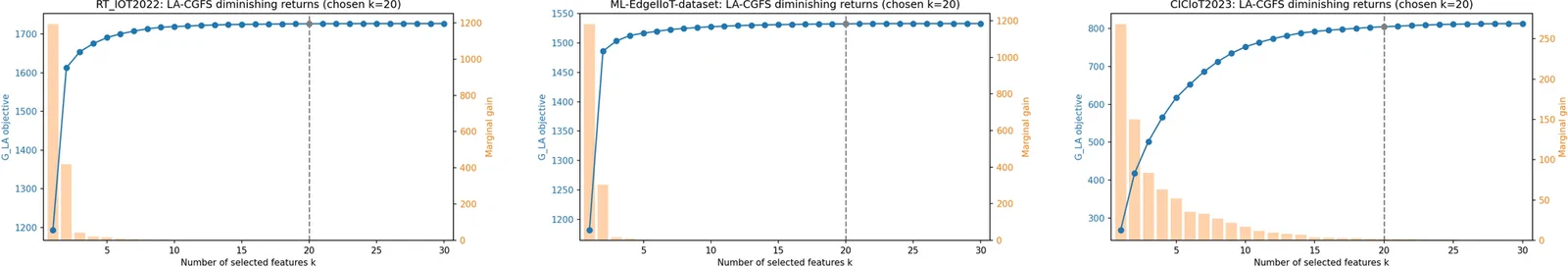
LA-CGFS Stage-1 diminishing returns on RT-IoT2022 (left), Edge-IIoTset (center), and CICIoT2023 (right). The cumulative class-weighted facility-location objective $G_{\text {LA}}$ (blue, left axis) rises steeply and then saturates, while the per-feature marginal gain (orange bars, right axis) collapses after the first few features. The dashed line marks the unified operating point *k=20*, at which the objective has reached *99.97%*, *99.97%*, and *98.97%* of its value at *k=30* on RT-IoT2022, Edge-IIoTset, and CICIoT2023, respectively.

Figure 4 reports the LA-CGFS diminishing-returns curves for the three benchmarks. For each dataset, the blue curve (left axis) is the cumulative $\mathcal {G}_{\text {LA}}$ objective as a function of the number of selected features *k*, and the orange bars (right axis) are the marginal gain contributed by each newly added feature. Since $\mathcal {G}_{\text {LA}}(\emptyset )=0$, the cumulative objective equals the running sum of the marginal gains. The curves are computed to *k=30* so that the residual coverage beyond the chosen operating point is visible, and the dashed vertical line marks *k=20*.

The three curves exhibit the same qualitative behavior predicted by submodularity: a sharp initial rise followed by a pronounced plateau. Quantitatively, at *k=20*, the objective attains *99.97%* of $\mathcal {G}_{\text {LA}}(k{=}30)$ on RT-IoT2022 and Edge-IIoTset and *98.97%* on CIC-IoT2023. The marginal contribution of the 20th feature is likewise negligible–below *0.03%* of the first feature’s gain on RT-IoT2022 and Edge-IIoTset, and *0.65%* on CICIoT2023–confirming that the remaining unselected features are almost entirely redundant with respect to the class-weighted coverage. The three datasets differ in how quickly they saturate: RT-IoT2022 and Edge-IIoTset reach near-complete coverage within roughly the first ten features, whereas CICIoT2023, with its larger feature set (48 attributes) and 22-way class structure, approaches the plateau more gradually and therefore represents the most demanding case; even there, 20 features suffice to recover essentially all of the objective. A smaller budget (e.g., *k=10*) would sacrifice a non-trivial fraction of coverage on CICIoT2023, while a larger budget yields no measurable gain on any dataset.

We therefore adopt *k=20* as a single, dataset-agnostic Stage-1 configuration throughout the paper. This choice is deliberately *not* tuned to downstream classifier accuracy: it is determined solely by the intrinsic saturation of the LA-CGFS objective, which keeps Stage 1 unsupervised of the evaluation metric and avoids information leakage from the test protocol. Fixing *k=20* across all benchmarks also isolates the effect of the Stage-2 coreset budget in the comparisons that follow (Section “Multiclass attack detection performance”), since every SSFSS variant and the LA-CGFS (Stage 1) baseline then operate on an identically sized, coverage-saturated feature space.

### Multiclass attack detection performance

Tables 5, 6, 7, 8, 9, 10, 11, 12, 13, 14, 15 and 16 compare SSFSS against both the full-data and Stage 1-only baselines for the four differentiable ERM classifiers introduced in Section “Experimental setup”. All entries are reported as mean ± standard deviation over three independent seeds. The discussion below considers support-weighted metrics alongside macro-F1, balanced accuracy, per-class scores, and retention ratios, so that majority-class performance cannot obscure minority-class degradation. Because each classifier is a differentiable weighted ERM over the shared kernel representation, these results provide a direct empirical test of the conditions under which the error analysis of Section “Error analysis” is derived, and in particular of the two quantities that govern the joint bound (22): the feature distortion $\varepsilon _{\text {feat}}(F_s)$ and the per-class coverage errors $\varepsilon _{\text {samp}}^{(c)}(\mathcal {S}_c \mid F_s)$.

The first consistent pattern concerns Stage 1. At the coverage-saturated budget of *k=20* features (Section “LA-CGFS feature budget”), LA-CGFS is almost lossless across classifiers and datasets. On RT-IoT2022, the Stage 1 baseline matches or exceeds the full-feature baseline for all four models, both on the support-weighted metrics (e.g., KRR accuracy *0.9851 → 0.9873*) and, more markedly, on the minority-sensitive ones (KRR macro-F1 *0.6577 → 0.7038*; SoftmaxLR macro-F1 *0.8519 → 0.8863*) (Table 5). On Edge-IIoTset the largest reduction is 0.30 percentage points (KSVM accuracy *0.9947 → 0.9917*), while KRR again improves (*0.9819 → 0.9831*, with macro-F1 rising *0.9586 → 0.9679*) (Table 9). On CICIoT2023 the three logistic and hinge-type models stay within 0.13 percentage points of accuracy (e.g., SoftmaxLR *0.9871 → 0.9858*), whereas KRR is the single exception across all three benchmarks, losing 1.33 points of accuracy (*0.9606 → 0.9473*) and 3.55 points of macro-F1 (*0.7516 → 0.7161*) (Table 13). This corroborates the diminishing-returns analysis: once the submodular objective is saturated, the discarded features carry almost no class-discriminative information, rendering the feature-distortion term $\varepsilon _{\text {feat}}$ negligible at the selected operating point for every classifier with a bounded loss, and small but non-negligible for the unbounded squared loss on the most heterogeneous benchmark.

The second pattern concerns the Stage 2 coreset, and it differs across datasets in an instructive way. On RT-IoT2022 the per-class coresets *exceed* the full baseline on both the weighted and the minority-sensitive metrics, for all four classifiers. The weighted F1 improves for every model (e.g., KSVM *0.9900 → 0.9945* at SSFSS-5, KRR *0.9827 → 0.9935*, SoftmaxLR *0.9920 → 0.9955* at SSFSS-15, and even KLR *0.9906 → 0.9930* at SSFSS-15), while the macro metrics improve far more dramatically: KSVM macro-F1 rises *0.8166 → 0.9263* and balanced accuracy *0.7788 → 0.9220* at SSFSS-5, KLR balanced accuracy *0.7918 → 0.9453* at SSFSS-10, and KRR—the model most exposed to imbalance—gains 23.6 points of macro-F1 (*0.6577 → 0.8937*) and 21.7 points of balanced accuracy at SSFSS-5 alone (Table 5). Notably, the macro-optimal budget is frequently the *tightest* one (SSFSS-5) rather than the largest, which is the expected signature of the per-class allocation: at the smallest budget the rarest classes receive the largest relative over-allocation. This is not an artifact. On a dataset whose empirical risk is dominated by a single dense class—DOS SYN Hping, whose raw retention falls to $\rho _c^{\text {raw}} = 0.52$ under selection—the square-root per-class allocation compresses the redundant majority region below its natural share while granting the rarest classes several times their proportional mass, so the weighted empirical risk seen by the learner is far better conditioned than the raw one. The coreset thus acts as a principled rebalancing regularizer for differentiable ERMs, an effect that the support-weighted metrics alone would entirely understate.

CICIoT2023—the largest and most heterogeneous benchmark—exhibits the textbook behavior implied by the sample-coverage term: the weighted metrics dip at the tightest budget and recover monotonically as the per-class budgets grow, while the minority-sensitive metrics improve over the full baseline. KSVM weighted F1 rises from 0.9544 (SSFSS-5) to 0.9707 (SSFSS-10) and 0.9705 (SSFSS-15) against a full baseline of 0.9817, and SoftmaxLR follows the same trajectory ($0.9612 \rightarrow 0.9750 \rightarrow 0.9760$ against 0.9865) (Table 13). The macro metrics recover faster and, for three of the four classifiers, overtake the full baseline: KSVM macro-F1 reaches 0.9041 at SSFSS-15 against 0.8784 with all the data, KLR 0.9000 against 0.8843, and KRR improves from 0.7516 to 0.8248 balanced accuracy already by SSFSS-10 (*+7.3* points of macro-F1). Edge-IIoTset, by contrast, is near-lossless and essentially flat in the budget: SoftmaxLR retains 0.9916–0.9927 weighted F1 against 0.9954 full, KSVM 0.9888–0.9918 against 0.9947, and KRR improves its macro-F1 from 0.9586 to 0.9750 at SSFSS-5, with KLR the only model to shed more than a point (weighted F1 *0.9948 → 0.9798* at SSFSS-10) (Table 9).

The retention table makes the structural consequence of per-class selection explicit. Because Stage 2 solves the coverage objective (8) independently within every class and the Voronoi weights of class *c* sum to $n_c$ by construction, the weighted retention satisfies $\rho _c^{\text {w}} = 1$ *exactly* for every class, every budget, and every dataset (Tables 8, 12, 16): the effective mass of each attack class in the weighted empirical risk is preserved identically rather than approximately. The raw prototype-count share, by contrast, is deliberately *not* proportional: the square-root allocation assigns each class a budget proportional to $\sqrt{n_c}$, so dense classes are sub-sampled below their natural share while rare classes are over-covered. On RT-IoT2022 the dominant DOS SYN Hping class is retained at $\rho _c^{\text {raw}} = 0.52$, on Edge-IIoTset the Normal class at 0.69 (and DDoS-ICMP/UDP at *≈ 0.89*–0.90), and on CICIoT2023 the largest DDoS floods between 0.64 and 0.94; symmetrically, mid-rarity classes are lifted to a scale-free multiple of their proportional share that stays roughly constant across budgets (e.g., DDOS Slowloris *≈ 6.9* and NMAP TCP scan *≈ 5.1* on RT-IoT2022; Fingerprinting *≈ 3.4* and MITM *≈ 3.1* on Edge-IIoTset; DNS Spoofing *≈ 4.0* and MITM-ArpSpoofing *≈ 3.0* on CICIoT2023). The only classes whose over-allocation decays with the budget are the ultra-rare ones capped at their full support: on RT-IoT2022, Metasploit Brute Force SSH, NMAP FIN Scan and Wipro Bulb fall from $\rho _c^{\text {raw}} \approx 20$ at SSFSS-5 to *≈ 6.8* at SSFSS-15, and on CICIoT2023 DDoS-SlowLoris and DDoS-HTTP Flood decay from *≈ 10*–11 to *≈ 6.7*. Class-mass preservation is therefore no longer a property to be verified after the fact; the exact preservation of the weighted mass is enforced by construction, and the raw-count profile is a controlled design choice—a deliberate rebalancing—rather than an approximation error.

The per-class tables show where this preserved and rebalanced mass translates into recovered predictive quality. Classes of moderate rarity are the clearest beneficiaries. On RT-IoT2022, the aggregated per-class F1 of DDOS Slowloris rises from 0.5649 (full) to 0.8975 at SSFSS-10, driven by recall (*0.4490 → 0.8990*); Wipro Bulb improves from 0.3959 to 0.8571; Metasploit Brute Force SSH from 0.4963 to 0.7693; and NMAP TCP Scan from 0.9527 to 0.9981 (Tables 6 and 7). On Edge-IIoTset, MITM improves from 0.9760 to 0.9905 at SSFSS-10, and Fingerprinting—the smallest class—trades a little precision for a substantial recall gain (*0.7660 → 0.8566*) and lifts its F1 from 0.8567 to 0.9127 (Tables 10 and 11). On CICIoT2023, the classes that the kernel family finds hardest with all the data improve rather than degrade under compression: DDoS-SlowLoris from 0.3241 to 0.6801, DDoS-HTTP Flood from 0.5318 to 0.8035, MITM-ArpSpoofing from 0.4684 to 0.6291, and DNS Spoofing from 0.3186 to 0.5146 (Table 14). Majority classes are, throughout, essentially unaffected, with per-class F1 changes on the dense classes of all three benchmarks confined to the third decimal.

Two residual failure modes remain, and both are geometric rather than distributional. The first is intra-class coverage under a tight budget: on CICIoT2023 the two large Mirai variants, which are mutually confusable in the reduced feature space, lose substantial F1 at SSFSS-5 (Mirai-greeth *0.9368 → 0.7973*; Mirai-greip *0.9030 → 0.6653*) and recover most of the gap by SSFSS-15 (0.8736 and 0.7529). This is precisely the behavior predicted by the per-class coverage term $\varepsilon _{\text {samp}}^{(c)}$ of Theorem 4.2: preserving a class’s mass does not by itself guarantee that the prototypes resolve its internal structure, and the residual distance defined in (18) shrinks only as the class receives more prototypes. The second failure mode is absolute support: RT-IoT2022 contains classes with roughly 28–37 training samples, for which no coverage statement is meaningful and no budget helps. NMAP FIN Scan swings from 0.2054 (full) up to 0.6989 (SSFSS-5) and back to 0.2714 at SSFSS-10, and Metasploit Brute Force SSH varies between 0.50 and 0.77 without a monotone trend; the dispersion across the four classifiers for these two classes reaches *± 0.36*–0.39 F1 and is as large at full data as on any coreset, so it exceeds the differences between baselines and no ordering among them is statistically meaningful. These observations delimit the minority-class claim precisely: per-class selection guarantees that minority *mass* is preserved exactly and, in most cases, that minority F1 improves through better conditioning; it cannot manufacture separability for classes whose geometry overlaps that of a dense neighbor, nor for classes whose support is too small to be learned from at all—an inherent limitation of coverage-based compression that we acknowledge rather than attempt to mitigate.

Comparing the four losses on the shared representation isolates loss-level effects previously obscured by representation discrepancies. The three logistic and hinge-type models (KLR, KSVM, SoftmaxLR) are nearly interchangeable on the weighted metrics at full data but separate under compression: KSVM is the most resilient, remaining within half a point of its full weighted baseline at every budget on both Edge-IIoTset and RT-IoT2022, while KLR is the most sensitive, accounting for the largest coreset losses on Edge-IIoTset (*0.9948 → 0.9798*) and CICIoT2023 (*0.9824 → 0.9456* at SSFSS-5). KRR, the squared-loss member, is the weakest at full data—most visibly in the macro metrics (macro-F1 0.6577 on RT-IoT2022 and 0.7516 on CICIoT2023)—consistent with the anticipated behavior of an unbounded quadratic loss under pronounced class imbalance. For the same reason, KRR is the classifier that benefits most from SSFSS: its RT-IoT2022 macro-F1 gains 23.6 points at the *5%* budget (*0.6577 → 0.8937*) alongside 21.7 points of balanced accuracy, its CICIoT2023 macro-F1 gains 7.3 points at SSFSS-10, and its Edge-IIoTset macro-F1 rises from 0.9586 to 0.9750. The rebalancing effect of per-class selection is thus maximized precisely for the loss most sensitive to imbalance, in agreement with the weighted-ERM framework of the analysis.

The reported standard deviations delimit which comparisons are resolvable at three seeds. On the support-weighted metrics the conclusions are robust: the seed-to-seed deviation never exceeds 0.93 points of accuracy on RT-IoT2022 and Edge-IIoTset, so the budget-to-budget orderings there are significant; on CICIoT2023 the KLR coreset configurations are the exception, reaching *± 1.00* point of weighted F1 at SSFSS-15, and differences of that magnitude for that model should not be over-interpreted. The macro metrics are intrinsically noisier on RT-IoT2022, where a handful of classes with fewer than 30 samples dominate the macro average and the mean deviation is 2.0–3.6 points for *every* baseline, including the full-data one; crucially, the coreset configurations are *no more* variable than the full-data reference there (the mean macro-F1 deviation across classifiers is comparable at the coreset budgets and at full data, and several coreset settings—e.g., KRR at SSFSS-10, *± 0.0010*—are markedly tighter), so the large rebalancing gains reported above are not an artifact of increased variance. Overall, the findings substantiate a precisely delimited version of the paper’s principal claim: for the differentiable ERM classifiers covered by the theory, a 10–*15%* per-class Voronoi-weighted coreset built on top of 20 LA-CGFS features preserves full-data support-weighted performance to within roughly half a point on Edge-IIoTset (for the three models other than KLR) and within 1.1–1.7 points on CICIoT2023, *exceeds* it on RT-IoT2022 for all four classifiers, and improves the minority-sensitive macro metrics on RT-IoT2022 and CICIoT2023 while leaving them near-lossless on Edge-IIoTset. The residual failure modes are localized, mechanistically explained by the per-class coverage term $\varepsilon _{\text {samp}}^{(c)}$ of the theory, and confined to classes that are either geometrically entangled with a dense neighbor or at the periphery of statistical learnability.

**Table 5:** Performance comparison of classifiers across baselines on RT-IoT2022 (mean ±std over three seeds).

Classifier	Baseline	Acc.	Prec.	Rec.	F1-Score	Macro-F1	Bal. Acc.
KLR	Full Dataset	*0.9913 ± 0.0005*	*0.9911 ± 0.0005*	*0.9913 ± 0.0005*	*0.9906 ± 0.0005*	*0.8278 ± 0.0308*	*0.7918 ± 0.0267*
	LA-CGFS (Stage 1)	*0.9916 ± 0.0014*	*0.9915 ± 0.0015*	*0.9916 ± 0.0014*	*0.9910 ± 0.0016*	*0.8277 ± 0.0594*	*0.7984 ± 0.0659*
	**SSFSS-5 (Stage 2)**	*0.9906 ± 0.0007*	*0.9913 ± 0.0006*	*0.9906 ± 0.0007*	*0.9907 ± 0.0007*	*0.9035 ± 0.0099*	*0.9407 ± 0.0021*
	**SSFSS-10 (Stage 2)**	*0.9915 ± 0.0005*	*0.9924 ± 0.0005*	*0.9915 ± 0.0005*	*0.9918 ± 0.0005*	*0.8818 ± 0.0059*	*0.9453 ± 0.0058*
	**SSFSS-15 (Stage 2)**	*0.9929 ± 0.0004*	*0.9934 ± 0.0001*	*0.9929 ± 0.0004*	*0.9930 ± 0.0003*	*0.9027 ± 0.0159*	*0.9418 ± 0.0017*
KSVM	Full Dataset	*0.9908 ± 0.0008*	*0.9906 ± 0.0008*	*0.9908 ± 0.0008*	*0.9900 ± 0.0009*	*0.8166 ± 0.0273*	*0.7788 ± 0.0214*
	LA-CGFS (Stage 1)	*0.9913 ± 0.0014*	*0.9913 ± 0.0015*	*0.9913 ± 0.0014*	*0.9907 ± 0.0017*	*0.8199 ± 0.0627*	*0.7913 ± 0.0685*
	**SSFSS-5 (Stage 2)**	*0.9945 ± 0.0001*	*0.9946 ± 0.0001*	*0.9945 ± 0.0001*	*0.9945 ± 0.0001*	*0.9263 ± 0.0091*	*0.9220 ± 0.0177*
	**SSFSS-10 (Stage 2)**	*0.9944 ± 0.0007*	*0.9942 ± 0.0007*	*0.9944 ± 0.0007*	*0.9943 ± 0.0007*	*0.8725 ± 0.0028*	*0.8567 ± 0.0036*
	**SSFSS-15 (Stage 2)**	*0.9948 ± 0.0003*	*0.9947 ± 0.0004*	*0.9948 ± 0.0003*	*0.9947 ± 0.0003*	*0.8957 ± 0.0364*	*0.8790 ± 0.0384*
KRR	Full Dataset	*0.9851 ± 0.0027*	*0.9815 ± 0.0031*	*0.9851 ± 0.0027*	*0.9827 ± 0.0027*	*0.6577 ± 0.0194*	*0.6521 ± 0.0186*
	LA-CGFS (Stage 1)	*0.9873 ± 0.0015*	*0.9865 ± 0.0014*	*0.9873 ± 0.0015*	*0.9858 ± 0.0014*	*0.7038 ± 0.0065*	*0.6852 ± 0.0059*
	**SSFSS-5 (Stage 2)**	*0.9936 ± 0.0004*	*0.9936 ± 0.0004*	*0.9936 ± 0.0004*	*0.9935 ± 0.0004*	*0.8937 ± 0.0443*	*0.8694 ± 0.0454*
	**SSFSS-10 (Stage 2)**	*0.9935 ± 0.0006*	*0.9934 ± 0.0006*	*0.9935 ± 0.0006*	*0.9934 ± 0.0006*	*0.8687 ± 0.0010*	*0.8479 ± 0.0011*
	**SSFSS-15 (Stage 2)**	*0.9933 ± 0.0004*	*0.9932 ± 0.0004*	*0.9933 ± 0.0004*	*0.9932 ± 0.0004*	*0.8601 ± 0.0041*	*0.8357 ± 0.0059*
SoftmaxLR	Full Dataset	*0.9924 ± 0.0007*	*0.9922 ± 0.0007*	*0.9924 ± 0.0007*	*0.9920 ± 0.0008*	*0.8519 ± 0.0359*	*0.8246 ± 0.0411*
	LA-CGFS (Stage 1)	*0.9928 ± 0.0012*	*0.9927 ± 0.0012*	*0.9928 ± 0.0012*	*0.9925 ± 0.0013*	*0.8863 ± 0.0333*	*0.8646 ± 0.0409*
	**SSFSS-5 (Stage 2)**	*0.9945 ± 0.0003*	*0.9945 ± 0.0002*	*0.9945 ± 0.0003*	*0.9945 ± 0.0003*	*0.9297 ± 0.0100*	*0.9302 ± 0.0159*
	**SSFSS-10 (Stage 2)**	*0.9949 ± 0.0006*	*0.9950 ± 0.0006*	*0.9949 ± 0.0006*	*0.9949 ± 0.0006*	*0.9359 ± 0.0154*	*0.9416 ± 0.0076*
	**SSFSS-15 (Stage 2)**	*0.9956 ± 0.0003*	*0.9955 ± 0.0004*	*0.9956 ± 0.0003*	*0.9955 ± 0.0004*	*0.9273 ± 0.0392*	*0.9147 ± 0.0431*

The proposed SSFSS at 5%, 10%, and 15% budgets for each classifier is shown in bold.

**Table 6:** Aggregated per-class F1-score (mean ± std over the four classifiers, across three seeds) for RT-IoT2022.

Traffic Class	Full Dataset	LA-CGFS	SSFSS-5	SSFSS-10	SSFSS-15
ARP poisioning	*0.9386 ± 0.0129*	*0.9423 ± 0.0134*	*0.9590 ± 0.0116*	*0.9589 ± 0.0087*	*0.9641 ± 0.0066*
DDOS Slowloris	*0.5649 ± 0.2033*	*0.6605 ± 0.1369*	*0.8735 ± 0.0420*	*0.8975 ± 0.0516*	*0.8850 ± 0.0495*
DOS SYN Hping	*1.0000 ± 0.0000*	*1.0000 ± 0.0000*	*1.0000 ± 0.0000*	*1.0000 ± 0.0000*	*1.0000 ± 0.0000*
MQTT Publish	*0.9956 ± 0.0015*	*0.9934 ± 0.0012*	*0.9962 ± 0.0013*	*0.9958 ± 0.0016*	*0.9955 ± 0.0015*
Metasploit Brute Force SSH	*0.4963 ± 0.2910*	*0.3807 ± 0.2685*	*0.6747 ± 0.1825*	*0.7693 ± 0.0872*	*0.7688 ± 0.0528*
NMAP FIN SCAN	*0.2054 ± 0.3604*	*0.3000 ± 0.4244*	*0.6989 ± 0.1729*	*0.2714 ± 0.3181*	*0.3609 ± 0.3913*
NMAP OS DETECTION	*0.9804 ± 0.0539*	*0.9922 ± 0.0233*	*0.9990 ± 0.0007*	*0.9988 ± 0.0008*	*0.9990 ± 0.0010*
NMAP TCP scan	*0.9527 ± 0.1208*	*0.9697 ± 0.0930*	*0.9960 ± 0.0029*	*0.9981 ± 0.0020*	*0.9981 ± 0.0023*
NMAP UDP SCAN	*0.9703 ± 0.0044*	*0.9636 ± 0.0072*	*0.9515 ± 0.0255*	*0.9620 ± 0.0174*	*0.9622 ± 0.0134*
NMAP XMAS TREE SCAN	*0.9969 ± 0.0015*	*0.9934 ± 0.0087*	*0.9928 ± 0.0031*	*0.9956 ± 0.0022*	*0.9963 ± 0.0013*
Thing Speak	*0.9650 ± 0.0087*	*0.9677 ± 0.0058*	*0.9733 ± 0.0041*	*0.9723 ± 0.0042*	*0.9758 ± 0.0046*
Wipro bulb	*0.3959 ± 0.2340*	*0.5498 ± 0.2087*	*0.8447 ± 0.0555*	*0.8571 ± 0.0217*	*0.8518 ± 0.0348*

**Table 7:** Aggregated per-class Recall (mean ± std over the four classifiers, across three seeds) for RT-IoT2022.

Traffic Class	Full Dataset	LA-CGFS	SSFSS-5	SSFSS-10	SSFSS-15
ARP poisoning	*0.9671 ± 0.0086*	*0.9684 ± 0.0081*	*0.9512 ± 0.0273*	*0.9576 ± 0.0222*	*0.9682 ± 0.0162*
DDOS Slowloris	*0.4490 ± 0.1884*	*0.5229 ± 0.1599*	*0.8599 ± 0.0922*	*0.8990 ± 0.0814*	*0.8661 ± 0.0914*
DOS SYN Hping	*1.0000 ± 0.0000*	*1.0000 ± 0.0000*	*1.0000 ± 0.0001*	*1.0000 ± 0.0000*	*1.0000 ± 0.0000*
MQTT Publish	*0.9914 ± 0.0030*	*0.9871 ± 0.0020*	*0.9939 ± 0.0022*	*0.9934 ± 0.0015*	*0.9930 ± 0.0025*
Metasploit Brute Force SSH	*0.3939 ± 0.2328*	*0.3030 ± 0.2386*	*0.6591 ± 0.1897*	*0.7045 ± 0.0840*	*0.6818 ± 0.0787*
NMAP FIN SCAN	*0.1875 ± 0.3404*	*0.3229 ± 0.4577*	*0.7917 ± 0.2465*	*0.4792 ± 0.4810*	*0.4688 ± 0.4708*
NMAP OS DETECTION	*0.9842 ± 0.0442*	*0.9974 ± 0.0059*	*0.9989 ± 0.0014*	*0.9990 ± 0.0017*	*0.9987 ± 0.0018*
NMAP TCP scan	*0.9488 ± 0.1345*	*0.9615 ± 0.1237*	*0.9994 ± 0.0018*	*0.9994 ± 0.0018*	*0.9983 ± 0.0037*
NMAP UDP SCAN	*0.9780 ± 0.0052*	*0.9670 ± 0.0071*	*0.9595 ± 0.0177*	*0.9605 ± 0.0169*	*0.9612 ± 0.0148*
NMAP XMAS TREE SCAN	*0.9939 ± 0.0028*	*0.9931 ± 0.0043*	*0.9934 ± 0.0025*	*0.9928 ± 0.0044*	*0.9939 ± 0.0029*
Thing Speak	*0.9652 ± 0.0065*	*0.9688 ± 0.0043*	*0.9816 ± 0.0038*	*0.9766 ± 0.0043*	*0.9776 ± 0.0050*
Wipro bulb	*0.2829 ± 0.1722*	*0.4265 ± 0.1830*	*0.7982 ± 0.0822*	*0.8125 ± 0.0540*	*0.8059 ± 0.0462*

**Table 8:** Per-class retention ratios (mean ± std over three seeds) for RT-IoT2022. $\rho _c^{\text {raw}}$ is the raw sample-count retention and $\rho _c^{\text {w}}$ is the Voronoi-weighted retention; a value of 1 denotes exact preservation of a class’s proportion in the coreset.

	SSFSS-5		SSFSS-10		SSFSS-15	
Traffic class	$\rho _c^{\text {raw}}$	$\rho _c^{\text {w}}$	$\rho _c^{\text {raw}}$	$\rho _c^{\text {w}}$	$\rho _c^{\text {raw}}$	$\rho _c^{\text {w}}$
ARP poisioning	*1.8169 ± 0.0000*	*1.0000 ± 0.0000*	*1.8271 ± 0.0000*	*1.0000 ± 0.0000*	*1.8451 ± 0.0000*	*1.0000 ± 0.0000*
DDOS Slowloris	*6.8705 ± 0.0000*	*1.0000 ± 0.0000*	*6.9361 ± 0.0000*	*1.0000 ± 0.0000*	*6.7955 ± 0.0000*	*1.0000 ± 0.0000*
DOS SYN Hping	*0.5199 ± 0.0000*	*1.0000 ± 0.0000*	*0.5228 ± 0.0000*	*1.0000 ± 0.0000*	*0.5280 ± 0.0000*	*1.0000 ± 0.0000*
MQTT Publish	*2.4903 ± 0.0000*	*1.0000 ± 0.0000*	*2.5008 ± 0.0000*	*1.0000 ± 0.0000*	*2.5243 ± 0.0000*	*1.0000 ± 0.0000*
Metasploit Brute Force SSH	*20.0748 ± 0.0000*	*1.0000 ± 0.0000*	*10.0938 ± 0.0000*	*1.0000 ± 0.0000*	*6.7955 ± 0.0000*	*1.0000 ± 0.0000*
NMAP FIN SCAN	*20.0748 ± 0.0000*	*1.0000 ± 0.0000*	*10.0938 ± 0.0000*	*1.0000 ± 0.0000*	*6.7955 ± 0.0000*	*1.0000 ± 0.0000*
NMAP OS DETECTION	*3.5848 ± 0.0000*	*1.0000 ± 0.0000*	*3.5977 ± 0.0000*	*1.0000 ± 0.0000*	*3.6308 ± 0.0000*	*1.0000 ± 0.0000*
NMAP TCP scan	*5.0402 ± 0.0000*	*1.0000 ± 0.0000*	*5.0685 ± 0.0000*	*1.0000 ± 0.0000*	*5.1282 ± 0.0000*	*1.0000 ± 0.0000*
NMAP UDP SCAN	*3.1446 ± 0.0000*	*1.0000 ± 0.0000*	*3.1623 ± 0.0000*	*1.0000 ± 0.0000*	*3.1935 ± 0.0000*	*1.0000 ± 0.0000*
NMAP XMAS TREE SCAN	*3.5812 ± 0.0000*	*1.0000 ± 0.0000*	*3.5942 ± 0.0000*	*1.0000 ± 0.0000*	*3.6224 ± 0.0000*	*1.0000 ± 0.0000*
Thing Speak	*1.7793 ± 0.0000*	*1.0000 ± 0.0000*	*1.7875 ± 0.0000*	*1.0000 ± 0.0000*	*1.8046 ± 0.0000*	*1.0000 ± 0.0000*
Wipro bulb	*9.9807 ± 0.0000*	*1.0000 ± 0.0000*	*10.0938 ± 0.0000*	*1.0000 ± 0.0000*	*6.7955 ± 0.0000*	*1.0000 ± 0.0000*

**Table 9:** Performance comparison of classifiers across baselines on EdgeIIoT-dataset (mean ± std over three seeds).

Classifier	Baseline	Acc.	Prec.	Rec.	F1-Score	Macro-F1	Bal. Acc.
KLR	Full Dataset	*0.9949 ± 0.0002*	*0.9950 ± 0.0002*	*0.9949 ± 0.0002*	*0.9948 ± 0.0002*	*0.9888 ± 0.0002*	*0.9850 ± 0.0004*
	LA-CGFS (Stage 1)	*0.9923 ± 0.0007*	*0.9925 ± 0.0007*	*0.9923 ± 0.0007*	*0.9923 ± 0.0007*	*0.9860 ± 0.0005*	*0.9810 ± 0.0009*
	**SSFSS-5 (Stage 2)**	*0.9877 ± 0.0009*	*0.9878 ± 0.0009*	*0.9877 ± 0.0009*	*0.9877 ± 0.0009*	*0.9826 ± 0.0011*	*0.9800 ± 0.0024*
	**SSFSS-10 (Stage 2)**	*0.9798 ± 0.0059*	*0.9810 ± 0.0049*	*0.9798 ± 0.0059*	*0.9798 ± 0.0059*	*0.9725 ± 0.0068*	*0.9736 ± 0.0061*
	**SSFSS-15 (Stage 2)**	*0.9818 ± 0.0024*	*0.9828 ± 0.0018*	*0.9818 ± 0.0024*	*0.9818 ± 0.0023*	*0.9765 ± 0.0030*	*0.9745 ± 0.0044*
KSVM	Full Dataset	*0.9947 ± 0.0002*	*0.9949 ± 0.0002*	*0.9947 ± 0.0002*	*0.9947 ± 0.0002*	*0.9896 ± 0.0011*	*0.9863 ± 0.0015*
	LA-CGFS (Stage 1)	*0.9917 ± 0.0002*	*0.9919 ± 0.0002*	*0.9917 ± 0.0002*	*0.9917 ± 0.0002*	*0.9854 ± 0.0005*	*0.9804 ± 0.0011*
	**SSFSS-5 (Stage 2)**	*0.9918 ± 0.0007*	*0.9920 ± 0.0007*	*0.9918 ± 0.0007*	*0.9918 ± 0.0007*	*0.9861 ± 0.0020*	*0.9818 ± 0.0031*
	**SSFSS-10 (Stage 2)**	*0.9908 ± 0.0025*	*0.9911 ± 0.0023*	*0.9908 ± 0.0025*	*0.9908 ± 0.0025*	*0.9860 ± 0.0036*	*0.9822 ± 0.0044*
	**SSFSS-15 (Stage 2)**	*0.9888 ± 0.0028*	*0.9893 ± 0.0025*	*0.9888 ± 0.0028*	*0.9888 ± 0.0028*	*0.9827 ± 0.0040*	*0.9777 ± 0.0051*
KRR	Full Dataset	*0.9819 ± 0.0021*	*0.9825 ± 0.0021*	*0.9819 ± 0.0021*	*0.9814 ± 0.0022*	*0.9586 ± 0.0077*	*0.9488 ± 0.0127*
	LA-CGFS (Stage 1)	*0.9831 ± 0.0046*	*0.9840 ± 0.0042*	*0.9831 ± 0.0046*	*0.9830 ± 0.0046*	*0.9679 ± 0.0088*	*0.9588 ± 0.0138*
	**SSFSS-5 (Stage 2)**	*0.9832 ± 0.0015*	*0.9837 ± 0.0014*	*0.9832 ± 0.0015*	*0.9832 ± 0.0015*	*0.9750 ± 0.0021*	*0.9708 ± 0.0030*
	**SSFSS-10 (Stage 2)**	*0.9807 ± 0.0048*	*0.9816 ± 0.0044*	*0.9807 ± 0.0048*	*0.9808 ± 0.0047*	*0.9716 ± 0.0047*	*0.9668 ± 0.0063*
	**SSFSS-15 (Stage 2)**	*0.9737 ± 0.0105*	*0.9758 ± 0.0097*	*0.9737 ± 0.0105*	*0.9740 ± 0.0103*	*0.9635 ± 0.0141*	*0.9556 ± 0.0165*
SoftmaxLR	Full Dataset	*0.9954 ± 0.0002*	*0.9956 ± 0.0002*	*0.9954 ± 0.0002*	*0.9954 ± 0.0002*	*0.9902 ± 0.0012*	*0.9869 ± 0.0015*
	LA-CGFS (Stage 1)	*0.9928 ± 0.0008*	*0.9930 ± 0.0007*	*0.9928 ± 0.0008*	*0.9928 ± 0.0007*	*0.9867 ± 0.0005*	*0.9816 ± 0.0008*
	**SSFSS-5 (Stage 2)**	*0.9927 ± 0.0012*	*0.9928 ± 0.0012*	*0.9927 ± 0.0012*	*0.9926 ± 0.0012*	*0.9869 ± 0.0027*	*0.9823 ± 0.0037*
	**SSFSS-10 (Stage 2)**	*0.9918 ± 0.0025*	*0.9921 ± 0.0023*	*0.9918 ± 0.0025*	*0.9918 ± 0.0025*	*0.9873 ± 0.0035*	*0.9837 ± 0.0044*
	**SSFSS-15 (Stage 2)**	*0.9916 ± 0.0023*	*0.9919 ± 0.0021*	*0.9916 ± 0.0023*	*0.9916 ± 0.0022*	*0.9866 ± 0.0029*	*0.9834 ± 0.0040*

The proposed SSFSS at 5%, 10%, and 15% budgets for each classifier is shown in bold.

**Table 10:** Aggregated per-class F1-score (mean ± std over the four classifiers, across three seeds) for EdgeIIoT-dataset.

Traffic Class	Full Dataset	LA-CGFS	SSFSS-5	SSFSS-10	SSFSS-15
Backdoor	*0.9769 ± 0.0057*	*0.9792 ± 0.0073*	*0.9777 ± 0.0047*	*0.9746 ± 0.0085*	*0.9738 ± 0.0058*
DDoS HTTP	*0.9927 ± 0.0077*	*0.9809 ± 0.0042*	*0.9758 ± 0.0081*	*0.9817 ± 0.0089*	*0.9747 ± 0.0102*
DDoS ICMP	*0.9982 ± 0.0029*	*0.9999 ± 0.0001*	*0.9992 ± 0.0012*	*0.9995 ± 0.0008*	*0.9998 ± 0.0002*
DDoS TCP	*1.0000 ± 0.0000*	*0.9999 ± 0.0001*	*0.9999 ± 0.0001*	*0.9998 ± 0.0005*	*0.9999 ± 0.0002*
DDoS UDP	*1.0000 ± 0.0000*	*1.0000 ± 0.0000*	*1.0000 ± 0.0000*	*1.0000 ± 0.0000*	*1.0000 ± 0.0000*
Fingerprinting	*0.8567 ± 0.1170*	*0.8883 ± 0.0447*	*0.9076 ± 0.0230*	*0.8894 ± 0.0522*	*0.9127 ± 0.0249*
MITM	*0.9760 ± 0.0292*	*0.9676 ± 0.0471*	*0.9858 ± 0.0230*	*0.9905 ± 0.0123*	*0.9705 ± 0.0432*
Normal	*0.9963 ± 0.0066*	*0.9963 ± 0.0022*	*0.9953 ± 0.0051*	*0.9892 ± 0.0156*	*0.9938 ± 0.0061*
Password	*0.9914 ± 0.0087*	*0.9809 ± 0.0077*	*0.9766 ± 0.0073*	*0.9818 ± 0.0088*	*0.9775 ± 0.0057*
Port Scanning	*0.9916 ± 0.0021*	*0.9915 ± 0.0017*	*0.9879 ± 0.0064*	*0.9792 ± 0.0167*	*0.9857 ± 0.0125*
Ransomware	*0.9762 ± 0.0080*	*0.9707 ± 0.0149*	*0.9753 ± 0.0082*	*0.9533 ± 0.0260*	*0.9540 ± 0.0254*
SQL injection	*0.9947 ± 0.0104*	*0.9981 ± 0.0015*	*0.9944 ± 0.0078*	*0.9963 ± 0.0049*	*0.9891 ± 0.0189*
Uploading	*0.9944 ± 0.0111*	*0.9959 ± 0.0057*	*0.9907 ± 0.0082*	*0.9892 ± 0.0156*	*0.9742 ± 0.0353*
Vulnerability scanner	*0.9925 ± 0.0079*	*0.9909 ± 0.0099*	*0.9870 ± 0.0085*	*0.9890 ± 0.0103*	*0.9883 ± 0.0114*
XSS	*0.9892 ± 0.0118*	*0.9820 ± 0.0166*	*0.9865 ± 0.0079*	*0.9767 ± 0.0163*	*0.9661 ± 0.0319*

**Table 11:** Aggregated per-class Recall (mean ± std over the four classifiers, across three seeds) for EdgeIIoT-dataset.

Traffic Class	Full Dataset	LA-CGFS	SSFSS-5	SSFSS-10	SSFSS-15
Backdoor	*0.9550 ± 0.0107*	*0.9598 ± 0.0130*	*0.9589 ± 0.0106*	*0.9568 ± 0.0092*	*0.9495 ± 0.0109*
DDoS HTTP	*0.9921 ± 0.0123*	*0.9680 ± 0.0084*	*0.9632 ± 0.0134*	*0.9776 ± 0.0134*	*0.9644 ± 0.0076*
DDoS ICMP	*0.9998 ± 0.0001*	*0.9998 ± 0.0002*	*0.9986 ± 0.0024*	*0.9990 ± 0.0015*	*0.9997 ± 0.0003*
DDoS TCP	*1.0000 ± 0.0000*	*0.9999 ± 0.0001*	*0.9999 ± 0.0003*	*0.9997 ± 0.0009*	*0.9998 ± 0.0004*
DDoS UDP	*1.0000 ± 0.0000*	*1.0000 ± 0.0000*	*1.0000 ± 0.0000*	*1.0000 ± 0.0000*	*1.0000 ± 0.0000*
Fingerprinting	*0.7660 ± 0.1571*	*0.8039 ± 0.0671*	*0.8558 ± 0.0316*	*0.8617 ± 0.0519*	*0.8566 ± 0.0426*
MITM	*0.9836 ± 0.0544*	*0.9492 ± 0.0815*	*0.9730 ± 0.0437*	*0.9815 ± 0.0238*	*0.9511 ± 0.0794*
Normal	*0.9972 ± 0.0023*	*0.9945 ± 0.0025*	*0.9961 ± 0.0023*	*0.9943 ± 0.0022*	*0.9924 ± 0.0088*
Password	*0.9915 ± 0.0057*	*0.9878 ± 0.0118*	*0.9881 ± 0.0062*	*0.9848 ± 0.0071*	*0.9882 ± 0.0058*
Port Scanning	*1.0000 ± 0.0000*	*1.0000 ± 0.0001*	*0.9987 ± 0.0022*	*0.9738 ± 0.0366*	*0.9859 ± 0.0264*
Ransomware	*0.9954 ± 0.0142*	*0.9997 ± 0.0003*	*0.9898 ± 0.0183*	*0.9694 ± 0.0471*	*0.9737 ± 0.0387*
SQL injection	*0.9948 ± 0.0080*	*0.9983 ± 0.0002*	*0.9981 ± 0.0004*	*0.9985 ± 0.0005*	*0.9832 ± 0.0325*
Uploading	*0.9934 ± 0.0151*	*0.9949 ± 0.0074*	*0.9884 ± 0.0151*	*0.9928 ± 0.0102*	*0.9938 ± 0.0093*
Vulnerability scanner	*0.9879 ± 0.0113*	*0.9859 ± 0.0166*	*0.9784 ± 0.0152*	*0.9837 ± 0.0184*	*0.9815 ± 0.0188*
XSS	*0.9941 ± 0.0089*	*0.9904 ± 0.0130*	*0.9942 ± 0.0029*	*0.9752 ± 0.0227*	*0.9724 ± 0.0346*

**Table 12:** Per-class retention ratios (mean ± std over three seeds) for EdgeIIoT-dataset. $\rho _c^{\text {raw}}$ is the raw sample-count retention and $\rho _c^{\text {w}}$ is the Voronoi-weighted retention; a value of 1 denotes exact preservation of a class’s proportion in the coreset.

	SSFSS-5		SSFSS-10		SSFSS-15	
Traffic Class	$\rho _c^{\text {raw}}$	$\rho _c^{\text {w}}$	$\rho _c^{\text {raw}}$	$\rho _c^{\text {w}}$	$\rho _c^{\text {raw}}$	$\rho _c^{\text {w}}$
Backdoor	*1.0587 ± 0.0000*	*1.0000 ± 0.0000*	*1.0587 ± 0.0000*	*1.0000 ± 0.0000*	*1.0597 ± 0.0000*	*1.0000 ± 0.0000*
DDoS HTTP	*1.0440 ± 0.0000*	*1.0000 ± 0.0000*	*1.0426 ± 0.0000*	*1.0000 ± 0.0000*	*1.0413 ± 0.0000*	*1.0000 ± 0.0000*
DDoS ICMP	*0.9033 ± 0.0000*	*1.0000 ± 0.0000*	*0.9023 ± 0.0000*	*1.0000 ± 0.0000*	*0.9020 ± 0.0000*	*1.0000 ± 0.0000*
DDoS TCP	*1.0560 ± 0.0000*	*1.0000 ± 0.0000*	*1.0560 ± 0.0000*	*1.0000 ± 0.0000*	*1.0561 ± 0.0000*	*1.0000 ± 0.0000*
DDoS UDP	*0.8898 ± 0.0000*	*1.0000 ± 0.0000*	*0.8888 ± 0.0000*	*1.0000 ± 0.0000*	*0.8892 ± 0.0000*	*1.0000 ± 0.0000*
Fingerprinting	*3.3716 ± 0.0000*	*1.0000 ± 0.0000*	*3.3716 ± 0.0000*	*1.0000 ± 0.0000*	*3.3718 ± 0.0000*	*1.0000 ± 0.0000*
MITM	*3.0676 ± 0.0000*	*1.0000 ± 0.0000*	*3.0676 ± 0.0000*	*1.0000 ± 0.0000*	*3.0678 ± 0.0000*	*1.0000 ± 0.0000*
Normal	*0.6864 ± 0.0000*	*1.0000 ± 0.0000*	*0.6864 ± 0.0000*	*1.0000 ± 0.0000*	*0.6864 ± 0.0000*	*1.0000 ± 0.0000*
Password	*1.0690 ± 0.0000*	*1.0000 ± 0.0000*	*1.0705 ± 0.0000*	*1.0000 ± 0.0000*	*1.0700 ± 0.0000*	*1.0000 ± 0.0000*
Port Scanning	*1.0660 ± 0.0000*	*1.0000 ± 0.0000*	*1.0660 ± 0.0000*	*1.0000 ± 0.0000*	*1.0661 ± 0.0000*	*1.0000 ± 0.0000*
Ransomware	*1.0249 ± 0.0000*	*1.0000 ± 0.0000*	*1.0249 ± 0.0000*	*1.0000 ± 0.0000*	*1.0241 ± 0.0000*	*1.0000 ± 0.0000*
SQL injection	*1.0551 ± 0.0000*	*1.0000 ± 0.0000*	*1.0551 ± 0.0000*	*1.0000 ± 0.0000*	*1.0542 ± 0.0000*	*1.0000 ± 0.0000*
Uploading	*1.0539 ± 0.0000*	*1.0000 ± 0.0000*	*1.0553 ± 0.0000*	*1.0000 ± 0.0000*	*1.0568 ± 0.0000*	*1.0000 ± 0.0000*
Vulnerability scanner	*1.0654 ± 0.0000*	*1.0000 ± 0.0000*	*1.0654 ± 0.0000*	*1.0000 ± 0.0000*	*1.0655 ± 0.0000*	*1.0000 ± 0.0000*
XSS	*1.0651 ± 0.0000*	*1.0000 ± 0.0000*	*1.0666 ± 0.0000*	*1.0000 ± 0.0000*	*1.0671 ± 0.0000*	*1.0000 ± 0.0000*

**Table 13:** Performance comparison of classifiers across baselines on CICIoT2023 (mean ± std over three seeds).

Classifier	Baseline	Acc.	Prec.	Rec.	F1-Score	Macro-F1	Bal. Acc.
KLR	Full Dataset	*0.9833 ± 0.0002*	*0.9831 ± 0.0003*	*0.9833 ± 0.0002*	*0.9824 ± 0.0003*	*0.8843 ± 0.0089*	*0.8748 ± 0.0088*
	LA-CGFS (Stage 1)	*0.9829 ± 0.0013*	*0.9829 ± 0.0010*	*0.9829 ± 0.0013*	*0.9820 ± 0.0014*	*0.8833 ± 0.0038*	*0.8669 ± 0.0083*
	**SSFSS-5 (Stage 2)**	*0.9466 ± 0.0027*	*0.9484 ± 0.0036*	*0.9466 ± 0.0027*	*0.9456 ± 0.0038*	*0.8600 ± 0.0043*	*0.8535 ± 0.0059*
	**SSFSS-10 (Stage 2)**	*0.9693 ± 0.0083*	*0.9696 ± 0.0080*	*0.9693 ± 0.0083*	*0.9687 ± 0.0090*	*0.8955 ± 0.0065*	*0.8934 ± 0.0061*
	**SSFSS-15 (Stage 2)**	*0.9655 ± 0.0093*	*0.9666 ± 0.0091*	*0.9655 ± 0.0093*	*0.9651 ± 0.0100*	*0.9000 ± 0.0044*	*0.8992 ± 0.0076*
KSVM	Full Dataset	*0.9828 ± 0.0004*	*0.9826 ± 0.0005*	*0.9828 ± 0.0004*	*0.9817 ± 0.0005*	*0.8784 ± 0.0106*	*0.8694 ± 0.0099*
	LA-CGFS (Stage 1)	*0.9819 ± 0.0018*	*0.9820 ± 0.0016*	*0.9819 ± 0.0018*	*0.9808 ± 0.0020*	*0.8731 ± 0.0149*	*0.8562 ± 0.0180*
	**SSFSS-5 (Stage 2)**	*0.9546 ± 0.0086*	*0.9558 ± 0.0079*	*0.9546 ± 0.0086*	*0.9544 ± 0.0090*	*0.8709 ± 0.0092*	*0.8739 ± 0.0068*
	**SSFSS-10 (Stage 2)**	*0.9712 ± 0.0055*	*0.9710 ± 0.0058*	*0.9712 ± 0.0055*	*0.9707 ± 0.0058*	*0.8982 ± 0.0034*	*0.8966 ± 0.0023*
	**SSFSS-15 (Stage 2)**	*0.9710 ± 0.0082*	*0.9712 ± 0.0078*	*0.9710 ± 0.0082*	*0.9705 ± 0.0086*	*0.9041 ± 0.0047*	*0.9011 ± 0.0071*
KRR	Full Dataset	*0.9606 ± 0.0010*	*0.9584 ± 0.0010*	*0.9606 ± 0.0010*	*0.9569 ± 0.0013*	*0.7516 ± 0.0096*	*0.7477 ± 0.0082*
	LA-CGFS (Stage 1)	*0.9473 ± 0.0039*	*0.9453 ± 0.0058*	*0.9473 ± 0.0039*	*0.9420 ± 0.0049*	*0.7161 ± 0.0035*	*0.7171 ± 0.0027*
	**SSFSS-5 (Stage 2)**	*0.9343 ± 0.0063*	*0.9363 ± 0.0076*	*0.9343 ± 0.0063*	*0.9338 ± 0.0072*	*0.8070 ± 0.0166*	*0.8005 ± 0.0176*
	**SSFSS-10 (Stage 2)**	*0.9486 ± 0.0053*	*0.9480 ± 0.0050*	*0.9486 ± 0.0053*	*0.9463 ± 0.0062*	*0.8248 ± 0.0108*	*0.8134 ± 0.0114*
	**SSFSS-15 (Stage 2)**	*0.9478 ± 0.0062*	*0.9474 ± 0.0053*	*0.9478 ± 0.0062*	*0.9451 ± 0.0070*	*0.8056 ± 0.0099*	*0.7924 ± 0.0112*
SoftmaxLR	Full Dataset	*0.9871 ± 0.0003*	*0.9868 ± 0.0003*	*0.9871 ± 0.0003*	*0.9865 ± 0.0003*	*0.9053 ± 0.0106*	*0.8994 ± 0.0104*
	LA-CGFS (Stage 1)	*0.9858 ± 0.0009*	*0.9857 ± 0.0009*	*0.9858 ± 0.0009*	*0.9852 ± 0.0011*	*0.9042 ± 0.0067*	*0.8967 ± 0.0075*
	**SSFSS-5 (Stage 2)**	*0.9613 ± 0.0099*	*0.9620 ± 0.0095*	*0.9613 ± 0.0099*	*0.9612 ± 0.0101*	*0.8838 ± 0.0061*	*0.8852 ± 0.0062*
	**SSFSS-10 (Stage 2)**	*0.9751 ± 0.0038*	*0.9751 ± 0.0039*	*0.9751 ± 0.0038*	*0.9750 ± 0.0038*	*0.9131 ± 0.0037*	*0.9126 ± 0.0042*
	**SSFSS-15 (Stage 2)**	*0.9762 ± 0.0054*	*0.9764 ± 0.0052*	*0.9762 ± 0.0054*	*0.9760 ± 0.0055*	*0.9162 ± 0.0066*	*0.9147 ± 0.0079*

The proposed SSFSS at 5%, 10%, and 15% budgets for each classifier is shown in bold.

**Table 14:** Aggregated per-class F1-score (mean ± std over the four classifiers, across three seeds) for CICIoT2023.

Traffic Class	Full Dataset	LA-CGFS	SSFSS-5	SSFSS-10	SSFSS-15
BenignTraffic	*0.8415 ± 0.0270*	*0.8438 ± 0.0404*	*0.8710 ± 0.0102*	*0.8869 ± 0.0166*	*0.8895 ± 0.0185*
DDoS-ACK Fragmentation	*0.9258 ± 0.0887*	*0.8133 ± 0.2252*	*0.8221 ± 0.1788*	*0.8645 ± 0.1327*	*0.8809 ± 0.1313*
DDoS-HTTP Flood	*0.5318 ± 0.3173*	*0.4909 ± 0.2923*	*0.7728 ± 0.0744*	*0.8035 ± 0.0752*	*0.7859 ± 0.1083*
DDoS-ICMP Flood	*0.9990 ± 0.0002*	*0.9954 ± 0.0044*	*0.9878 ± 0.0121*	*0.9935 ± 0.0064*	*0.9927 ± 0.0077*
DDoS-ICMP Fragmentation	*0.9840 ± 0.0081*	*0.9121 ± 0.0688*	*0.9157 ± 0.0667*	*0.9229 ± 0.0614*	*0.9291 ± 0.0635*
DDoS-PSHACK Flood	*0.9978 ± 0.0021*	*0.9923 ± 0.0086*	*0.9858 ± 0.0139*	*0.9891 ± 0.0112*	*0.9880 ± 0.0123*
DDoS-RSTFINFlood	*0.9984 ± 0.0013*	*0.9958 ± 0.0033*	*0.9967 ± 0.0026*	*0.9975 ± 0.0009*	*0.9969 ± 0.0022*
DDoS-SYN Flood	*0.9707 ± 0.0183*	*0.9758 ± 0.0222*	*0.8839 ± 0.0432*	*0.9360 ± 0.0198*	*0.9340 ± 0.0272*
DDoS-SlowLoris	*0.3241 ± 0.2538*	*0.4075 ± 0.2550*	*0.5619 ± 0.0827*	*0.6801 ± 0.0882*	*0.6049 ± 0.2196*
DDoS-SynonymousIP Flood	*0.9781 ± 0.0077*	*0.9849 ± 0.0090*	*0.9112 ± 0.0300*	*0.9435 ± 0.0162*	*0.9319 ± 0.0360*
DDoS-TCP Flood	*0.9941 ± 0.0062*	*0.9873 ± 0.0148*	*0.9727 ± 0.0112*	*0.9817 ± 0.0122*	*0.9773 ± 0.0114*
DDoS-UDP Flood	*0.9962 ± 0.0027*	*0.9963 ± 0.0036*	*0.9946 ± 0.0017*	*0.9961 ± 0.0021*	*0.9957 ± 0.0023*
DDoS-UDP Fragmentation	*0.9555 ± 0.0509*	*0.9530 ± 0.0552*	*0.9596 ± 0.0104*	*0.9716 ± 0.0161*	*0.9716 ± 0.0210*
DNS Spoofing	*0.3186 ± 0.1257*	*0.4071 ± 0.1422*	*0.4824 ± 0.0894*	*0.5146 ± 0.0888*	*0.5123 ± 0.1325*
DoS-HTTP Flood	*0.7405 ± 0.1893*	*0.6668 ± 0.2214*	*0.7859 ± 0.0628*	*0.8072 ± 0.0654*	*0.8195 ± 0.0750*
DoS-SYN Flood	*0.9726 ± 0.0296*	*0.9714 ± 0.0277*	*0.9036 ± 0.0647*	*0.9613 ± 0.0293*	*0.9699 ± 0.0203*
DoS-TCP Flood	*0.9912 ± 0.0102*	*0.9831 ± 0.0204*	*0.9667 ± 0.0172*	*0.9807 ± 0.0092*	*0.9724 ± 0.0150*
DoS-UDP Flood	*0.9918 ± 0.0078*	*0.9931 ± 0.0070*	*0.9899 ± 0.0028*	*0.9919 ± 0.0036*	*0.9926 ± 0.0039*
MITM-ArpSpoofing	*0.4684 ± 0.1463*	*0.4932 ± 0.1337*	*0.6030 ± 0.0345*	*0.6138 ± 0.0417*	*0.6291 ± 0.0660*
Mirai-greeth flood	*0.9368 ± 0.0510*	*0.9075 ± 0.0801*	*0.7973 ± 0.1115*	*0.8573 ± 0.1002*	*0.8736 ± 0.1001*
Mirai-greip flood	*0.9030 ± 0.0880*	*0.8099 ± 0.2165*	*0.6653 ± 0.2010*	*0.7385 ± 0.2165*	*0.7529 ± 0.2157*
Mirai-udpplain	*0.9882 ± 0.0122*	*0.9917 ± 0.0061*	*0.9890 ± 0.0027*	*0.9917 ± 0.0036*	*0.9913 ± 0.0036*

**Table 15:** Aggregated per-class Recall (mean ± std over the four classifiers, across three seeds) for CICIoT2023.

Traffic class	Full dataset	LA-CGFS	SSFSS-5	SSFSS-10	SSFSS-15
BenignTraffic	*0.9571 ± 0.0103*	*0.9485 ± 0.0294*	*0.8877 ± 0.0280*	*0.9173 ± 0.0220*	*0.9228 ± 0.0268*
DDoS-ACK Fragmentation	*0.9080 ± 0.1249*	*0.7732 ± 0.2434*	*0.7960 ± 0.2022*	*0.8472 ± 0.1602*	*0.8689 ± 0.1554*
DDoS-HTTP Flood	*0.5109 ± 0.3111*	*0.4565 ± 0.2815*	*0.7681 ± 0.1127*	*0.7971 ± 0.1259*	*0.7862 ± 0.1592*
DDoS-ICMP Flood	*0.9980 ± 0.0004*	*0.9945 ± 0.0040*	*0.9861 ± 0.0163*	*0.9954 ± 0.0027*	*0.9948 ± 0.0046*
DDoS-ICMP Fragmentation	*0.9828 ± 0.0057*	*0.9382 ± 0.0313*	*0.9230 ± 0.0334*	*0.9299 ± 0.0413*	*0.9323 ± 0.0444*
DDoS-PSHACK Flood	*0.9988 ± 0.0007*	*0.9956 ± 0.0044*	*0.9817 ± 0.0210*	*0.9837 ± 0.0183*	*0.9874 ± 0.0150*
DDoS-RSTFINFlood	*0.9983 ± 0.0005*	*0.9976 ± 0.0011*	*0.9965 ± 0.0047*	*0.9983 ± 0.0005*	*0.9968 ± 0.0040*
DDoS-SYN Flood	*0.9601 ± 0.0193*	*0.9749 ± 0.0174*	*0.8906 ± 0.0867*	*0.9426 ± 0.0095*	*0.9595 ± 0.0098*
DDoS-SlowLoris	*0.2851 ± 0.2454*	*0.3684 ± 0.2578*	*0.5965 ± 0.0755*	*0.7105 ± 0.1206*	*0.6096 ± 0.2268*
DDoS-SynonymousIP Flood	*0.9894 ± 0.0043*	*0.9912 ± 0.0058*	*0.9112 ± 0.0248*	*0.9425 ± 0.0293*	*0.9099 ± 0.0599*
DDoS-TCP Flood	*0.9957 ± 0.0043*	*0.9854 ± 0.0176*	*0.9814 ± 0.0071*	*0.9865 ± 0.0080*	*0.9724 ± 0.0184*
DDoS-UDP Flood	*0.9957 ± 0.0030*	*0.9973 ± 0.0018*	*0.9961 ± 0.0017*	*0.9970 ± 0.0017*	*0.9972 ± 0.0016*
DDoS-UDP Fragmentation	*0.9578 ± 0.0502*	*0.9585 ± 0.0502*	*0.9627 ± 0.0197*	*0.9754 ± 0.0198*	*0.9696 ± 0.0281*
DNS Spoofing	*0.2301 ± 0.1052*	*0.3013 ± 0.1285*	*0.4581 ± 0.1279*	*0.4829 ± 0.1338*	*0.4768 ± 0.1778*
DoS-HTTP Flood	*0.7315 ± 0.2137*	*0.6362 ± 0.2083*	*0.7593 ± 0.0766*	*0.7606 ± 0.0953*	*0.7804 ± 0.1184*
DoS-SYN Flood	*0.9691 ± 0.0362*	*0.9663 ± 0.0398*	*0.8987 ± 0.0927*	*0.9548 ± 0.0496*	*0.9628 ± 0.0324*
DoS-TCP Flood	*0.9902 ± 0.0114*	*0.9833 ± 0.0201*	*0.9610 ± 0.0349*	*0.9823 ± 0.0127*	*0.9840 ± 0.0154*
DoS-UDP Flood	*0.9946 ± 0.0033*	*0.9949 ± 0.0036*	*0.9876 ± 0.0058*	*0.9907 ± 0.0032*	*0.9922 ± 0.0059*
MITM-ArpSpoofing	*0.3733 ± 0.1404*	*0.3859 ± 0.1293*	*0.5707 ± 0.0580*	*0.5612 ± 0.0667*	*0.5802 ± 0.0976*
Mirai-greeth flood	*0.9422 ± 0.0276*	*0.9438 ± 0.0292*	*0.8181 ± 0.0953*	*0.8804 ± 0.0744*	*0.8959 ± 0.0663*
Mirai-greip flood	*0.8899 ± 0.1232*	*0.7703 ± 0.2561*	*0.6519 ± 0.2242*	*0.7104 ± 0.2693*	*0.7197 ± 0.2713*
Mirai-udpplain	*0.9935 ± 0.0028*	*0.9914 ± 0.0065*	*0.9893 ± 0.0063*	*0.9910 ± 0.0062*	*0.9912 ± 0.0065*

**Table 16:** Per-class retention ratios (mean ± std over three seeds) for CICIoT2023. $\rho _c^{\text {raw}}$ is the raw sample-count retention and $\rho _c^{\text {w}}$ is the Voronoi-weighted retention; a value of 1 denotes exact preservation of a class’s proportion in the coreset.

	SSFSS-5		SSFSS-10		SSFSS-15	
Traffic class	$\rho _c^{\text {raw}}$	$\rho _c^{\text {w}}$	$\rho _c^{\text {raw}}$	$\rho _c^{\text {w}}$	$\rho _c^{\text {raw}}$	$\rho _c^{\text {w}}$
BenignTraffic	*1.6433 ± 0.0000*	*1.0000 ± 0.0000*	*1.6399 ± 0.0000*	*1.0000 ± 0.0000*	*1.6412 ± 0.0000*	*1.0000 ± 0.0000*
DDoS-ACK Fragmentation	*3.1616 ± 0.0000*	*1.0000 ± 0.0000*	*3.1818 ± 0.0000*	*1.0000 ± 0.0000*	*3.1994 ± 0.0000*	*1.0000 ± 0.0000*
DDoS-HTTP Flood	*10.1818 ± 0.0000*	*1.0000 ± 0.0000*	*10.0077 ± 0.0000*	*1.0000 ± 0.0000*	*6.6961 ± 0.0000*	*1.0000 ± 0.0000*
DDoS-ICMP Flood	*0.6378 ± 0.0000*	*1.0000 ± 0.0000*	*0.6375 ± 0.0000*	*1.0000 ± 0.0000*	*0.6401 ± 0.0000*	*1.0000 ± 0.0000*
DDoS-ICMP Fragmentation	*2.5251 ± 0.0000*	*1.0000 ± 0.0000*	*2.5271 ± 0.0000*	*1.0000 ± 0.0000*	*2.5363 ± 0.0000*	*1.0000 ± 0.0000*
DDoS-PSHACK Flood	*0.8436 ± 0.0000*	*1.0000 ± 0.0000*	*0.8431 ± 0.0000*	*1.0000 ± 0.0000*	*0.8457 ± 0.0000*	*1.0000 ± 0.0000*
DDoS-RSTFINFlood	*0.8506 ± 0.0000*	*1.0000 ± 0.0000*	*0.8500 ± 0.0000*	*1.0000 ± 0.0000*	*0.8527 ± 0.0000*	*1.0000 ± 0.0000*
DDoS-SYN Flood	*0.8455 ± 0.0000*	*1.0000 ± 0.0000*	*0.8461 ± 0.0000*	*1.0000 ± 0.0000*	*0.8492 ± 0.0000*	*1.0000 ± 0.0000*
DDoS-SlowLoris	*11.1628 ± 0.0000*	*1.0000 ± 0.0000*	*10.0077 ± 0.0000*	*1.0000 ± 0.0000*	*6.6961 ± 0.0000*	*1.0000 ± 0.0000*
DDoS-SynonymousIP Flood	*0.9010 ± 0.0000*	*1.0000 ± 0.0000*	*0.9017 ± 0.0000*	*1.0000 ± 0.0000*	*0.9050 ± 0.0000*	*1.0000 ± 0.0000*
DDoS-TCP Flood	*0.8056 ± 0.0000*	*1.0000 ± 0.0000*	*0.8051 ± 0.0000*	*1.0000 ± 0.0000*	*0.8076 ± 0.0000*	*1.0000 ± 0.0000*
DDoS-UDP Flood	*0.7354 ± 0.0000*	*1.0000 ± 0.0000*	*0.7360 ± 0.0000*	*1.0000 ± 0.0000*	*0.7380 ± 0.0000*	*1.0000 ± 0.0000*
DDoS-UDP Fragmentation	*3.1616 ± 0.0000*	*1.0000 ± 0.0000*	*3.1818 ± 0.0000*	*1.0000 ± 0.0000*	*3.1994 ± 0.0000*	*1.0000 ± 0.0000*
DNS Spoofing	*4.0227 ± 0.0000*	*1.0000 ± 0.0000*	*4.0258 ± 0.0000*	*1.0000 ± 0.0000*	*4.0404 ± 0.0000*	*1.0000 ± 0.0000*
DoS-HTTP Flood	*6.1224 ± 0.0000*	*1.0000 ± 0.0000*	*6.1952 ± 0.0000*	*1.0000 ± 0.0000*	*6.2406 ± 0.0000*	*1.0000 ± 0.0000*
DoS-SYN Flood	*1.2032 ± 0.0000*	*1.0000 ± 0.0000*	*1.2042 ± 0.0000*	*1.0000 ± 0.0000*	*1.2085 ± 0.0000*	*1.0000 ± 0.0000*
DoS-TCP Flood	*1.0472 ± 0.0000*	*1.0000 ± 0.0000*	*1.0480 ± 0.0000*	*1.0000 ± 0.0000*	*1.0505 ± 0.0000*	*1.0000 ± 0.0000*
DoS-UDP Flood	*0.9354 ± 0.0000*	*1.0000 ± 0.0000*	*0.9361 ± 0.0000*	*1.0000 ± 0.0000*	*0.9395 ± 0.0000*	*1.0000 ± 0.0000*
MITM-ArpSpoofing	*3.0293 ± 0.0000*	*1.0000 ± 0.0000*	*3.0479 ± 0.0000*	*1.0000 ± 0.0000*	*3.0645 ± 0.0000*	*1.0000 ± 0.0000*
Mirai-greeth flood	*1.7261 ± 0.0000*	*1.0000 ± 0.0000*	*1.7171 ± 0.0000*	*1.0000 ± 0.0000*	*1.7268 ± 0.0000*	*1.0000 ± 0.0000*
Mirai-greip flood	*1.9555 ± 0.0000*	*1.0000 ± 0.0000*	*1.9637 ± 0.0000*	*1.0000 ± 0.0000*	*1.9732 ± 0.0000*	*1.0000 ± 0.0000*
Mirai-udpplain	*1.7904 ± 0.0000*	*1.0000 ± 0.0000*	*1.7974 ± 0.0000*	*1.0000 ± 0.0000*	*1.8059 ± 0.0000*	*1.0000 ± 0.0000*

### Comparison with random sampling

A central question for any data-reduction method is whether its selection rule actually adds value over the trivial baseline of keeping a uniformly random subset of the same size. We answer this directly with a *matched-budget* protocol: at each budget the SSFSS coreset $(\mathcal {S},\boldsymbol{\gamma })$ and a random two-stage subsample retain exactly the same number of training points, so any performance gap is attributable to *which* samples are chosen rather than *how many*. Table 17 reports both methods averaged over the four differentiable ERM classifiers and three seeds. SSFSS is superior in all 36 head-to-head comparisons—three datasets *× * three budgets *× * four metrics—without a single exception, and the margin widens systematically as the metric becomes more sensitive to class imbalance. On Edge-IIoTset the gap is uniform and large at every budget: 11.3–12.0 points of accuracy, 11.7–12.3 points of weighted F1, 14.6–15.2 points of macro-F1, and 14.4–15.3 points of balanced accuracy. On CICIoT2023 the weighted metrics improve by roughly 5–6.5 points while the unweighted ones improve by 11.1–13.6. On RT-IoT2022 the weighted-metric margins are smaller—about 0.9–1.2 points—because both methods operate near the accuracy ceiling set by a dataset whose empirical risk is dominated by one dense class; the imbalance-sensitive margins, however, remain large throughout, from 11.7 points of macro-F1 and 13.5 points of balanced accuracy at the *5%* budget down to a still-substantial 7.3 and 8.6 points at the *15%* budget.

This separation between the weighted and unweighted metrics is precisely what one expects if random sampling degrades minority-class coverage. Accuracy and weighted F1 are dominated by the majority attack and benign classes, which any sufficiently large subsample represents adequately. Macro-F1 and balanced accuracy, by contrast, weight every class equally and are therefore governed by the rare classes, which a uniform draw under-samples in proportion to their scarcity and, for the rarest classes, may omit from a given seed entirely. Per-class Stage 2 selection removes this failure mode by construction rather than in expectation: the budget is allocated to every class independently and the Voronoi weights of class *c* sum to $n_c$, so the effective mass of each attack class is preserved exactly ($\rho _c^{\text {w}} = 1$, Section “Multiclass attack detection performance”), while the greedy maximization of the per-class coverage objective (8) minimizes the residual coverage error $\varepsilon _{\text {samp}}^{(c)}$ that enters the joint bound of Theorem 4.3 class by class. Uniform sampling offers neither guarantee: it preserves class proportions only in expectation and provides no control at all over within-class geometry. This is the mechanism behind the double-digit macro-F1 and balanced-accuracy gains on the two large, highly imbalanced corpora, where SSFSS lifts macro-F1 from 83.1 to 97.7–98.3 on Edge-IIoTset and from the low-to-mid 70s into the high 80s on CICIoT2023.

Two further observations reinforce the deployment case. First, SSFSS is markedly more *stable* than random sampling: its seed-to-seed dispersion is smaller by up to more than an order of magnitude (e.g., at the *5%* budget, *± 0.69* versus *± 8.98* accuracy points and *± 0.90* versus *± 7.92* macro-F1 points on CICIoT2023, and *± 0.11* versus *± 3.39* accuracy points on Edge-IIoTset), because an informed, per-class selection does not gamble on whether a given random draw happens to include the rare classes. For an intrusion-detection system that is retrained repeatedly in the field, this reliability is as important as the mean improvement. Second, random subsampling scales poorly with budget: tripling it from *5%* to *15%* buys only 0.2 points of accuracy on Edge-IIoTset, 0.3 on RT-IoT2022, and 1.0 on CICIoT2023, since the randomly drawn feature subset caps what any number of samples can deliver and additional uniform draws mostly duplicate already-covered majority regions. SSFSS, by contrast, is already at or near its ceiling at the tightest budget—its *5%* coreset outperforms a random subsample of *three times* the size on every metric and every dataset (e.g., 98.89 versus 87.10 accuracy on Edge-IIoTset and 94.92 versus 90.87 on CICIoT2023)—and continues to improve with the budget on CICIoT2023, the benchmark whose class geometry is least covered at *5%*, peaking at the *10%* budget ($94.92 \rightarrow 96.61 \rightarrow 96.51$ accuracy and $85.54 \rightarrow 88.29 \rightarrow 88.15$ macro-F1). Overall, the matched-budget comparison confirms that the benefits of SSFSS come from its selection rule rather than just data reduction, and this advantage is most significant in the class-imbalanced regime that characterizes realistic IoT intrusion detection.

**Table 17:** Matched-budget comparison of SSFSS against random two-stage subsampling, averaged over the four differentiable ERM classifiers (KLR, KSVM, KRR, SoftmaxLR; mean ± std over three seeds, in %).

Budget	Method	Accuracy	Weighted F1	Macro F1	Balanced Acc
*(a) RT-IoT2022*					
5%	Random	*98.21 ± 0.40*	*98.18 ± 0.40*	*79.65 ± 2.75*	*78.05 ± 3.17*
	SSFSS	$\mathbf {99.33 \pm 0.03}$	$\mathbf {99.33 \pm 0.04}$	$\mathbf {91.33 \pm 1.83}$	$\mathbf {91.56 \pm 2.03}$
10%	Random	*98.27 ± 0.39*	*98.20 ± 0.40*	*79.57 ± 3.68*	*77.87 ± 4.15*
	SSFSS	$\mathbf {99.36 \pm 0.06}$	$\mathbf {99.36 \pm 0.06}$	$\mathbf {88.97 \pm 0.63}$	$\mathbf {89.79 \pm 0.45}$
15%	Random	*98.51 ± 0.51*	*98.48 ± 0.52*	*82.32 ± 3.78*	*80.70 ± 4.38*
	SSFSS	$\mathbf {99.41 \pm 0.04}$	$\mathbf {99.41 \pm 0.04}$	$\mathbf {89.65 \pm 2.39}$	$\mathbf {89.28 \pm 2.23}$
*(b) Edge-IIoTset*					
5%	Random	*86.86 ± 3.39*	*86.58 ± 3.43*	*83.06 ± 3.09*	*82.53 ± 3.02*
	SSFSS	$\mathbf {98.89 \pm 0.11}$	$\mathbf {98.88 \pm 0.11}$	$\mathbf {98.27 \pm 0.20}$	$\mathbf {97.87 \pm 0.30}$
10%	Random	*87.05 ± 3.56*	*86.78 ± 3.62*	*83.10 ± 3.52*	*82.74 ± 3.30*
	SSFSS	$\mathbf {98.58 \pm 0.39}$	$\mathbf {98.58 \pm 0.39}$	$\mathbf {97.93 \pm 0.46}$	$\mathbf {97.66 \pm 0.53}$
15%	Random	*87.10 ± 3.48*	*86.73 ± 3.58*	*83.15 ± 3.42*	*82.85 ± 3.21*
	SSFSS	$\mathbf {98.39 \pm 0.45}$	$\mathbf {98.40 \pm 0.44}$	$\mathbf {97.73 \pm 0.60}$	$\mathbf {97.28 \pm 0.75}$
*(c) CICIoT2023*					
5%	Random	*89.85 ± 8.98*	*89.22 ± 9.52*	*71.94 ± 7.92*	*71.76 ± 7.56*
	SSFSS	$\mathbf {94.92 \pm 0.69}$	$\mathbf {94.87 \pm 0.75}$	$\mathbf {85.54 \pm 0.90}$	$\mathbf {85.33 \pm 0.91}$
10%	Random	*90.51 ± 8.57*	*89.97 ± 9.12*	*74.65 ± 7.62*	*74.41 ± 7.11*
	SSFSS	$\mathbf {96.61 \pm 0.57}$	$\mathbf {96.52 \pm 0.62}$	$\mathbf {88.29 \pm 0.61}$	$\mathbf {87.90 \pm 0.60}$
15%	Random	*90.87 ± 8.70*	*90.26 ± 9.27*	*77.09 ± 7.09*	*76.56 ± 7.37*
	SSFSS	$\mathbf {96.51 \pm 0.72}$	$\mathbf {96.42 \pm 0.78}$	$\mathbf {88.15 \pm 0.64}$	$\mathbf {87.68 \pm 0.84}$

At each budget (5%, 10%, 15% of the training samples), both methods retain the same number of samples, so differences isolate the effect of the SSFSS selection over uniform random sampling. The best value per metric within each budget is shown in bold.

### Comparison with state-of-the-art feature and sample selection methods

We evaluate SSFSS against established feature and sample selection methods using a controlled protocol: each baseline is assessed with the identical SoftmaxLR classifier to ensure that variations are attributable to the selection strategy rather than the learner. Table 18 first evaluates the feature stage in isolation, contrasting LA-CGFS with three conventional top-20 selectors: mutual information (MI), the ANOVA *F*-test, and PCA. LA-CGFS achieves the optimal value for every metric across all three datasets. The univariate filters deteriorate sharply when feature interactions are pertinent, with ANOVA yielding an accuracy of *82.03%* and a macro-F1 score of only *68.81%* on CICIoT2023, whereas the unsupervised PCA projection remains competitive on Edge-IIoTset (accuracy *97.19± 2.13* and macro-F1 *95.25± 3.24*) but at the cost of the highest variance among the filters. LA-CGFS attains the best macro-F1 across all three benchmarks (*88.63%*, *98.67%*, and *90.42%*), with tight dispersion on the two large corpora (macro-F1 std of 0.05 on Edge-IIoTset and 0.67 on CICIoT2023); the higher spread on RT-IoT2022 (*± 3.33*) reflects the handful of sub-30-sample classes that inflate the macro variance for *every* selector on that benchmark. These results validate that the supervised, conditionally greedy criterion of LA-CGFS identifies discriminative, non-redundant features overlooked by marginal-relevance filters and variance-based projections.

Table 19 broadens the comparison to the full two-stage framework at a *10%* sample budget, pairing each first-stage selector with random, stratified, or *k*-center second-stage sampling and evaluating all nine decoupled combinations against SSFSS-10. SSFSS-10 attains the optimal value across all four metrics on all three datasets. The critical observation is not the performance of any individual configuration—several baselines are locally competitive, such as PCA*+k*-center on RT-IoT2022 (*99.25%* accuracy)—but rather the absence of any baseline that stays competitive *across* datasets. The strongest non-SSFSS pipeline changes with the corpus (PCA*+k*-center on RT-IoT2022, MI+random on Edge-IIoTset, and MI-based sampling on CICIoT2023), and each off-the-shelf family breaks down somewhere: *k*-center sampling is fragile under class imbalance (MI*+k*-center reaches only *86.53%* accuracy on Edge-IIoTset, and on CICIoT2023 the *k*-center pairings collapse to *68.97%* for ANOVA*+k*-center and *77.74%* for PCA*+k*-center), while ANOVA-based pipelines fail on CICIoT2023 (*67.04%* macro-F1 for ANOVA+random). The SSFSS-10 advantage is most pronounced on the imbalance-sensitive metrics, improving macro-F1 over the strongest non-SSFSS baseline by as much as 6.6 points (*91.31%* vs. *84.75%* on CICIoT2023) and balanced accuracy by up to 6.9 points (*91.26%* vs. *84.39%*). It couples these gains with low, consistent seed-to-seed variance: on CICIoT2023 its macro-F1 dispersion (*± 0.37*) is roughly a third of that of its nearest macro competitor, MI+random (*± 1.01*), so the advantage does not come at the price of reliability.

This consistency arises directly from the pipeline’s architecture: integrating a supervised, coverage-aware Stage 1 with a submodular weighted coreset in Stage 2 preserves the minority-class structure that decoupled selector–sampler combinations overlook, yielding a reduced problem that is accurate, stable, and robust across diverse IoT traffic.

**Table 18:** First-stage feature-selection comparison on the top-20 features, using the SoftmaxLR classifier (mean ± std over three seeds, in %).

Selector	Accuracy	Weighted F1	Macro F1	Balanced Acc
*(a) RT-IoT2022*				
MI	*97.21 ± 0.04*	*97.36 ± 0.03*	*73.22 ± 0.38*	*86.41 ± 1.07*
ANOVA	*98.67 ± 0.15*	*98.92 ± 0.13*	*79.92 ± 1.34*	*84.80 ± 0.81*
PCA	*99.10 ± 0.05*	*99.15 ± 0.05*	*88.49 ± 0.38*	*85.94 ± 1.08*
LA-CGFS	$\mathbf {99.28 \pm 0.12}$	$\mathbf {99.25 \pm 0.13}$	$\mathbf {88.63 \pm 3.33}$	$\mathbf {86.46 \pm 4.09}$
*(b) Edge-IIoTset*				
MI	*98.74 ± 0.06*	*98.74 ± 0.06*	*98.11 ± 0.11*	*97.67 ± 0.13*
ANOVA	*96.42 ± 0.13*	*96.41 ± 0.13*	*95.48 ± 0.20*	*94.86 ± 0.21*
PCA	*97.19 ± 2.13*	*97.15 ± 2.18*	*95.25 ± 3.24*	*94.70 ± 3.40*
LA-CGFS	$\mathbf {99.28 \pm 0.08}$	$\mathbf {99.28 \pm 0.07}$	$\mathbf {98.67 \pm 0.05}$	$\mathbf {98.16 \pm 0.08}$
*(c) CICIoT2023*				
MI	*98.47 ± 0.11*	*98.41 ± 0.12*	*89.73 ± 0.61*	*89.43 ± 0.48*
ANOVA	*82.03 ± 0.06*	*79.30 ± 0.05*	*68.81 ± 1.01*	*69.26 ± 0.96*
PCA	*93.19 ± 0.11*	*93.10 ± 0.13*	*85.66 ± 0.56*	*84.95 ± 0.29*
LA-CGFS	$\mathbf {98.58 \pm 0.09}$	$\mathbf {98.52 \pm 0.11}$	$\mathbf {90.42 \pm 0.67}$	$\mathbf {89.67 \pm 0.75}$

All selectors reduce each dataset to 20 features; LA-CGFS is the proposed Stage 1 selector. The best value per metric within each dataset is shown in bold.

**Table 19:** Two-stage feature–sample selection comparison at a *10%* sample budget, using the SoftmaxLR classifier (mean ± std over three seeds, in %).

Variant	Accuracy	Weighted F1	Macro F1	Balanced Acc
*(a) RT-IoT2022*				
MI + random	*97.14 ± 0.03*	*97.21 ± 0.09*	*70.99 ± 3.32*	*77.35 ± 4.70*
MI + stratified	*97.26 ± 0.12*	*97.31 ± 0.10*	*76.42 ± 1.25*	*85.36 ± 0.60*
MI + *k*-center	*95.78 ± 0.02*	*95.93 ± 0.05*	*71.51 ± 0.67*	*80.60 ± 3.74*
ANOVA + random	*98.88 ± 0.17*	*98.97 ± 0.09*	*80.72 ± 4.51*	*86.72 ± 4.87*
ANOVA + stratified	*98.61 ± 0.05*	*98.86 ± 0.04*	*83.94 ± 2.39*	*93.94 ± 0.90*
ANOVA + *k*-center	*88.49 ± 0.23*	*91.85 ± 1.08*	*74.86 ± 3.59*	*86.91 ± 3.58*
PCA + random	*98.98 ± 0.09*	*99.03 ± 0.07*	*81.91 ± 5.28*	*85.87 ± 5.99*
PCA + stratified	*99.06 ± 0.09*	*99.08 ± 0.09*	*89.57 ± 1.30*	*92.65 ± 0.28*
PCA + *k*-center	*99.25 ± 0.09*	*99.28 ± 0.09*	*88.55 ± 1.82*	*93.61 ± 0.65*
**SSFSS-10**	$\mathbf {99.49 \pm 0.06}$	$\mathbf {99.49 \pm 0.06}$	$\mathbf {93.59 \pm 1.54}$	$\mathbf {94.16 \pm 0.76}$
*(b) Edge-IIoTset*				
MI + random	*98.00 ± 0.12*	*98.00 ± 0.13*	*97.12 ± 0.29*	*96.71 ± 0.41*
MI + stratified	*97.82 ± 0.12*	*97.81 ± 0.11*	*96.88 ± 0.24*	*96.27 ± 0.57*
MI + *k*-center	*86.53 ± 3.67*	*86.05 ± 3.91*	*80.79 ± 2.88*	*79.33 ± 3.04*
ANOVA + random	*95.81 ± 0.07*	*95.79 ± 0.07*	*94.70 ± 0.19*	*94.17 ± 0.08*
ANOVA + stratified	*95.59 ± 0.08*	*95.57 ± 0.10*	*94.43 ± 0.28*	*93.82 ± 0.24*
ANOVA + *k*-center	*92.47 ± 0.21*	*92.21 ± 0.16*	*85.87 ± 0.72*	*84.71 ± 0.80*
PCA + random	*97.11 ± 1.13*	*97.08 ± 1.17*	*94.88 ± 2.35*	*94.37 ± 2.47*
PCA + stratified	*97.53 ± 0.78*	*97.53 ± 0.78*	*95.34 ± 1.95*	*95.01 ± 1.78*
PCA + *k*-center	*92.94 ± 1.27*	*92.92 ± 1.23*	*89.97 ± 2.59*	*89.90 ± 2.37*
**SSFSS-10**	$\mathbf {99.18 \pm 0.25}$	$\mathbf {99.18 \pm 0.25}$	$\mathbf {98.73 \pm 0.35}$	$\mathbf {98.37 \pm 0.44}$
*(c) CICIoT2023*				
MI + random	*96.59 ± 0.10*	*96.52 ± 0.11*	*84.75 ± 1.01*	*84.39 ± 1.38*
MI + stratified	*97.47 ± 0.04*	*97.39 ± 0.05*	*84.16 ± 0.67*	*83.70 ± 0.83*
MI + *k*-center	*86.66 ± 0.48*	*86.12 ± 0.51*	*82.66 ± 0.71*	*82.26 ± 0.73*
ANOVA + random	*81.28 ± 0.23*	*78.71 ± 0.29*	*67.04 ± 1.10*	*67.51 ± 1.10*
ANOVA + stratified	*81.27 ± 0.08*	*78.67 ± 0.10*	*67.37 ± 0.21*	*67.94 ± 0.31*
ANOVA + *k*-center	*68.97 ± 3.07*	*65.94 ± 3.58*	*65.41 ± 1.28*	*67.49 ± 0.72*
PCA + random	*92.01 ± 0.18*	*91.92 ± 0.16*	*81.18 ± 0.96*	*81.23 ± 1.19*
PCA + stratified	*92.09 ± 0.15*	*92.01 ± 0.15*	*81.52 ± 0.52*	*80.89 ± 0.67*
PCA + *k*-center	*77.74 ± 2.14*	*76.19 ± 2.51*	*76.62 ± 1.35*	*77.15 ± 0.98*
**SSFSS-10**	$\mathbf {97.51 \pm 0.38}$	$\mathbf {97.50 \pm 0.38}$	$\mathbf {91.31 \pm 0.37}$	$\mathbf {91.26 \pm 0.42}$

Each baseline pairs a first-stage selector (MI, ANOVA, PCA on the top-20 features) with a second-stage sampler (random, stratified, or *k*-center); SSFSS-10 is the proposed pipeline (LA-CGFS + submodular coreset). The best value per metric within each dataset is shown in bold.

### Training-time and end-to-end speedup analysis

The primary objective of SSFSS at the systems level is to render repeated retraining economically viable on limited hardware, making training latency a key metric for evaluation. We present two complementary metrics. The *training-only* speedup evaluates the expense of training a classifier on the SSFSS coreset $(\mathcal {S},\boldsymbol{\gamma })$ in comparison to training the identical classifier on the complete training dataset. The end-to-end speedup also incurs a one-time expense for constructing the reduced problem, which includes Stage 1 feature selection and Stage 2 coreset selection and weighting, thereby quantifying the cost of a single cold start. All timings are averaged across the four differentiable ERM classifiers listed in Table 1 (KLR, KSVM, KRR, and SoftmaxLR) and over three seeds, collected on a single workstation (Intel Core i7-10750H, 32 GB RAM); the analysis below is based on these averaged times as reported in Table 20.

A methodological point clarifies how these ratios are formed. For each dataset, all three budgets—SSFSS-5, SSFSS-10, and SSFSS-15—are evaluated *within a single run*: the full-data baseline and Stage 1 feature selection are measured once and shared across the three budgets, and the corresponding coreset pipelines are measured consecutively in the same run. This is why the **Full train** and **Stage 1** columns of Table 20 are identical across the three rows of each sub-table—they report a single co-measured baseline rather than three independent measurements. Each training-only and end-to-end speedup is therefore the ratio of this shared full-data time to the corresponding coreset or end-to-end time recorded in the same run, so the reported gains are ratios of co-measured quantities and are directly comparable across budgets for a given dataset. Absolute wall-clock times still depend on the shared CPU’s thermal state, cache occupancy, and background load, which vary between the three per-dataset runs; the absolute magnitudes in Table 20 should accordingly be read as representative rather than as constants comparable across datasets.

The stage decomposition in Table 20 indicates that Stage 2 coreset selection predominates the construction budget. Stage 1 feature selection (LA-CGFS, including FAISS indexing) requires at most about 1.6 seconds on any dataset, and Voronoi-weight construction is smaller still, well under one second (0.06–0.69 s). The bulk of the one-time cost is coreset selection, ranging from roughly 45 to 54 seconds on RT-IoT2022, 8 to 10 seconds on CICIoT2023, and 10 to 14 seconds on Edge-IIoTset. This expense is *classifier-independent* and, importantly, is incurred *once*: the weighted coreset is reused for every subsequent retraining. The persistent per-update expense of SSFSS is represented by the **Coreset train** column, which is well below the full-data baseline—by roughly *8× * to *70× * depending on the dataset and budget.

When evaluated independently, the training cost diminishes for every dataset and budget. We quantify the training-only speedup directly from Table 20 as the ratio of the shared full-data training time to the averaged coreset-training time. Under the most stringent budget, SSFSS-5 reduces training time by *70.1× * on RT-IoT2022, *36.9× * on Edge-IIoTset, and *27.3× * on CICIoT2023. The balanced SSFSS-10 configuration retains speedups of *24.6× *, *13.7× *, and *13.4× *, while the fidelity-oriented SSFSS-15 continues to provide *17.1× *, *8.4× *, and *8.9× *, respectively. The ordering is monotonic in the retained-sample fraction and aligns with the joint error/computation trade-off delineated in Section “Error analysis”: a smaller coreset requires less computation and facilitates faster training, albeit at the expense of a marginally weaker gradient-approximation bound.

The end-to-end column reflects the least favorable accounting, in which the full construction cost is charged against a *single* coreset training that never reuses the coreset. Even under this conservative view, the averaged end-to-end time remains below the full-data training time for every dataset and budget, giving end-to-end speedups of *6.9× */*5.5× */*4.7× * on RT-IoT2022, *17.9× */*9.4× */*6.3× * on Edge-IIoTset, and *17.0× */*10.1× */*7.2× * on CICIoT2023 for SSFSS-5/10/15. Because Stage 2 selection is now a small fraction of the full-data fit on Edge-IIoTset and CICIoT2023, even a single cold start on those corpora is already *6× *–*18× * cheaper than training once on all the data; on RT-IoT2022, whose Stage 2 selection is the most expensive of the three, the cold-start gain is a still-substantial *4.7× *–*6.9× *. SSFSS is therefore already cheaper than a single full-data fit, and every additional update widens the margin, since each subsequent retraining costs only the **Coreset train** time and inherits the larger training-only speedup reported above.

**Fig. 5:**
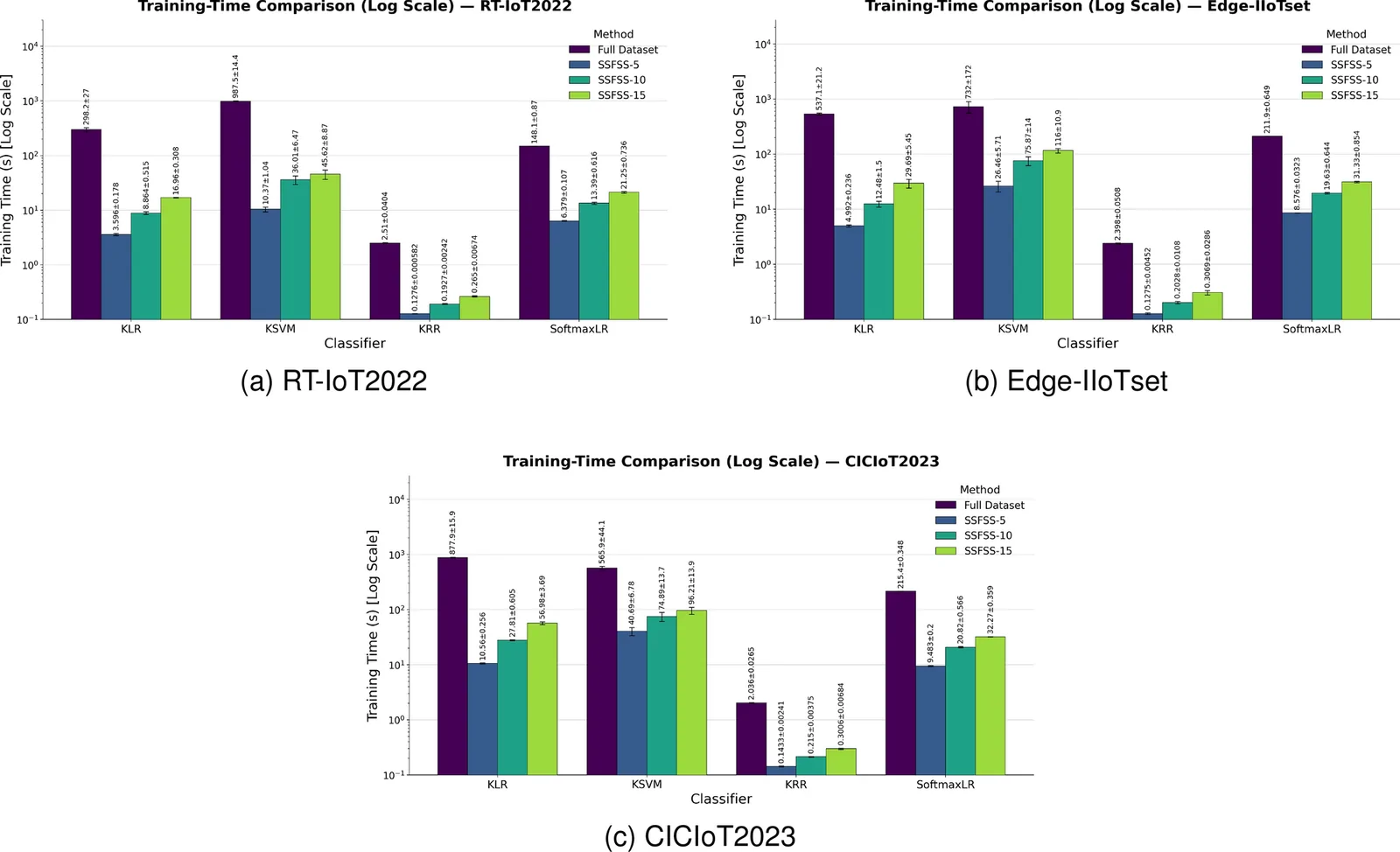
Log-scale training-time comparison across classifiers for the three benchmark datasets: (**a**) RT-IoT2022, (**b**) Edge-IIoTset, and (**c**) CICIoT2023.

Taken together, the two speedup views define three operating regimes. SSFSS-5 minimizes per-update latency and is preferable when retraining frequency dominates the cost model; SSFSS-15 is the most conservative reduced-data setting when fidelity is prioritized; and SSFSS-10 provides the best balance, preserving near-full accuracy (Tables 5, 6, 7, 8, 9, 10, 11, 12, 13, 14, 15, 16, 17, 18, 19, 20 and 21) while still compressing per-update training by more than an order of magnitude. The log-scale panels of Fig. 5 make the separation explicit: the reduced-data SSFSS bars sit consistently one to two decades below the full-data baseline, and the gap widens as the retained-sample fraction shrinks. These training-time gains are complemented by the peak-RAM reductions analyzed in Section “RAM usage reduction analysis”.

**Table 20:** SSFSS preprocessing and training time across the three datasets, averaged over the four differentiable ERM classifiers (mean ± std over three seeds, in seconds).

Variant	Full	Stage 1	Stage 2	Stage 2	Coreset	End-to-
	Train	(Select)	(Coreset)	(Weights)	Train	End
*(a) RT-IoT2022*						
SSFSS-5	*359.09 ± 10.59*	*1.57 ± 0.02*	*45.45 ± 0.15*	*0.22 ± 0.00*	*5.12 ± 0.33*	*52.37 ± 0.40*
SSFSS-10	*359.09 ± 10.59*	*1.57 ± 0.02*	*48.17 ± 1.11*	*0.46 ± 0.02*	*14.61 ± 1.90*	*64.83 ± 2.60*
SSFSS-15	*359.09 ± 10.59*	*1.57 ± 0.02*	*53.50 ± 0.55*	*0.69 ± 0.02*	*21.02 ± 2.48*	*76.80 ± 2.87*
*(b) Edge-IIoTset*						
SSFSS-5	*370.84 ± 48.44*	*0.63 ± 0.04*	*9.92 ± 0.32*	*0.08 ± 0.00*	*10.04 ± 1.50*	*20.68 ± 1.79*
SSFSS-10	*370.84 ± 48.44*	*0.63 ± 0.04*	*11.47 ± 0.55*	*0.14 ± 0.00*	*27.05 ± 4.03*	*39.30 ± 4.33*
SSFSS-15	*370.84 ± 48.44*	*0.63 ± 0.04*	*13.69 ± 0.56*	*0.21 ± 0.01*	*44.34 ± 4.31*	*58.88 ± 4.80*
*(c) CICIoT2023*						
SSFSS-5	*415.33 ± 15.08*	*0.94 ± 0.02*	*8.18 ± 0.18*	*0.06 ± 0.00*	*15.22 ± 1.81*	*24.42 ± 1.76*
SSFSS-10	*415.33 ± 15.08*	*0.94 ± 0.02*	*9.25 ± 0.28*	*0.11 ± 0.00*	*30.93 ± 3.71*	*41.25 ± 3.74*
SSFSS-15	*415.33 ± 15.08*	*0.94 ± 0.02*	*10.39 ± 0.33*	*0.17 ± 0.01*	*46.44 ± 4.50*	*57.95 ± 4.56*

The first column reports full-model training time on the complete (non-coreset) training set with the Stage 1 selected features, shown as a baseline. End-to-end time is the sum of Stage 1, Stage 2, and coreset training.

### RAM usage reduction analysis

Peak resident memory is the second binding constraint for on-device learning, since an edge gateway or embedded controller cannot retrain a model whose transient footprint exceeds its physical RAM. We therefore report peak RAM using the same protocol as the timing study: for each dataset, all three budgets are measured within a single run, peak RAM is captured per stage, and results are averaged over the four differentiable ERM classifiers (Table 1) and three seeds. The full-data peak and the Stage 1 peak are measured once and shared across the three budgets, which is why the **Full train** and **Stage 1** columns of Table 21 are identical across the three rows of each sub-table. Each reduction factor is thus the ratio of this shared full-data peak to the corresponding coreset-training peak recorded in the same run. As with wall-clock time, absolute footprints drift between the per-dataset runs with allocator and machine state, so the absolute values in Table 21 should be read as representative magnitudes, whereas the reduction factors below are ratios of co-measured peaks and are robust to inter-run drift.

The stage decomposition in Table 21 reveals a structural property that distinguishes the memory profile from the timing profile. The two selection stages are memory-light: Stage 1 (LA-CGFS) peaks at only a few MiB on Edge-IIoTset and CICIoT2023 (*≈ 3.3* MiB) and in the tens of MiB on RT-IoT2022 (40.7 MiB), and Stage 2 coreset construction is comparably small (*≈ 3.3*–25.7 MiB across datasets). Crucially, the three stages execute *sequentially* rather than concurrently, so the memory allocated by one stage is released before the next begins and is amortized by it; the peak resident set of the whole pipeline is therefore the *maximum* of the per-stage peaks, not their sum. This is the memory-side counterpart to the timing analysis, where the end-to-end *time* is additive because each stage’s duration accumulates. Consequently, the binding peak of the whole pipeline is the coreset-*training* step, and the end-to-end peak coincides with the coreset-training peak in all nine configurations (Table 21). In other words, whereas the one-time Stage 2 construction dominates the time budget, it is negligible in the memory budget, so SSFSS introduces essentially no preprocessing memory overhead, and the training-variant and end-to-end-variant reductions are identical.

The reductions relative to full-data training are substantial across all datasets and budgets. Under the most aggressive budget, SSFSS-5 lowers the average training peak from 874.8 MiB to 49.0 MiB on RT-IoT2022 (*17.9× *; *94.4%*), from 1070.6 MiB to 48.5 MiB on Edge-IIoTset (*22.1× *; *95.5%*), and from 974.6 MiB to 34.2 MiB on CICIoT2023 (*28.5× *; *96.5%*). The balanced SSFSS-10 configuration retains reductions of *8.6× *, *8.5× *, and *10.2× *, and the fidelity-oriented SSFSS-15 still delivers *5.9× *, *6.3× *, and *7.1× *, respectively. Because the end-to-end peak coincides with the coreset-training peak, the end-to-end variant achieves these same reduction factors, so the framework is memory-efficient not only at steady-state retraining but also for a single cold start.

Two trends are worth noting. First, the reduction factor decreases monotonically as the retained-sample fraction grows, consistent with the coreset-training peak scaling with the size of the reduced design matrix and the learner-side kernel structures built from it; the raw peaks in Table 21 grow with the budget on every dataset (for example, $49.0 \rightarrow 101.8 \rightarrow 147.5$ MiB on RT-IoT2022). Second, the largest reduction at a fixed budget occurs on CICIoT2023 (*28.5× * for SSFSS-5, against *17.9× * for RT-IoT2022), because a full-data fit on that high-cardinality corpus materializes a large footprint that a fixed-fraction coreset compresses most aggressively.

Taken together with the timing results, the memory analysis confirms the intended operating regime of SSFSS. The balanced SSFSS-10 coreset reduces average training and end-to-end peak RAM to 101.8, 126.5, and 95.1 MiB on RT-IoT2022, Edge-IIoTset, and CICIoT2023—close to an order-of-magnitude reduction (*8.6× *, *8.5× *, and *10.2× *) relative to the roughly 0.85–1.05 GiB full-data baselines—while SSFSS-5 pushes the compression to *18× *–*28× * (tens of MiB) when memory is the dominant constraint. Reducing the retraining footprint from the gigabyte scale of full-data training to roughly 100 MiB at SSFSS-10, and to the tens of MiB at SSFSS-5, is a substantial measured reduction that motivates deployment on resource-constrained gateways and embedded controllers; confirming on-device feasibility on such hardware is left to future work. These gains complement the training-time and end-to-end speedups of Section “Training-time and end-to-end speedup analysis”.

**Table 21:** SSFSS peak RAM usage across the three datasets, averaged over the four differentiable ERM classifiers (mean ± std over three seeds, in MiB).

Variant	Full train	Stage 1	Stage 2	Coreset train	End-to-end
*(a) RT-IoT2022*					
SSFSS-5	*874.84 ± 2.39*	*40.71 ± 4.63*	*22.76 ± 2.42*	*49.00 ± 2.55*	*49.00 ± 2.55*
SSFSS-10	*874.84 ± 2.39*	*40.71 ± 4.63*	*25.59 ± 0.72*	*101.83 ± 2.54*	*101.83 ± 2.54*
SSFSS-15	*874.84 ± 2.39*	*40.71 ± 4.63*	*25.67 ± 2.73*	*147.45 ± 2.71*	*147.45 ± 2.71*
*(b) Edge-IIoTset*					
SSFSS-5	*1070.58 ± 7.39*	*3.27 ± 0.05*	*3.30 ± 0.11*	*48.47 ± 0.06*	*48.47 ± 0.06*
SSFSS-10	*1070.58 ± 7.39*	*3.27 ± 0.05*	*3.37 ± 0.04*	*126.53 ± 0.06*	*126.53 ± 0.06*
SSFSS-15	*1070.58 ± 7.39*	*3.27 ± 0.05*	*3.29 ± 0.03*	*170.84 ± 0.10*	*170.84 ± 0.10*
*(c) CICIoT2023*					
SSFSS-5	*974.56 ± 1.50*	*3.36 ± 0.05*	*3.45 ± 0.04*	*34.22 ± 1.73*	*34.22 ± 1.73*
SSFSS-10	*974.56 ± 1.50*	*3.36 ± 0.05*	*3.47 ± 0.04*	*95.10 ± 1.45*	*95.10 ± 1.45*
SSFSS-15	*974.56 ± 1.50*	*3.36 ± 0.05*	*3.45 ± 0.03*	*137.27 ± 0.87*	*137.27 ± 0.87*

The first column reports full-model training peak RAM on the complete (non-coreset) training set with the Stage 1 selected features, shown as a baseline. End-to-end peak RAM is the maximum of the Stage 1, Stage 2, and coreset-training peaks, since the stages run sequentially and each stage’s memory is released before the next begins.

### Comparison with recent feature-sample reduction frameworks

To position SSFSS against the most closely related recent literature, Table 22 compares it with recent IoT/IIoT IDS frameworks that combine feature reduction with sample-side operations such as rebalancing, clustering, pruning, or explicit subset selection. Because these studies differ in datasets, task granularity, data splits, and model families, the table should be interpreted as a structured positioning analysis rather than as a strict leaderboard. To keep the comparison internally consistent, the table reports predictive feature counts only (excluding target/label columns), explicitly distinguishes sample compression from sample rebalancing, and reproduces the systems metric that each paper actually reports—relative training speedup when available, and otherwise the reported absolute runtime or memory figure.

Several patterns are clear. First, most recent frameworks still combine feature selection with oversampling or hybrid rebalancing rather than *true training-set compression*^12,19,35,36^. These pipelines can improve minority-class detection, but they usually retain or even enlarge the effective training set, which is not well aligned with the update-efficient learning objective emphasized throughout this paper. By contrast, Gheni and Al-Yaseen^14^ and SSFSS explicitly reduce the dataset size; however, only SSFSS couples that reduction with label-aware feature selection and a geometry-aware coreset selection objective targeted at retraining fidelity.

Second, SSFSS is the only framework in Table 22 that reports *full-dataset-relative* training speedups together with peak-RAM measurements across three modern IoT benchmarks. Under the balanced **SSFSS-10** operating point, SSFSS retains only 20 selected features and 10% of the training samples, while still achieving 99.49%, 99.18%, and 97.51% accuracy on RT-IoT2022, Edge-IIoTset, and CICIoT2023, respectively, with average training speedups of 24.6*× *, 13.7*× *, and 13.4*× * and peak training RAM of 101.8, 126.5, and 95.1 MiB. This operating point is precisely aligned with the update-efficient learning regime discussed in Sections “Training-time and end-to-end speedup analysis” and “RAM usage reduction analysis”.

Third, the comparison shows that strong raw accuracy alone does not fully characterize practical lightweight retraining. AegisGuard^19^ reports excellent accuracy, but it also reports 47.3 minutes of training time and 8.4 GB of memory usage, placing it in a materially heavier operating regime. Ayad et al.^35^ and Yang et al.^36^ obtain strong detection results, yet rely on sample rebalancing rather than compression and do not report retraining-cost reductions relative to full-data training. LIRAD^12^ is the closest in spirit to resource-aware deployment, but it reports absolute runtime and memory rather than speedup relative to the unreduced training set. The main value of SSFSS is therefore not only competitive fidelity, but a more favorable *accuracy–training cost–memory* trade-off for repeated edge-side retraining.

**Table 22:** Comparison of SSFSS with recent IoT/IIoT IDS frameworks that combine feature reduction with sample-side rebalancing, selection, or compression.

Framework	Feature reduction	Sample-side operation	Datasets	Model family	Best reported accuracy	Reported speedup/time	Reported memory	Key comparative note
Gheni and Al-Yaseen^14^	Information Gain(47*→ *15)	Two-step GSK clustering(87,438 retained rows;37.55% retained)	CICIoT2023	MLP, AE	97.46% multiclass(99.26% binary)	0.03 s(test time)	N/R	Performs dataset compression, but no retraining-speed or RAM study
Ayad et al.^35^	Hybrid filter+wrapper(46*→ *12; 45*→ *15;79*→ *35)	SMOTE oversampling(rebalancing; ratio N/R)	BoT-IoT;TON-IoT;CIC-DDoS2019	DT, RF,GNB, KNN	99.82–100%	0.02–0.15 s(detection time)	N/R	Strong accuracy, but expands/rebalances rather than compresses the training set
Yang et al.^36^	RF-P(60*→ *39; 79*→ *61;46*→ *35)	ARL hybrid rebalancing(ADASYN + RENN + LOF;ratio N/R)	Edge-IIoTset;CICIDS2017;CICIoT2023	BiGRU + attention+ Inception-CNN	94.70–99.50%	N/R	N/R	Strong deep model on modern datasets, but no explicit retraining-cost analysis
Mishra et al.^12^	Boruta + WOA	GA-SMOTE, SMOTE-PSO,RUS + tree pruning	CICIDS2017;EDGE-IIOT	DT, RF,XGBoost	99.50–99.99%	4.09–4.26 s(execution time)	1.93–3.07 MB	Resource-aware deployment study, but no full-data-relative training speedup
Abou Elasaad et al.^19^	QIFSA(41*→ *12 to 44*→ *13;70.6% avg. reduction)	Four-stage rebalancing(SMOTE + SMOTE-ENN + ADASYN+ controlled undersampling)	CICIoT2023;IoT-Intrusion;RT-IoT2022;X-IIoTID	Calibratedensemble	99.68–99.74%	47.3 min(training time)	8.4 GB	Excellent accuracy, but substantially heavier training footprint
**SSFSS (this work)**	**LA-CGFS** **(20 selected features)**	**Geometry-aware Coreset Selection** **selection (10% retained samples)**	**RT-IoT2022;** **Edge-IIoTset;** **CICIoT2023**	**KLR, KSVM,** **KRR, SoftmaxLR**	**97.51–99.49%**	**13.4** *× * **–24.6** *× * **(training speedup)**	**95.1–126.5 MiB**	**True feature and sample compression; explicit retraining-speed and RAM study**

The proposed SSFSS framework is shown in bold.

*Notes:* Feature counts exclude target/label columns. “N/R” denotes “not reported” in the original paper. The accuracy column reproduces the best result explicitly reported under each paper’s own evaluation protocol; accordingly, some entries reflect best multiclass results, while Gheni and Al-Yaseen also report a binary result that is shown in parentheses. In the speed/time column, relative speedup is reported when available; otherwise, the paper’s reported absolute runtime is shown. For oversampling and hybrid rebalancing methods, a retained-sample ratio is typically not defined because the training set is modified rather than compressed. For SSFSS, the table uses the balanced **SSFSS-10** operating point from Tables 5, 6, 7, 8, 9, 10, 11, 12, 13, 14, 15, 16, 17, 18, 19, 20 and 21. The fidelity-oriented **SSFSS-15** variant yields slightly higher accuracies on RT-IoT2022 (99.56%) and CICIoT2023 (97.62%) and is essentially unchanged on Edge-IIoTset (99.16%), with smaller but still substantial training-speed and RAM gains.

Taken together, Table 22 reinforces the central claim of this paper. Prior works either improve predictive quality through sample rebalancing, reduce dataset size without quantifying the resulting retraining benefits, or report resource-aware deployment metrics without relating them to unreduced full-data training. SSFSS occupies a distinct position in this literature by jointly compressing the feature and sample spaces and by directly showing how that compression translates into large retraining-time and RAM savings while preserving high multiclass detection fidelity on modern IoT benchmarks.

## Conclusion and future work

This paper introduced the Sequential Submodular Feature-Sample Selection (SSFSS) framework for update-efficient IoT intrusion detection under tight edge-side computational and memory constraints. By coupling Label-Aware Coreset Greedy-Based Feature Selection (LA-CGFS) with geometry-aware sample coreset construction, SSFSS performs sample compression in a representation space that already preserves class-discriminative structure. From the theoretical perspective, both stages are formulated as cardinality-constrained monotone submodular maximization problems and therefore inherit the classical greedy *(1-1/e)* approximation guarantee at the stage level. The unified analysis further shows that the discrepancy between full-data and reduced-data training is controlled by feature distortionand sample-coverage error in the reduced space.

The empirical study confirms that this sequential design is effective across RT-IoT2022, Edge-IIoTset, and CICIoT2023. SSFSS reduces the representation to 20 selected features and compresses the training set to 5%–15% of the original training split. Among the evaluated budgets, **SSFSS-10** provides the most balanced operating point: the best reduced classifier on each dataset attains 99.49%, 99.18%, and 97.51% accuracy on RT-IoT2022, Edge-IIoTset, and CICIoT2023, respectively, while the average training speedup reaches 24.6*× *, 13.7*× *, and 13.4*× *, and the peak training RAM drops to 101.8, 126.5, and 95.1 MiB, corresponding to approximately 88.4%, 88.2%, and 90.2% reductions relative to full-data training. When fidelity is prioritized, **SSFSS-15** raises the best reduced accuracy to 99.56% on RT-IoT2022 and 97.62% on CICIoT2023 and holds it at 99.16% on Edge-IIoTset, while still maintaining substantial efficiency gains. When maximum efficiency is prioritized, **SSFSS-5** increases the average training speedup to 70.1*× *, 36.9*× *, and 27.3*× *, with peak-RAM reduction factors of 17.9*× *, 22.1*× *, and 28.5*× *, respectively; even with the one-time coreset-construction overhead included, the end-to-end pipeline stays faster than a single full-data fit on every dataset and budget.

At the same time, the results make the operating trade-off explicit. The strongest performance retention is obtained for the hinge- and softmax-loss ERMs (KSVM and SoftmaxLR), for which 10–15% coresets remain within roughly half a point of full-data support-weighted performance on Edge-IIoTset and within about one point on CICIoT2023, and exceed it on RT-IoT2022; KLR shows the largest support-weighted degradation under compression, whereas the squared-loss KRR is the weakest learner on full data but benefits the most from the weighted coreset’s rebalancing effect on the minority-sensitive metrics. The per-class analysis also indicates that the label-aware feature stage helps preserve minority-class structure better than naive global reduction; however, extremely low-support or intrinsically difficult classes remain the most sensitive to aggressive compression. SSFSS should therefore be interpreted not as a lossless reduction method for every classifier, but as a principled and practically effective way to optimize the accuracy–training time–memory trade-off required for frequent retraining on resource-constrained IoT platforms.

Future work will extend SSFSS beyond the present batch setting to streaming and concept-drifting environments, where the selected coreset must be updated incrementally rather than recomputed from scratch. Another promising direction is federated and distributed edge learning, in which transmitting compact representative subsets instead of full local datasets could reduce communication overhead while preserving training fidelity. A further essential direction is validation on representative embedded and edge hardware, such as microcontroller- and gateway-class platforms: the efficiency gains reported here are measured on a workstation, and confirming them on constrained devices is necessary to fully substantiate the on-device deployment motivation of this work.

## Data Availability

The datasets and the practical code used during the current study are available from the corresponding author [Mohammed Nagah Amr, Moh.Nagah@ngu.edu.eg] on reasonable request. Datasets links: 1) CIC-IoT2023: https://www.kaggle.com/ datasets/himadri07/ciciot2023, 2) RT-IoT2022: https://archive.ics.uci.edu/dataset/942/rt-iot2022, and 3) Edge-IIoT: https://www.kaggle.com/datasets/sibasispradhan/edge-iiotset-dataset?select= ML-EdgeIIoT-dataset.csv

## Appendix 1: Proofs supporting the error analysis

This appendix proves the three main results used in the error analysis of SSFSS. The proof of Theorem 4.1 is given in Appendix A.1, the proof of Theorem 4.2 is given in Appendix A.2, and the proof of Theorem 4.3 is given in Appendix A.3. Throughout, we use the notation introduced in Section “Error analysis”, including the full-data gradient (10), the projected full-data gradient (4), the weighted coreset gradient (5), the per-class assignment map (16), and the decomposition in (13).

### A.1 Proof of Theorem 4.1

#### Proof

By the definition of $\Delta _{\text {feat}}(F_s)$ in (13), together with (10) and (4), we have

$$ \Delta _{\text {feat}}(F_s) = \sup _{\boldsymbol{\theta }\in \Theta } \left\| \frac{1}{n} \sum _{i=1}^{n} \left[ \nabla _{\boldsymbol{\theta }}\ell (\boldsymbol{\theta };\boldsymbol{x}_i,y_i) - \nabla _{\boldsymbol{\theta }}\ell (\boldsymbol{\theta };\boldsymbol{x}_i^{(F_s)},y_i) \right] \right\| _2 $$

Applying the triangle inequality gives

$$ \Delta _{\text {feat}}(F_s) \le \frac{1}{n} \sum _{i=1}^{n} \sup _{\boldsymbol{\theta }\in \Theta } \left\| \nabla _{\boldsymbol{\theta }}\ell (\boldsymbol{\theta };\boldsymbol{x}_i,y_i) - \nabla _{\boldsymbol{\theta }}\ell (\boldsymbol{\theta };\boldsymbol{x}_i^{(F_s)},y_i) \right\| _2 $$

Each summation compares two gradients evaluated at the *same* label $y_i$, so the label-conditional Lipschitz condition (12) applies directly and yields

$$ \Delta _{\text {feat}}(F_s) \le \frac{L_{\text {in}}}{n} \sum _{i=1}^{n} \left\| \boldsymbol{x}_i-\boldsymbol{x}_i^{(F_s)} \right\| _2 = L_{\text {in}}\,\varepsilon _{\text {feat}}(F_s) $$

This proves (15). *□ *

### A.2 Proof of Theorem 4.2

#### Proof

Since the class index sets $\mathcal {I}_1,\dots ,\mathcal {I}_C$ partition $\mathcal {I}$, the projected full-data gradient (4) can be written class-wise as $\boldsymbol{g}_{\text {full}}^{(F_s)}(\boldsymbol{\theta }) = \frac{1}{n}\sum _{c=1}^{C}\sum _{i\in \mathcal {I}_c}\nabla _{\boldsymbol{\theta }}\ell (\boldsymbol{\theta };\boldsymbol{x}_i^{(F_s)},y_i)$. Combining this with the assignment form of the coreset gradient (17), the definition of $\Delta _{\text {samp}}(\mathcal {S},\boldsymbol{\gamma }\mid F_s)$ in (13) becomes

$$ \Delta _{\text {samp}}(\mathcal {S},\boldsymbol{\gamma }\mid F_s) = \sup _{\boldsymbol{\theta }\in \Theta } \left\| \frac{1}{n} \sum _{c=1}^{C} \sum _{i\in \mathcal {I}_c} \left[ \nabla _{\boldsymbol{\theta }}\ell (\boldsymbol{\theta };\boldsymbol{x}_i^{(F_s)},y_i) - \nabla _{\boldsymbol{\theta }}\ell (\boldsymbol{\theta };\boldsymbol{x}_{a_c(i)}^{(F_s)},y_i) \right] \right\| _2 $$

Applying the triangle inequality gives

$$ \Delta _{\text {samp}}(\mathcal {S},\boldsymbol{\gamma }\mid F_s) \le \frac{1}{n} \sum _{c=1}^{C} \sum _{i\in \mathcal {I}_c} \sup _{\boldsymbol{\theta }\in \Theta } \left\| \nabla _{\boldsymbol{\theta }}\ell (\boldsymbol{\theta };\boldsymbol{x}_i^{(F_s)},y_i) - \nabla _{\boldsymbol{\theta }}\ell (\boldsymbol{\theta };\boldsymbol{x}_{a_c(i)}^{(F_s)},y_i) \right\| _2 $$

Because Stage 2 selects each coreset inside a single class, the assignment (16) satisfies $a_c(i)\in \mathcal {S}_c\subseteq \mathcal {I}_c$ and hence $y_{a_c(i)}=y_i$ for every *i*. Every summand therefore compares two gradients evaluated at the same label, and the label-conditional Lipschitz condition (12) applies to each of them, giving

$$ \Delta _{\text {samp}}(\mathcal {S},\boldsymbol{\gamma }\mid F_s) \le \frac{L_{\text {in}}}{n} \sum _{c=1}^{C} \sum _{i\in \mathcal {I}_c} \left\| \boldsymbol{x}_i^{(F_s)}-\boldsymbol{x}_{a_c(i)}^{(F_s)} \right\| _2 $$

Finally, using the per-class coverage error (18) together with the aggregation identity (19), we obtain

$$ \Delta _{\text {samp}}(\mathcal {S},\boldsymbol{\gamma }\mid F_s) \le L_{\text {in}} \sum _{c=1}^{C} \frac{n_c}{n}\, \varepsilon _{\text {samp}}^{(c)}(\mathcal {S}_c\mid F_s) = L_{\text {in}}\,\varepsilon _{\text {samp}}(\mathcal {S}\mid F_s) $$

This proves (21). *□ *

### A.3 Proof of Theorem 4.3

#### Proof

Starting from the decomposition (13) and applying Theorems 4.1 and 4.2, we obtain

$$ \mathcal {E}_{\text {total}} \le \Delta _{\text {feat}}(F_s) + \Delta _{\text {samp}}(\mathcal {S},\boldsymbol{\gamma }\mid F_s) $$

$$ \mathcal {E}_{\text {total}} \le L_{\text {in}}\,\varepsilon _{\text {feat}}(F_s) + L_{\text {in}} \sum _{c=1}^{C} \frac{n_c}{n}\, \varepsilon _{\text {samp}}^{(c)}(\mathcal {S}_c\mid F_s) $$

Collecting the common factor $L_{\text {in}}$ gives

$$ \mathcal {E}_{\text {total}} \le L_{\text {in}} \left[ \varepsilon _{\text {feat}}(F_s) + \sum _{c=1}^{C} \frac{n_c}{n}\, \varepsilon _{\text {samp}}^{(c)}(\mathcal {S}_c\mid F_s) \right] $$

This is exactly (22). *□ *

## References

[1] Aivaliotis, V., Tsantikidou, K. & Sklavos, N. IoT-based multi-sensor healthcare architectures and a lightweight-based privacy scheme. *Sensors* **22**, 4269 (2022). 35684890 10.3390/s22114269 PMC9185436

[2] Sharmila, B. & Nagapadma, R. Quantized autoencoder (QAE) intrusion detection system for anomaly detection in resource-constrained IoT devices using RT-IoT2022 dataset. *Cybersecurity* **6**, 41 (2023).

[3] Insights, T. *Global IoT connections to hit 29.4 billion in* 2030 (2022).

[4] Ferrag, M. A., Friha, O., Hamouda, D., Maglaras, L. & Janicke, H. Edge-IIoTset: A new comprehensive realistic cyber security dataset of IoT and IIoT applications for centralized and federated learning. *IEEE Access* **10**, 40281–40306 (2022).

[5] Neto, E. C. P. et al. CICIoT2023: A real-time dataset and benchmark for large-scale attacks in IoT environment. *Sensors* **23**, 5941 (2023). 37447792 10.3390/s23135941 PMC10346235

[6] Rahman, M. M., Al Shakil, S. & Mustakim, M. R. A survey on intrusion detection system in IoT networks. *Cyber Secur. Appl.* **3**, 100082 (2025).

[7] Rehman, M. U., Kalakoti, R. & Bahşi, H. Comprehensive feature selection for machine learning-based intrusion detection in healthcare IoMT networks. In *Proceedings of the 11th International Conference on Information Systems Security and Privacy*, 248–259. 10.5220/0013313600003899. organizationINSTICC (SciTePress, 2025).

[8] Elrawy, M. F., Awad, A. I. & Hamed, H. F. Intrusion detection systems for IoT-based smart environments: A survey. *J. Cloud Comput.* **7**, 1–20 (2018).

[9] Musthafa, M. B. et al. Optimizing IoT intrusion detection using balanced class distribution, feature selection, and ensemble machine learning techniques. *Sensors* **24**, 4293 (2024). 39001072 10.3390/s24134293 PMC11244377

[10] Qaddos, A. et al. A novel intrusion detection framework for optimizing IoT security. *Sci. Rep.* **14**, 21789 (2024). 39294195 10.1038/s41598-024-72049-z PMC11410947

[11] Prasad, A. et al. Optimizing IoT intrusion detection with cosine similarity based dataset balancing and hybrid deep learning. *Sci. Rep.* **15**, 30939 (2025). 40847039 10.1038/s41598-025-15631-3 PMC12373782

[12] Mishra, S. et al. LIRAD: Lightweight tree-based approaches on resource constrained IoT devices for attack detection. *Cluster Comput.* **28**, 140 (2025).

[13] Fusco, P., Rimoli, G., Guerriero, A., Palmieri, F. & Ficco, M. On-device training and pruning for energy saving and continuous learning in resource-constrained MCUs. *Future Gener. Comput. Syst.* 108194 (2025).

[14] Gheni, H. Q. & Al-Yaseen, W. L. Two-step data clustering for improved intrusion detection system using CICIoT2023 dataset. *e-Prime-Adv. Electr. Eng. Electron. Energy* **9**, 100673 (2024).

[15] Narayan, K. G. R. IIDS: Design of intelligent intrusion detection system for internet-of-things applications. In *2023 IEEE 7th Conference on Information and Communication Technology (CICT)* 1–6 (organizationIEEE, 2023).

[16] Amierh, Z., Hammad, L., Qaddoura, R., Al-Omari, H. & Faris, H. A multiclass classification approach for IoT intrusion detection based on feature selection and oversampling. In *Cyber Malware: Offensive and Defensive Systems*, 197–233 (Springer, 2023).

[17] Javed, A., Javaid, N., Imran, M., Nasser, N. & Ali, A. Towards accurate intrusion detection in IoT: A deep learning approach with optimization and active sample selection. In *GLOBECOM 2025-2025 IEEE Global Communications Conference*, 943–948 (organizationIEEE, 2025).

[18] Siddharthan, H. & Thangavel, D. A novel framework approach for intrusion detection based on improved critical feature selection in internet of things networks. *Concurr. Comput. Pract. Exp.* **35**, e7445 (2023).

[19] Abou Elasaad, M. M., Sayed, S. G. & El-Dakroury, M. M. AegisGuard: A multi-stage hybrid intrusion detection system with optimized feature selection for industrial IoT security. *Sensors* **25**, 6958 (2025). 41305165 10.3390/s25226958 PMC12655908

[20] Har-Peled, S. & Mazumdar, S. On coresets for k-means and k-median clustering. In *Proceedings of the Thirty-Sixth Annual ACM Symposium on Theory of Computing*, 291–300 (2004).

[21] Bachem, O., Lucic, M. & Krause, A. Scalable k-means clustering via lightweight coresets. In *Proceedings of the 24th ACM SIGKDD International Conference on Knowledge Discovery & Data Mining*, 1119–1127 (2018).

[22] Mirzasoleiman, B., Bilmes, J. & Leskovec, J. Coresets for data-efficient training of machine learning models. In *Proceedings of the 37th International Conference on Machine Learning (ICML)*, vol. 119 of *seriesProceedings of Machine Learning Research* (PMLR, 2020).

[23] Killamsetty, K., Durga, S., Ramakrishnan, G., De, A. & Iyer, R. Grad-match: Gradient matching based data subset selection for efficient deep model training. In *International Conference on Machine Learning*, 5464–5474 (organizationPMLR, 2021).

[24] Nemhauser, G. L., Wolsey, L. A. & Fisher, M. L. An analysis of approximations for maximizing submodular set functions-I. *Math. Program.* **14**, 265–294 (1978).

[25] Ghubaish, A., Yang, Z., Erbad, A. & Jain, R. LEMDA: A novel feature engineering method for intrusion detection in IoT systems. *IEEE Internet Things J.* **11**, 13247–13256 (2023).

[26] Houkan, A. Enhancing security in industrial IoT networks: Machine learning solutions for feature selection and reduction (IEEe Access, 2024).

[27] Huang, W. et al. GSLDWOA: A feature selection algorithm for intrusion detection systems in IIoT. *Comput. Mater. Contin.* **86**, 1–24 (2025).

[28] Seyedi, B. & Postolache, O. Securing IoT communications via anomaly traffic detection: Synergy of genetic algorithm and ensemble method. *Sensors* **25**, 4098 (2025). 40648351 10.3390/s25134098 PMC12252052

[29] Harahsheh, K., Al-Naimat, R. & Chen, C.-H. Using feature selection enhancement to evaluate attack detection in the internet of things environment. *Electronics* **13**, 1678 (2024).

[30] Soliman, S., Oudah, W. & Aljuhani, A. Deep learning-based intrusion detection approach for securing industrial internet of things. *Alex. Eng. J.* **81**, 371–383 (2023).

[31] Saikam, J. & Ch, K. EESNN: Hybrid deep learning empowered spatial-temporal features for network intrusion detection system. *IEEE Access* **12**, 15930–15945 (2024).

[32] Ma, Z., Yuan, L., Yu, Y. & Peng, X. Dynamic weight-based intrusion detection model for IoT integration. In *2023 5th International Conference on Artificial Intelligence and Computer Applications (ICAICA)*, 286–290 (organizationIEEE, 2023).

[33] Pestov, V. On the geometry of similarity search: dimensionality curse and concentration of measure (1999) cs/9901004 arXiv preprint.

[34] François, D., Wertz, V. & Verleysen, M. The concentration of fractional distances. *IEEE Trans. Knowl. Data Eng.* **19**, 873–886 (2007).

[35] Ayad, A. G., Sakr, N. A. & Hikal, N. A. A hybrid approach for efficient feature selection in anomaly intrusion detection for IoT networks. *J. Supercomput.* **80**, 26942–26984. 10.1007/s11227-024-06409-x (2024).

[36] Yang, K., Wang, J. & Li, M. An improved intrusion detection method for IIoT using attention mechanisms, BiGRU, and Inception-CNN. *Sci. Rep.* **14**, 19339. 10.1038/s41598-024-70094-2 (2024). 39164384 10.1038/s41598-024-70094-2 PMC11335856

